# Adoptive T-cell therapies for persistent COVID-19 in immunocompromised patients: Comparison of IFN-γ virus-specific T-cell therapy and CD45RA^+^ T-cell depleted donor lymphocyte infusion

**DOI:** 10.1007/s11357-025-02050-5

**Published:** 2026-01-12

**Authors:** László Gopcsa, Borisz Rabán Petrik, Bálint Gergely Szabó, Marienn Réti, Hajnalka Andrikovics, Ilona Bobek, Gabriella Bekő, Judit Bogyó, Andrea Ceglédi, Katalin Dobos, Laura Giba-Kiss, Orsolya Kis, Botond Lakatos, Dóra Mathiász, Nóra Meggyesi, Gottfried Miskolczi, Noémi Németh, János Sinkó, Anikó Szilvási, János Szlávik, Szabolcs Tasnády, Zsuzsanna Várnai, Péter Reményi, István Vályi-Nagy

**Affiliations:** 1 Departmental Group of Infectious Diseases, Department of Internal Medicine and Haematology, Semmelweis University, Üllői Út 26., 1085 Budapest, Hungary; 2 Department of Hematology and Stem Cell Transplantation, Central Hospital of Southern-Pest, National Institute of Hematology and Infectious Diseases, 1097, Nagyvárad Square 1., Budapest, Hungary; 3 Laboratory of Molecular Genetics, Central Hospital of Southern-Pest, National Institute of Hematology and Infectious Diseases, Budapest, Hungary; 4 Institute of Preventive Medicine and Public Health, Semmelweis University, Budapest, Hungary; 5 Department of Intensive Care Unit, Central Hospital of Southern-Pest, National Institute of Hematology and Infectious Diseases, Budapest, Hungary; 6 Department of Central Laboratory, Central Hospital of Southern-Pest, National Institute of Hematology and Infectious Diseases, Budapest, Hungary; 7 Hungarian National Blood Transfusion Service, Karolina Út 19-21., 1113 Budapest, Hungary; 8 Department of Infectious Diseases, Central Hospital of Southern-Pest, National Institute of Hematology and Infectious Diseases, Budapest, Hungary; 9 Széchenyi István University of Győr, Egyetem Square 1, 9026 Győr, Hungary; 10 Doctoral College, Semmelweis University, Budapest, Hungary

**Keywords:** COVID-19, SARS-CoV-2, Virus-specific T-cells, CD45RA^+^ depleted donor Lymphocyte infusion, Hematopoietic stem cell transplantation, Elderly, Comorbidity, Immunocompromised, Immunosenescence

## Abstract

Advanced age, comorbidities, and immunocompromised states remain major risk factors for severe or persistent COVID-19 despite vaccination and antivirals, underscoring the need for innovative treatments such as adoptive T-cell therapy (ATT). In this prospective single-center study, we evaluated the safety, feasibility, and efficacy of two ATT approaches in immunocompromised patients with high-risk or persistent SARS-CoV-2 infection: interferon-γ cytokine capture system virus-specific T cells (IFN-γ CCS VST, n = 12; median age 59) and CD45RA + T-cell depleted donor lymphocyte infusion (CD45RA^+^ TCD DLI, *n* = 11; median age 46). Most patients (73.9%) had undergone prior hematopoietic stem cell transplantation (HSCT). Both treatments were safe, with no adverse events observed. One-year overall survival (OS) did not differ significantly between groups (*p* = 0.8907 overall; *p* = 0.5907 in HSCT recipients). However, CD45RA^+^ TCD DLI showed a trend toward improved 1-year COVID-19–free survival (*p* = 0.058) and significantly better survival among HSCT recipients (*p* = 0.0362). Viral clearance was achieved in most patients (90.9% vs. 83.3%). Immunomonitoring revealed distinct immune dynamics: between weeks 5–8, IFN-γ CCS VST promoted naïve T-cell expansion with broad cytokine elevation, while CD45RA^+^ TCD DLI expanded memory T cells with a more restricted cytokine profile. IFN-γ CCS VST also elicited stronger in vivo expansion of SARS-CoV-2–specific CD4 + and CD8 + T cells. In summary, both ATT approaches are safe and effective in immunocompromised patients with persistent COVID-19. CD45RA^+^ TCD DLI, which can be generated from convalescent donors as an off-the-shelf product, may provide a practical strategy for pandemic preparedness and treatment of vulnerable patients with immune senescence.

**Graphical Abstract:**

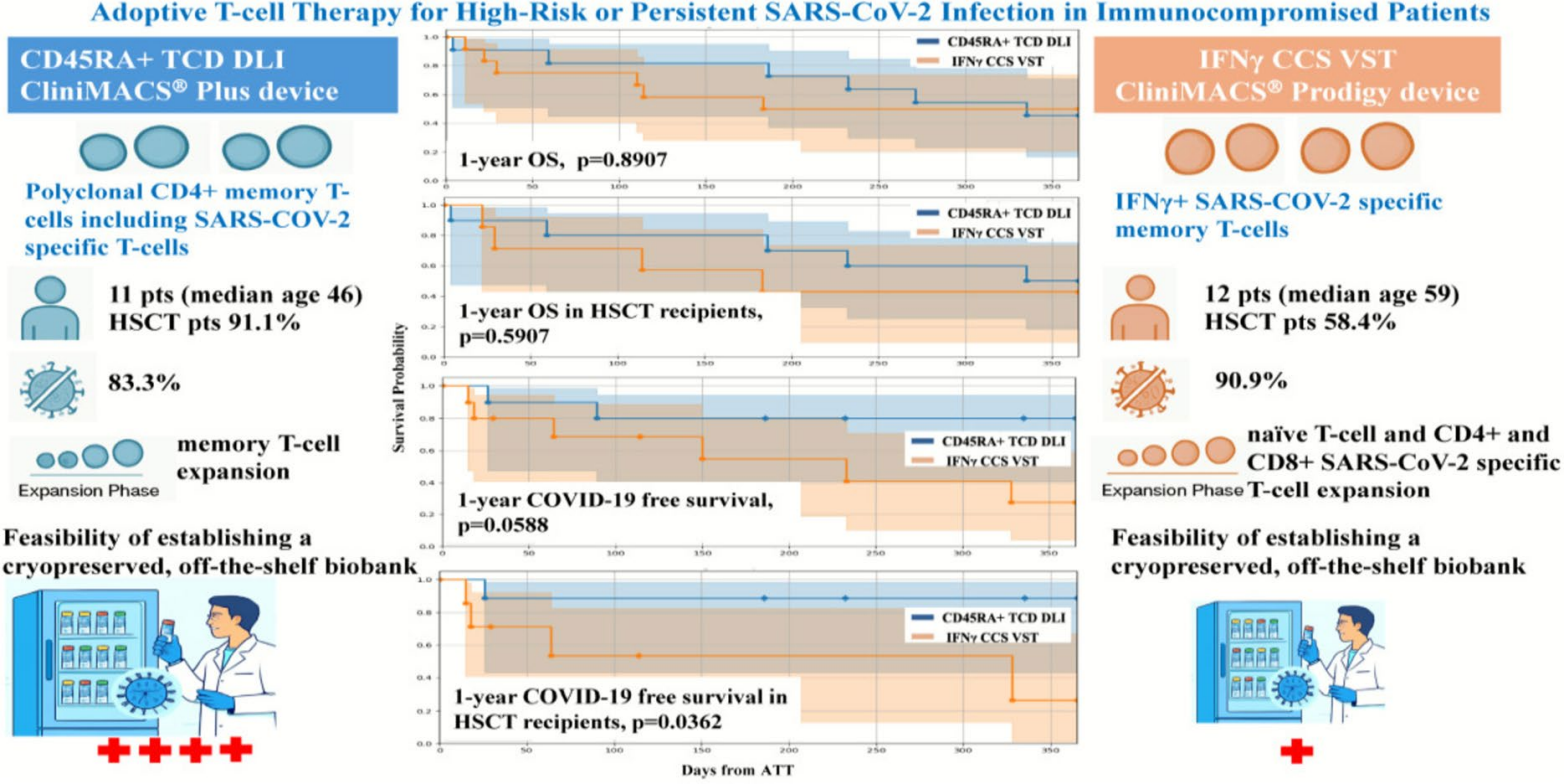

**Supplementary Information:**

The online version contains supplementary material available at 10.1007/s11357-025-02050-5.

## Introduction 

Coronavirus disease 2019 (COVID-19), caused by severe acute respiratory syndrome coronavirus 2 (SARS-CoV-2), has had an extraordinary global impact, resulting in substantial morbidity and mortality [1]. Between 2019 and 2021, global life expectancy declined by an average of 1.8 years as a direct consequence of the pandemic [2]. The burden of COVID-19 has fallen disproportionately on older adults and individuals with comorbidities, in whom frailty, sarcopenia, dementia, and immunosenescence contribute to poor clinical outcomes [3, 4]. Although by 2023–2024 the global impact of the pandemic had diminished due to widespread vaccination, high levels of population immunity, and improved diagnostics and therapies [5], SARS-CoV-2 continues to present a serious threat in vulnerable populations, particularly in the absence of robust immunity [6]. Furthermore, the morbidity and mortality associated with post-COVID-19 syndrome (long COVID), especially among older adults and males, remain ongoing concerns [7].

Patients at particularly high risk of severe or persistent COVID-19 include those who are immunocompromised, such as solid organ transplant (SOT) recipients, allogeneic hematopoietic stem cell transplant (allo-HSCT) or chimeric antigen receptor T-cell (CAR-T) therapy recipients, patients with hematologic malignancies, and individuals receiving therapies for inflammatory diseases [8, 9]. Important risk factors associated with poor vaccine response and/or worse COVID-19 outcomes include advanced age, comorbidities such as diabetes mellitus, obesity, chronic respiratory, renal or cardiovascular disease, as well as the type and number of immunosuppressive therapies [9]. In these groups, immune dysfunction whether disease-related or therapy-induced contributes to an increased risk of severe COVID-19, delayed viral clearance, higher rates of viral persistence or reactivation, and elevated mortality [10–12]. Furthermore, prolonged viral replication in immunocompromised and elderly individuals may facilitate the emergence of novel viral mutations with potential clinical and epidemiological significance [13].

Strategies aimed at reducing severe COVID-19 in immunocompromised populations include early diagnosis, close monitoring, primary vaccination regimens, pre-exposure prophylaxis with monoclonal antibodies, non-pharmacological preventive measures (e.g., masking), and rapid initiation of antiviral therapy [9]. As of 2024, the standard-of-care (SOC) for COVID-19 includes supportive care, antiviral agents such as remdesivir and nirmatrelvir/ritonavir, and immunomodulatory therapies including dexamethasone, baricitinib, or tocilizumab [14]. However, in immunocompromised and elderly patients with comorbidities, vaccine response and therapeutic efficacy of antiviral agents remain inferior compared to immunocompetent individuals, which has prompted interest in the development of various cellular-based therapeutic approaches [15–21].

Adoptive transfer of virus-specific T cells (VSTs) has shown promise in treating opportunistic viral infections in immunocompromised patients, including those after HSCT [22–29]. Our group previously reported successful clinical use of SARS-CoV-2–specific VSTs in high-risk HSCT recipients [30], and *Seng *et al. demonstrated the efficacy of COVID-19–specific VSTs generated by interferon-gamma (IFN-γ) cytokine capture system (CCS) technology **(IFN-γ CCS VST)** [31].

Another promising approach is the use of CD45RA-positive T-cell depleted donor lymphocyte infusion (CD45RA^+^ TCD DLI). By selectively removing naïve CD45RA + T cells, this strategy enriches for memory T cells with antiviral activity while reducing the risk of graft-versus-host disease (GVHD), a critical advantage for elderly or comorbid patients [32–34]. CD45RA- memory T cells encompass a repertoire against all pathogens previously encountered by the donor [32–34]. Clinical studies have confirmed the safety and efficacy of CD45RA-depleted T cells in allogeneic HSCT, with favorable outcomes including improved survival and low GVHD rates [35–37]. These memory T cells can rapidly expand in response to viral antigens, enabling clearance of otherwise refractory infections [38]. Initially, CD45RA- T cells were administered in low doses (10^4^–10^5^/kg) to promote immune reconstitution after allogeneic HSCT [39]. More recently, therapeutic dosing (≥ 10^6^/kg) has gained prominence [40].

The COVID-19 pandemic accelerated the translation of CD45RA^+^ TCD DLI into clinical practice. Inspired by the VST generation method utilizing rapid IFN-γ CCS, *Perez-Martinez and colleagues* screened donors via flow cytometry using SARS-CoV-2 peptide pools and established an off-the-shelf cryopreserved CD45RA^+^ TCD DLI bank using convalescent donors carrying common HLA alleles prevalent in the Spanish population [41, 42]. In a phase 1 dose-escalation trial, nine patients with severe COVID-19 infection characterized by lymphopenia and/or pneumonia were successfully treated with off-the-shelf CD45RA^+^ TCD DLI. These findings laid the groundwork for an ongoing phase 2 randomized prospective trial targeting elderly and/or comorbid patients with severe SARS-CoV-2 infection [43]. CD45RA^+^ TCD DLI has since been used successfully against other viral infections (e.g., cytomegalovirus (CMV), parvovirus B19) in immunocompromised patients [44–46]. At our institution, alongside the administration of SARS-CoV-2-specific VSTs generated using IFN-γ CCS, CD45RA^+^ TCD DLI therapy has also been introduced for immunocompromised patients with hematological diseases primarily post-HSCT who suffer from COVID-19 pneumonia and/or face a high risk of disease progression and/or persistent infection despite antiviral and immunomodulatory therapy. Compared to VSTs generated by ex vivo expansion, which require specialized infrastructure and prolonged manufacturing times, CD45RA^+^ TCD DLI products are easier to produce and can be used as "off-the-shelf" therapies, offering practical advantages in urgent or resource-limited settings.

Although both IFN-γ CCS VSTs and CD45RA^+^ TCD DLIs have been individually tested in severe or persistent COVID-19, a direct comparative study has not been reported. Here, we report the first prospective single-center comparison of **IFN-γ CCS VSTs** and CD45RA^+^ TCD DLI in immunocompromised patients with high-risk or persistent COVID-19, including older adults and those with hematologic malignancies. We evaluate safety, feasibility, immune responses, and clinical outcomes, and discuss the relative advantages of each approach in the context of immunosenescence and persistent viral infection.

## Materials and methods

### Study design and settings

A prospective, non-randomized, interventional study was performed between March 2021 and February 2024, among adult patients (≥ 18 years at diagnosis) with an established mainly malignant hematologic disease, receiving T-cell therapy of IFN-γ CCS VST or CD45RA^+^ TCD DLI for ongoing COVID-19 at South Pest Central Hospital, National Institute of Hematology and Infectious Diseases (SPCH-NIHID, Budapest, Hungary), a national-level tertiary-referral center. This study was conducted in accordance with national ethical standards and the principles of the Declaration of Helsinki and adhered to Good Clinical Practice guidelines. Ethical approval for the IFN-γ CCS VST or CD45RA^+^ TCD DLI studies was obtained from the Scientific and Research Ethics Committee of the Hungarian National Medical Scientific Council (IFN-γ CCS VST study Approval Number: ETT-TUKEB IV/2743–1/2021/EKU and CD45RA^+^ TCD DLI study Approval Number: ETT-TUKEB IV/8535–3/2021/EKU). Written informed consent was obtained from all participants prior to enrollment. Participants were informed about the study objectives, procedures, potential risks, benefits, and their right to withdraw at any time without penalty. Confidentiality of all personal data was ensured in compliance with national data protection laws.

### Patient selection and inclusion and exclusion criteria

All patients described above were eligible for inclusion during the study period at our center. Potential recipients were actively identified and recruited by their primary physicians and study personnel. The inclusion criteria for both T-cell therapy trials were as follows: 1) an established hematologic disease, requiring active immune-chemotherapy/immunocompromised or post HSCT status, and 2) presence of COVID-19 specific clinical symptoms, not responding to at least 1 antiviral and 1 immunomodulatory therapy targeted against SARS-CoV-2, and/or 3) presence of persistent pulmonary infiltrations on chest computed tomography (CT) scans, not attributable to other pathological processes, and/or 4) persistent SARS-CoV-2 real-time polymerase chain reaction (RT-PCR) positivity from at least 1 peripheral blood and/or oro-nasopharyngeal swab samples, taken at least 21 days apart, and 5) at least 1 human leukocyte antigen (HLA) allele match between recipient and donor, based on HLA-A, HLA-B, HLA-C, or HLA-DR antigen examination. Exclusion criteria for treatment were: 1) previous anaphylactic reaction to any blood product, or 2) under corticosteroid treatment (methylprednisolone or dexamethasone), or 3) patient unwillingness, or 4) absence of eligible donor with HLA allele match.

### Patient-level allocation of adoptive cellular therapies

Patients eligible for adoptive T-cell therapy could receive either or both modalities. At our institute, IFN-γ CCS VST became available first from April 2021, followed by CD45RA^+^ TCD DLI from June 2022. Throughout the clinical trial, we maintained cryopreserved "mini bank" pools of both IFN-γ CCS VST and CD45RA^+^ TCD DLI products to ensure timely availability. In cases requiring urgent intervention, the selection of available cryopreserved T-cell products was based on a minimum HLA allele match with the recipient.

### Data collection

An anonymized database was established for the study’s aim by manually extracting data of included patients from digital hospital records and written documentation, and transferred to a standardized case report form. Collected recipient data included: 1) age, gender, 2) comorbidities, 3) SARS-CoV-2 vaccination status, 4) underlying hematologic disease, HSCT status, such as stem cell transplantation type, time since transplantation, HLA matching, conditioning regimen, GVHD prophylaxis, presence of GVHD, 5) characteristics of COVID-19, including disease severity, presence of pneumonia, SARS-CoV-2 RNAemia, requirement of hospitalization and intensive care unit (ICU) admission, length of stay (LOS), preceding anti-COVID-19 treatments, 6) follow-up data. Baseline data were collected on the day of cellular therapy. Both adoptive T-cell treatments were followed for the appearance of complications, such as cytokine release syndrome (CRS), secondary hemophagocytic lymphohistiocytosis (sHLH), or GVHD.

### Selection of eligible donors for cellular therapy

Donors for IFN-γ CCS VST and CD45RA^+^ TCD DLI cohort were recruited through third-party, or original stem cell donor, or the volunteer donor of the Hungarian National Blood Transfusion Service (OVSZ) and selected based on their convalescent or vaccinated COVID-19 status, as well as HLA-A, -B, -C, and -DR antigen compatibility with potential recipients. All donors underwent flow cytometric analysis (FACS) to assess the presence of SARS-CoV-2-reactive T-lymphocytes [30]. Donors were considered eligible for donation if the SARS-CoV-2 specific CD4 + or CD8 + T-cell percentage was > 0.01% or close to 0.01% of all CD4 + or CD8 + T-cells, respectively [30]. Additionally, donors provided written informed consent prior to participation in the procedures.

### Leukapheresis of donors and preparation of the IFN-γ CCS VST and CD45^+^ TCD DLI product

Donor lymphocytes were collected through unstimulated leukapheresis using the Spectra Optia Apheresis System and the Continuous Mononuclear Cell Collection (CMNC) program (Terumo Blood and Cell Technologies Inc., Lakewood, USA). For the preparation of IFN-γ CCS VSTs, a total of 1 × 10^9^ white blood cells (WBCs) were processed using the CliniMACS Prodigy IFN-γ CCS (Miltenyi Biotec, Bergisch Gladbach, Germany), which selectively enriches antigen-specific, IFN-γ-secreting CD4 + and CD8 + memory T cells [30]. The required number of SARS-CoV-2-specific T cells was transfused immediately following flow cytometric analysis. In the first 3 patients treated with IFN-γ CCS VST in our previous publication [30], we gave 5 × 10^3^/kg IFN-γ producing T cells in 2 cases and 1 × 10^4^/kg IFN-γ producing T cells in the 3rd case. Subsequently, the dose used for routine IFN-γ CCS VST treatments in the remaining patients was determined by the number of alloreactive, non-IFN-γ producing T cells, which should not exceed 2.5 × 10^4^/kg, a threshold indicating the risk of developing GVHD in cell therapy practice [47, 48]. For CD45RA^+^ TCD DLIs, the CD45RA depletion was performed using the CliniMACS Plus device (Miltenyi Biotec B.V. & Co. KG, Bergisch Gladbach, Germany) according to the manufacturer's instructions. Platelets were depleted from the leukapheresis products by centrifugation. Cells were incubated with one vial of CliniMACS CD45RA Reagents (Miltenyi Biotec B.V. & Co. KG, Bergisch Gladbach, Germany) for 30 min at room temperature. Unbound reagents were washed out by centrifugation twice. CD45RA-positive cells were depleted using the CliniMACS Depletion Tubing Set (Miltenyi Biotec B.V. & Co. KG, Bergisch Gladbach, Germany) and the Depletion 3.1 program of the CliniMACS Plus device. Samples for quality control were taken from the leukapheresis products, preselection, target fraction, and non-target fraction. This process yielded a 99.8% purity of CD45RA-CD3 + T cells, a subset predominantly comprising CD45RO + and CD4 + memory T cells. The infusion dosage for COVID-19-specific CD45RA-negative memory T cells was standardized at 1 × 10^6^ CD45RA- T cells per kilogram of body weight, administered in a single session. All procedures were performed in strict accordance with the manufacturers’ protocols. For IFN-γ CCS VST products, the target fraction of non-IFNγ producing cells above 2.5 × 10^4^/kg and CD45RA^+^ TCD DLI above 1 × 10^6^ CD45RA- T-cells/kg dose were cryopreserved to generate SARS-CoV-2 specific mini bank.

### Laboratory assessments of recipients

The methods for SARS-CoV-2 RT-PCR testing from oro- and nasopharyngeal and peripheral blood samples, anti-SARS-CoV-2 serology and viral neutralization assays, as well as the measurements of multi-cytokine and chemokine levels and lymphocyte subpopulations, and detection of SARS-CoV-2 specific CD4 + and CD8 + T-cells and microchimerism have been previously described in detail in our earlier work [30, 49]. Multi-cytokine and chemokine measurements, peripheral blood FACS analysis, serological assays, and microchimerism detection were performed at screening and after adoptive T-cell therapy at weeks 1, 2, 3, and 4 and weeks 5–8. SARS-CoV-2 RT-PCR was performed weekly after T-cell administration until negative results were obtained, which corresponded to viral clearance, and routinely at months 1, 2, 6, and 12. Due to poor graft function or relapse of the underlying hematologic disease with persistent SARS-CoV-2 positivity, a CD34 + selected stem cell booster or urgent HSCT was administered. In the above cases, chimerism was then monitored using the short tandem repeats (STR) method [50].

### Patient follow-up and outcomes

Patients were followed from the day of administration of adoptive T-cell therapy until months 1, 2, 6, and 12. Patient follow-up was conducted at our dedicated COVID-19 outpatient clinic and in-patient ward (if rehospitalization occurred) of SPCH-NIHID by study personnel. Clinical follow-up included: 1) patient survival, 2) COVID-19 disease activity, including SARS-CoV-2 RT-PCR positivity in peripheral blood and oro-nasopharyngeal swabs and clinical symptoms, 3) hematologic disease activity (remission or relapse) and GVHD status (active or in remission), if applicable. The primary outcome was clinical cure, defined as a composite of: 1) cessation of COVID-19 specific symptoms (fever, cough, and dyspnea), 2) regression in laboratory parameters (serum CRP, LDH, plasma D-dimer, fibrinogen, and interleukin-6) and radiologic parameters (active COVID-19 pneumonitis on chest CT scan) with oro- and nasopharyngeal swab and peripheral blood SARS-CoV-2 RT-PCR negativity, assessed at 1 month after cellular therapy. Secondary outcomes were: 1) overall survival, 2) recurrence of clinically symptomatic COVID-19 (any severity), 3) progression or relapse of underlying malignant hematologic disease, 4) de novo or progression or relapse of GVHD (any severity), when relevant, all assessed at 12 months after cellular therapy.

### Statistical analysis

Continuous and categorical variables are expressed as medians ± interquartile ranges and numbers with percentages, respectively. Comparisons were performed by Mann–Whitney U-test or Fisher’s exact test. Kaplan–Meier analysis with log-rank testing was performed for COVID-19 free survival and total survival at 1 year between the two groups. A 2-tailed *p* < 0.05 determined the statistical significance. Statistical tests and Kaplan–Meier survival curves were generated using Python, version 3.11.13 (released June 4, 2025). An age-related subgroup analysis was performed for patients ≤ 50 and over 50 years old.

## Results

### Clinical characteristics of patients treated with SARS-CoV-2 IFN-γ CCS VST and CD45RA + TCD DLI

A total of 23 patients were included, 12 in the IFN-γ CCS VST group and 11 in the CD45RA^+^ TCD DLI group. Baseline clinical, hematological, and COVID-19 related characteristics are shown in Table 1. The median age was 53 ± 19 years, and there were no statistically significant differences between the two cohorts in terms of gender and comorbidities, although the median age was higher in the IFN-γ CCS VST group. The most common hematological malignancies were acute leukemias (47.8%) and B-cell Non-Hodgkin lymphomas (30.4%). A total of 17 (73.9%) patients underwent HSCT before SARS-CoV-2 specific adoptive T-cell therapy administration, and 13 (56.5%) allogeneic HSCT were performed. Among allogeneic transplant recipients, the proportion of haploidentical donors was higher in the CD45RA+ TCD DLI cohort; however, the difference was not statistically significant (62.5% for CD45RA^+^ TCD DLI vs. 0% for IFN-γ CCS VST, *p* = 0.09). Conditioning regimen intensity, pre-transplant disease status and preceding anti-thymocyte globulin administration, and post-transplant acute GVHD prevalence were similar between two groups. COVID-19 vaccination status was similar (63.6% for CD45RA^+^ TCD DLI vs. 75.0% for IFN-γ CCS VST, *p* = 0.67). COVID-19 pneumonitis was diagnosed in 69.6% of all patients, and it was significantly higher in the IFN-γ CCS VST group (45.5% for CD45RA^+^ TCD DLI vs. 91.7% for IFN-γ CCS VST, *p* = 0.03). During the disease course, SARS-CoV-2 RNAemia was detected in 13 (56.5% for all patients, 63,6% for CD45RA^+^ TCD DLI vs. 50% for IFN-γ CCS VST) patients. The need for hospitalization was higher in the IFN-γ CCS VST group but not statistically significant (63.6% for CD45RA^+^ TCD DLI vs. 91.7% for IFN-γ CCS VST, *p* = 0.06), and ICU admission (18.2% for CD45RA^+^ TCD DLI vs. 25% for IFN-γ CCS VST, *p* = 1.0) was similar. Hospital length of stay (LOS) median value was comparable (18 ± 24 days for CD45RA^+^ TCD DLI vs. 53 ± 50 days for IFN-γ CCS VST, *p* = 0.39) between the two groups. In the cohort, 100% of the patients received remdesivir; COVID-19 specific treatment was statistically significantly different only in regards to dexamethasone, which was more prevalent in the IFN-γ CCS VST group (9.1% for CD45RA^+^ TCD DLI vs. 66.7% for IFN-γ CCS VST, *p* = 0.01).

**Table 1:** Baseline clinical characteristics of COVID-19 infected immunocompromised recipients

Parameter	Total (*n* = 23)	CD45RA^+^TCD DLI (*n* = 11)	IFN-γ CCS VST (*n* = 12)	*p*-value
Age	53 ± 19	46 ± 14	59 ± 19	0.09
(years, median ± IQR, min‒max)	(26–78)	(26–67)	(32–78)	
Male sex (n, %)	15 (65.2%)	8(72.7%)	7(58.3%)	0.47
Comorbidities (n, %)				
- Essential hypertension	7 (30.4%)	2 (18.2%)	5 (41.7%)	0.37
- Diabetes mellitus	2 (8.7%)	0	2 (16.7%)	0.48
- Chronic respiratory disease	2(8.8%)	1 (9.1%)	1 (8.3%)	1
- Chronic renal disease	0	0	0	1
- Chronic hepatic disease	0	0	0	1
- Chronic gastrointestinal disease	2(8.7%)	1 (9.1%)	1 (8.3%)	1
Hematological disease (n, %)				
- AML	8 (34.8%)	5 (45.5%)	3 (25.0%)	0.24
- ALL	3 (13.0%)	1 (9.1%)	2 (16.7)	
- B-NHL	7 (30.4%)	1 (9.1%)	6 (50.0%)	
- T-NHL	3 (13.0%)	2 (18.2%)	1 (8.3%)	
- HL	1 (4.3%)	1 (9.1%)	0	
- SAA	1 (4.3%)	1 (9.1%)	0	
HSCT status (n, %)†				
- Allogenic	13 (56.5%)	8 (72.7%)	5 (41.7%)	0.26
- Autologous	4 (17.4%)	2 (18.2%)	2 (16.7%)	
- None	6 (26.1%)	1 (9.1%)	5 (41.7%)	
Conditioning regimen (n, %)^*^				
- MAC	11 (84.6%)	6 (75.0%)	5 (100.0%)	0.60
- RIC	2 (15.4%)	2 (25.0%)	0	
Disease status before HSCT (n,%) ^Ψ^:				
- Complete remission	12 (70.6%)	7 (70%)	5 (71.4%)	1.0
- Active disease	5 (29.4%)	3 (30.0%)	2 (28.6%)	
ATG preceding HSCT (n, %)^*^	7 (50.0%)	5 (62.5%)	2 (40.0%)	0.62
Donor type (n, %)^*^				
- MRD	2 (15.4%)	1 (12.5%)	1 (20.0%)	**0.09**
- MUD	5 (38.5%)	2 (25.0%)	3 (60.0%)	
- Haploidentical	5 (38.5%)	5 (62.5%)	0	
- Syngeneic	1 (7.6%)	0	1 (20.0%)	
Prevalence of acute GVHD (n, %)^*^	10 (71.4%)	5 (62.5%)	5 (100%)	0.23
Time to GVHD (median ± IQR, min‒max)^*^	50 ± 39 (16–117)	78 ± 55 (33–117)	38 ± 37 (16–58)	0.14
Organ affected by GVHD (n,%)^*^				
- Skin	9 (64.2%)	4 (50.0%)	5 (100.0%)	1.0
- Gut	1 (7.1%)	1 (12.5%)	0	
- Liver	1 (7.1%)	0	1 (20.0%)	
GVHD severity (n, %)^*^				
- Grade1	3 (21.4%)	2 (25.0%)	1 (20.0%)	0.43
- Grade 2	4 (28.6%)	1 (12.5%)	3 (60.0%)	
- Grade 3	3 (21.4%)	2 (25.0%)	1 (20.0%)	
- Grade 4	0	0	0	
COVID-19 vaccinated (n, %)	16 (69.6%)	7 (63.6%)	9 (75.0%)	0.67
COVID-19 pneumonitis (n, %)	16 (69.6%)	5 (45.5%)	11 (91.7%)	**0.03**
SARS-CoV-2 viraemia (n, %)^•^	13 (56.5%)	7 (63.6%)	6 (50.0%)	0.06
Requirement for hospital admission (n,%)	18 (78.3%)	7 (63.6%)	11 (91.7%)	0.16
Hospital-LOS (days, median ± IQR, min–max)	34 ± 47 (8–188)	18 ± 24 (8–94)	53 ± 50 (14–188)	0.39
Requirement for ICU admission (n, %)	5 (21.7%)	2 (18.2%)	3 (25%)	1.0
ICU-LOS (days, median ± IQR, min–max)	34 ± 40 (5–72)	39 ± 34 (5–72)	34 ± 20 (9–49)	1.0
COVID-19 specific treatment				F(.01)
- Remdesivir	23 (100%)	11 (100.0%)	12(100.0%)	1.0
- Nirmatrelvir/ritonavir	1 (4.3%)	0	1 (8.3%)	1.0
- Dexamethasone	9 (93.1%)	1 (9.1%)	8 (66.7%)	**0.01**
- Tocilizumab	1 (4.3%)	0	1 (8.3%)	1.0
- Baricitinib	2 (8.7%)	0	2 (16.7%)	0.48
- Ruxolitinib	5 (21.7%)	4 (36.4%)	1 (8.3%)	0.16
- Bamlanivimab	2 (8.7%)	0	2 (16.7%)	0.48
- Casirivimab/imdevimab	1 (4.3%)	0	1 (8.3%)	1.0
- Convalescent FFP	8 (34.8%)	2 (18.2%)	6 (50.0%)	0.19

Abbreviations: VST: virus-specific T-cells; IFN-γ CCS: interferon-γ cytokine capture system; TCD: T-cell depletion; DLI: donor memory T-cell infusion; AML: acute myeloid leukemia; ALL: acute lymphoid leukemia; B-NHL: B-cell Non-Hodgkin lymphoma; T-NHL: T-cell Non-Hodgkin lymphoma; HL: Hodgkin lymphoma; SAA: Severe aplastic anemia; HSCT: hematopoietic stem cell transplantation; MAC: myeloablative conditioning; RIC: reduced intensity conditioning; ATG: anti-thymocyte globulin; MRD: matched related donor; MUD: matched unrelated donor; GVHD: graft-versus host disease; IQR: interquartile range; LOS: length of stay; ICU: intensive care unit; FFP: fresh frozen plasma

^†^ calculated for total cohort (*n* = 23 DLI/VST = 11/12)

^*^ calculated for patients received allogenic HSCT (*n* = 13 DLI/VST = 8/5)

^**Ψ**^calculated for patients received HSCT (any type) (*n* = 17 DLI/VST = 10/7)

^•^ At any given point during the clinical course preceding cellular anti-COVID-19 therapy

*p* = 0.05 or less was considered significant difference

### Comparison of the donor characteristics and composition and doses of IFN-γ CCS VST and CD45RA^+^ TCD DLI products

Detailed parameters of donor characteristics and T-cell products are given in Table 2 and Supplementary Table 1. The median age of donors of CD45RA^+^ TCD DLI was statistically significantly higher (33.5 vs 57 years, *p* = 0.035). The gender distribution was similar between the two groups (41.7% vs 50%). All donors of IFN-γ CCS VST products were third-party, whereas for CD45RA^+^ TCD DLI, 75% were third-party and 25% were haploidentical. In both groups, the 1/6 donor-recipient HLA allele match was the most common (IFN-γ CCS VST 41.7% vs CD45RA^+^ TCD DLI 66.7%, *p* = 0.30). Donor COVID-19 status was as follows: convalescent (IFN-γ CCS VST 25% vs CD45RA^+^ TCD DLI 33.3%), convalescent and vaccinated (33.3% vs 0%), and vaccinated (41.7% vs 41.7%). During donor screening, the median number of SARS-CoV-2 specific CD4 + IFN-γ + T cells within the CD4 + T cell gate was significantly higher in the IFN-γ CCS VST group than in the CD45RA^+^ TCD DLI group (IFN-γ CCS VST median 0.034%, range 0.02–0.147% vs CD45RA^+^ TCD DLI median 0.009%, range 0.003–0.063%, *p* = 0.01). Similarly to the results of SARS-CoV-2 specific CD4 + IFN-γ + T-cell measurements, SARS-CoV-2 specific CD8 + IFN-γ + T-cells within the CD8 + T-cell gate were also significantly higher in the IFN-γ CCS VST group than in the CD45RA^+^ TCD DLI group (IFN-γ CCS VST median 0.114%, range 0.049–0.509% vs CD45RA^+^ TCD DLI median 0.009%, range 0.0–0.215%, *p* = 0.01). The CD3 + T-cell ratio was higher in the positive fraction of the end product in the VST group (57.9% vs 47%, *p* = 0.59). IFN-γ CCS VST end products were characterized by a slight CD4 + T-cell dominance, but 2 IFN-γ CCS VST products were dominated by CD8 + T-cells (median CD4 +/CD8 + T-cell ratio 1. 32). The CD45RA^+^ TCD DLI was characterized by a CD4 + T-cell dominance (median CD4 +/CD8 + T-cell ratio 10.4). The ratio of alloreactive T-cells was determined by the number of Prodigy IFN-γ CCS VST end product non-IFN-γ producing T-cells (CD4 + and CD8 + T-cells), which was median 5.42 × 10^3^/kg (range 0.81–12.76 × 10^3^/kg). In the IFN-γ CCS VST end product target fraction, the median value of SARS-CoV-2 specific IFN-γ producing T-cells was 18.63 × 10^3^/kg (range: 4.17–61.57 × 10^3^/kg). In the first 3 IFN-γ CCS VST-treated patients, we gave a dose of 5 × 10^3^/kg IFN-γ producing T-cells in 2 cases and 1 × 10^4^/kg IFN-γ producing T-cell in the 3rd case [30]. Subsequently, in the other patients, the dose used for IFN-γ CCS VST treatments routinely given for Epstein Barr virus (EBV), CMV, adenovirus (ADV), BK virus reactivations/diseases was determined by the number of alloreactive non-IFN-γ producing T-cells; the number of these cells in the T-cell preparation should not exceed 2.5 × 10^4^/kg, which is the threshold for the risk of developing GVHD in cell therapy practice [47, 51]. The proportion of alloreactive T cells in CD45RA^+^ TCD DLI produced with the CliniMACS Plus system is determined by the number of CD3 + CD45RA + T cells. The median value of CD3 +/CD45RA + alloreactive cells was 2.95 × 10^2^/kg (range: 0.0–11.9 × 10^2^/kg), which was clearly below the threshold for potential GVHD [47, 51]. Using CliniMACS Plus, the median CD3 + CD45RA- memory T-cell count was 99.97% (range: 99.88–100%), indicating coveted selection. For IFN-γ CCS VST, the median value of IFN-γ producing T-cell doses administered to patients was 17.28 × 10^3^/kg (range: 5–61.57 × 10^3^/kg). When using CD45RA^+^ TCD DLI, the median value of CD3 + CD45RA- T-cell doses administered to patients was 1.03 × 10^6^/kg (range: 0.99–1.06 × 10^6^/kg). Fresh products were administered to 75% of patients in the IFN-γ CCS VST group and 50% of those in the CD45RA^+^ TCD DLI group, while the remaining patients received a cryopreserved product from the 'mini-bank'. Three patients in the IFN-γ CCS VST group and one patient in the CD45RA^+^ TCD DLI cohort received a 2nd dose of T-cell product.

**Table 2:** Main characteristics of donors and T-cell products

Parameter (n)	Total (24)	CD45RA^+^ TCD DLI (12)	IFN-γ CCS VST (12)	*p*-value
Age (years, median ± IQR, min–max)	42 ± 25 (20–68)	57 ± 17 (20–68)	34 ± 10 (22–47)	**0.035**
Male gender (n,%)	11 (46.8)	5 (41.7)	6 (50.0)	0.68
Donor type				
- Third party	21 (87.5)	9 (75.0)	12 (100)	0.22
- Haploidentical	3 (12.5)	3 (25.0)	0	0.22
HLA-allele match (n,%)				
- 1/8	1 (4.2)	0	1 (8.3)	
- 1/6	13 (54.2)	8 (66.7)	5 (41.7)	
- 2/6	2 (8.3)	1 (8.3)	1 (8.3)	0.30
- 3/6	5 (20.8)	3 (25.0)	2 (16.7)	
- 4/6	3 (12.5)	0	3 (25.0)	
Donor COVID-19 status				
- Convalescent	7 (29.2)	4 (33.3)	3 (25.0)	
- Vaccinated	10 (41.7)	5 (41.7)	5 (41.7)	0.07
- Both	4 (16.7)	0	4 (33.3)	
- No data	3 (12.5)	3 (25.0)	0	
SARS-CoV-2 specific CD4 + IFN-γ + T-cells within CD4 + T-cell gate % median ± IQR,min–max)	0.028 ± 0.036 (0.003–0.147)	0.009 ± 0.014 (0.003–0.063)	0.034 ± 0.019 (0.02–0.147)	**0.01**
SARS-CoV-2-specific				**0.01**
CD8 + IFN-γ + T-cells within CD8 + T-cell gate (%) median ± IQR,min–max)	0.049 ± 0.141 (0.0–0.509)	0.009 ± 0.018 (0.0–0.215)	0.114 ± 0.125 (0.049–0.509)	
CD3 + T-cells (%) of T-cell products median ± IQR,min–max)	56.9 ± 25.8 (9.0–76.4)	47.0 ± 32.5 (19.0–67.0)	57.9 ± 10.9 (9.0–76.4)	0.59
median (range) or %				
1. VST IFN-γ producing cells dose (× 10^3^/kg)	NA	NA	**17.28 (5–61.57)** **f 75%** **c 25%**	
2. VST IFN-γ producing cells dose (× 10^3^/kg)	NA	NA	**5 (5–10)** **c 100%**	
1. CD3 + CD45RA-TCD dose (× 10^6^/kg)	NA	**1.03 (0.99–1.06) f 50% c 50%**	NA	
2. CD3 + CD45RA-TCD dose (× 10^6^/kg)	NA	**NA, (1 × 1.02, c)**	NA	

Abbreviation: TCD: T-cell depletion; DLI: donor lymphocyte infusion; VST: virus specific T-cell; IFN-γ CCS: interferon-γ cytokine capture system; M: male; F: female; HLA: human leukocyte antigen; IFNγ: interferon-gamma; IQR: interquartile range; PBMC: peripheral blood mononuclear cells f: fresh; c: cryopreserved; NA: not applicable

### Outcome characteristics

**Fig. 1:**
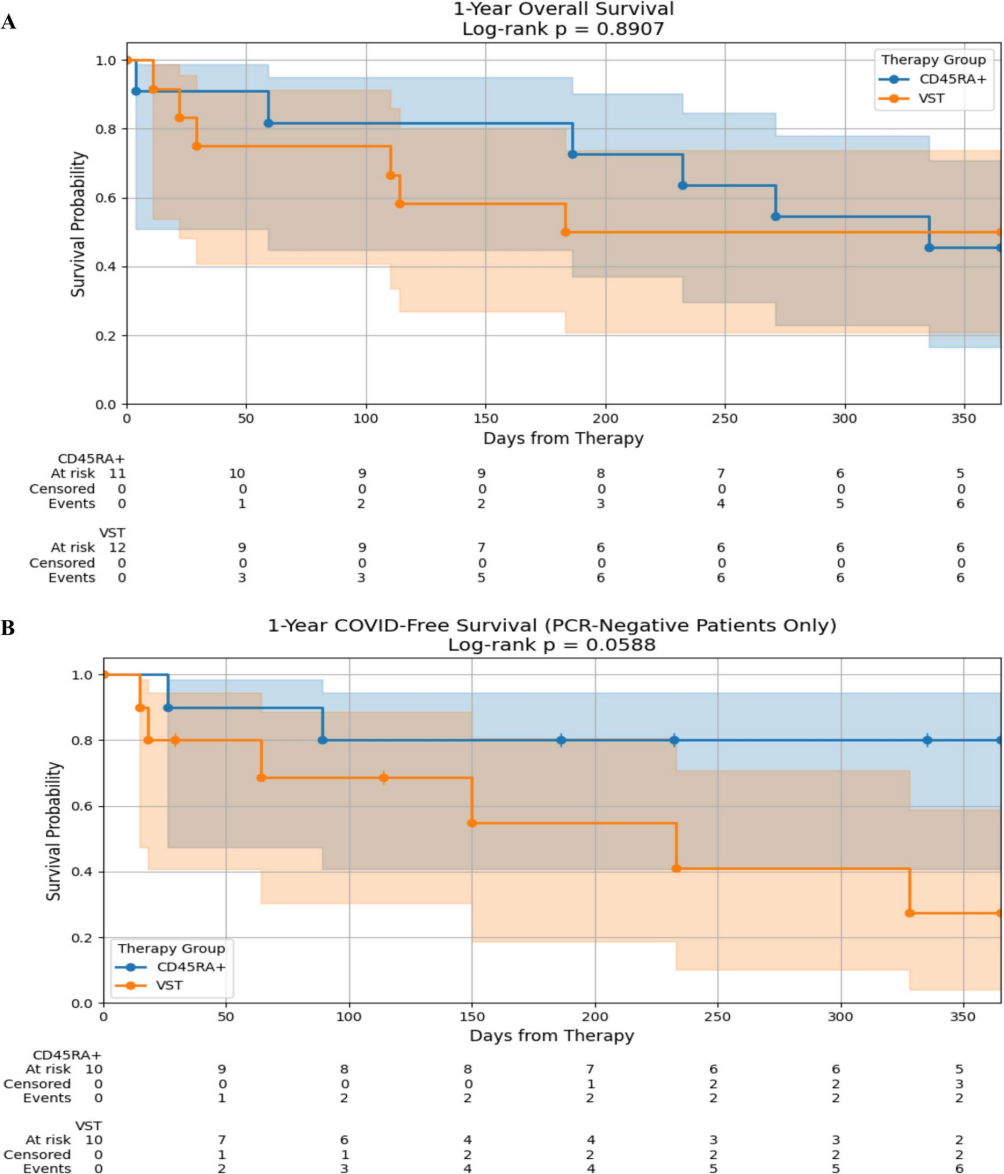

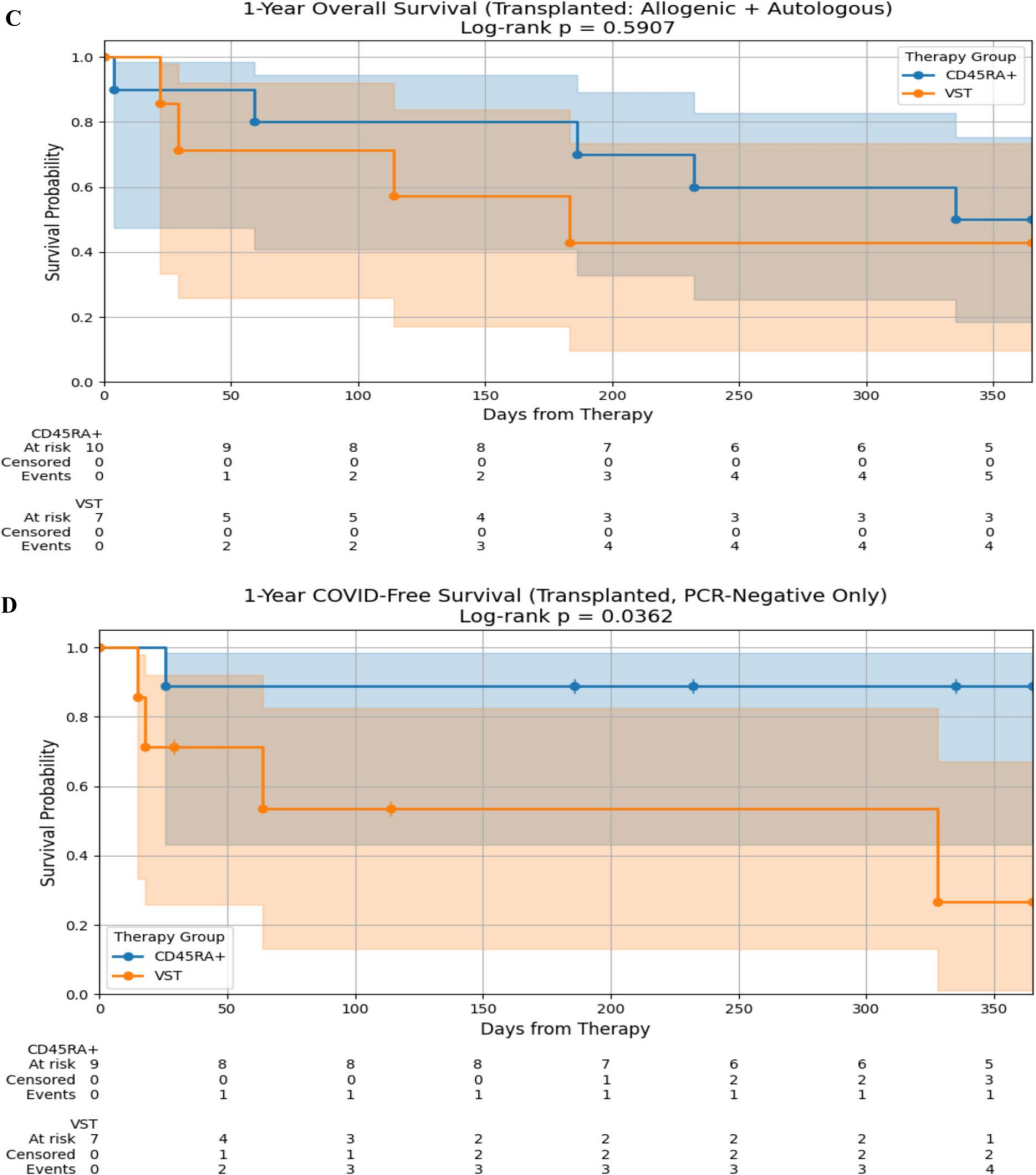
1-year Overall Survival and 1-year COVID-Free Survival. A: 1-year Overall Survival IFN-γ CCS VST vs CD45RA^+^ TCD DLI. B: 1-year COVID-Free Survival IFN-γ CCS VST vs CD45RA^+^ TCD DLI. C: 1-year Overall Survival in HSCT patients with IFN-γ CCS VST vs CD45RA^+^ TCD DLI. D: 1-year COVID-Free Survival in HSCT patients with IFN-γ CCS VST vs CD45RA^+^ TCD DLI. Abbreviations: VST: virus-specific T-cell; IFN-γ CCS: interferon-γ cytokine capture system; TCD: T-cell depleted; DLI: donor lymphocyte infusion; HSCT: hematopoietic stem cell transplantation

After ATT therapy, SARS-CoV-2 RT-PCR negativity was detected in 20 (87.0%) patients with nasopharyngeal swab specimens according to viral clearance (90.9% for CD45RA^+^ TCD DLI vs 83.3% for IFN-γ CCS VST, *p* = 1.0) (Supplementary Fig. 1). The median time to nasopharyngeal SARS-CoV-2 PCR virus clearance was the same between the 2 groups (median 3.5 weeks, range: 1–26 weeks for IFN-γ CCS VST vs median 4 weeks, range:1–27 weeks for CD45RA^+^ TCD DLI). In the CD45RA^+^ TCD DLI cohort, patient 7 of the 1 st dose of CD45RA + TCD DLI showed no viral clearance and poor graft function and therefore received a second CD34 + stem cell booster and CD45RA^+^ TCD DLI from an original haploidentical donor. Finally, after a total of 27 weeks, nasopharyngeal PCR showed viral clearance. At the administration of T-cellular therapy, each group had 1 patient with whole-blood RT-PCR positivity. At day 28, NP-PCR negativity was 54.5% in the CD45RA^+^ TCD DLI group and 50% in the IFN-γ CCS VST group. The 28-day overall survival following adoptive T-cell therapy was similar between the two cohorts (82% in the CD45RA⁺ TCD DLI group vs. 83.3% in the IFN-γ CCS VST group). Outcome parameters are shown in Table 3 and Fig. 1A-B. At 1-year, overall survival (OS) was 50% and 45.5% in the IFN-γ CCS VST and CD45RA^+^ TCD DLI cohorts, respectively (*p* = 1.0). The 1-year COVID-19 free survival was slightly higher in the SARS-CoV-2 specific CD45RA^+^ TCD DLI cohort compared to the IFN-γ CCS VST group (50% vs 20%, *p* = 0.19). Using the log-rank test, 1-year OS did not differ between the two groups (*p* = 0.8907); however, 1-year COVID-free survival was higher in the CD45RA⁺ TCD DLI cohort, although this difference did not reach statistical significance (*p* = 0.0588) (Fig. 1A-B). Among HSCT recipients, the 1-year OS was comparable between the two cohorts (*p* = 0.5907), but 1-year COVID-free survival was significantly better in the CD45RA^+^ TCD DLI group (*p* = 0.0362) (Fig. 1C-D). During the 12-month follow-up, SARS-CoV-2 reactivation was detected in 10 patients (43.5%) across the study population, most likely reflecting viral reactivation rather than reinfection. Reactivation occurred more frequently in the IFN-γ CCS VST cohort (7/12, 58.3%) compared with the CD45RA^+^ TCD DLI cohort (3/11, 27.3%). In the IFN-γ CCS VST cohort, the main predisposing factors associated with reactivation were ongoing immunochemotherapy (*n* = 3), GVHD requiring systemic immunosuppression (*n* = 2), and uncontrolled lymphoma progression without active treatment (*n* = 2). These conditions are characterized by profound impairment of both cellular and humoral immune recovery, creating an immunosuppressive milieu that may facilitate SARS-CoV-2 reactivation. In the CD45RA^+^ TCD DLI cohort, reactivation was observed in one patient with acute myeloid leukemia who underwent second and third salvage allo-HSCT with delayed immune reconstitution, and in two patients with progressive, untreated lymphoma. In these cases, the underlying disease or treatment-related immunodeficiency likely contributed to insufficient antiviral immune control. Both ATT therapies were safe; no CRS, GVHD, or sHLH were observed. Relapse or progression of hematological disease was higher in the IFN-γ CCS VST cohort (50% vs 18.2%, *p* = 0.19). The 1-year all-cause mortality was similar between the two groups (54.5% for CD45RA^+^ TCD DLI vs 50.0% for IFN-γ CCS VST, *p* = 1.0). However, the main cause of death in the IFN-γ CCS VST group was relapse or disease progression (41.7%), whereas in the CD45RA^+^ TCD DLI group, it was primarily due to transplant-related causes (45.5%).

**Table 3:** Outcome parameters of COVID-19 infected immunocompromised recipients after adoptive T-cell therapy

Parameter	Total (*n* = 23)	CD45^+^ TCD DLI (*n* = 11)	IFN-γ CCS VST (*n* = 12)	*p*-value
NP-PCR-negativity (n, %)†	20 (87.0)	10 (90.9)	10 (83.3)	1.0 (F <.01)
Whole-blood-PCR positivity at cellular therapy (n, %)^*^	2 (8.7)	1 (9.1)	1 (8.3)	0.68 (F <.01)
Whole-blood-PCR becoming negative (n,%) ¬	1 (50.0)	0	1 (100)	1.0
COVID-19 relapse (n, %)	10 (43.5)	3 (27.3)	7 (58.3)	0.21 F <.01
GVHD appearance/relapse (n, %)	2 (8.7)	0	2 (16.7)	1.0
Relapse or progression of hematological disease (n, %)	8 (34.8)	2 (18.2)	6 (50.0)	0.19
Time interval analysis (days, median ± IQR, min‒ max)				
- Time to NP-PCR negativity	32 ± 83 (1–282)	28 ± 104 (1–282)	13 ± 68 (2–226)	0.32
- Time to COVID-19 relapse	80 ± 177 (15–328)	89 ± 143 (26–312)	71 ± 151 (15–328)	N.A
28-days complete survival after adoptive T-cell therapy (%)	19 (83)	9 (82)	10 (83.3)	1.0
1-year complete survival after adoptive T-cell therapy (%)	11 (47.8)	5 (45.5)	6 (50.0)	(1.0)
28-days NP-PCR negativity (%)	12 (52.17)	6 (54.5)	6 (50)	1.0
1-year COVID-19 free survival after adoptive T-cell therapy (%)^Ψ^	7 (35.0)	5 (50.0)	2 (20.0)	(0.19)
1 year all-cause mortalityCauses of death (n, %)^°^	12 (52.2)	6 (54.5)	6 (50.0)	1.0
-Relapse/Progression	6 (26.0)	1 (9.1)	5 (41.7)	0.2
-Secondary infection	4 (17.4)	2 (18.2)	2 (16.7)	1.0
-Transplant related	7 (30.4)	5 (45.5)	2 (16.7)	0.2
-COVID-19	3 (13.0)	2 (18.2)	1 (8.3)	0.6

Abbreviations: VST: virus-specific T-cells; IFN-γ CCS: interferon-γ cytokine capture system; TCD: T-cell depletion; DLI: donor memory T-cell infusion; NP: nasopharyngeal swab; PCR: polymerase chain reaction; GVHD: graft-versus host disease; IQR: interquartile range

^†^ nasopharyngeal swab SARS-CoV-2 PCR negativity

^*^ EDTA-whole-blood SARS-CoV-2 PCR positivity

¬ calculated for patients with positive EDTA-PCR positivity at time of cellular therapy ^**Ψ**^Calculated only to patients who become SARS-CoV-2 PCR negative by nasopharyngeal swab (20, 10 vs 10) ^°^The majority of patients had multiple contributing factors to mortality, and therefore, were assigned to multiple cause-of-death categories

### Laboratory results after IFN-γ CCS VST and CD45RA + TCD DLI therapies

#### Kinetics of SARS-CoV-2 specific T-cell frequency before and after adoptive T-cell therapy

**Fig. 2:**
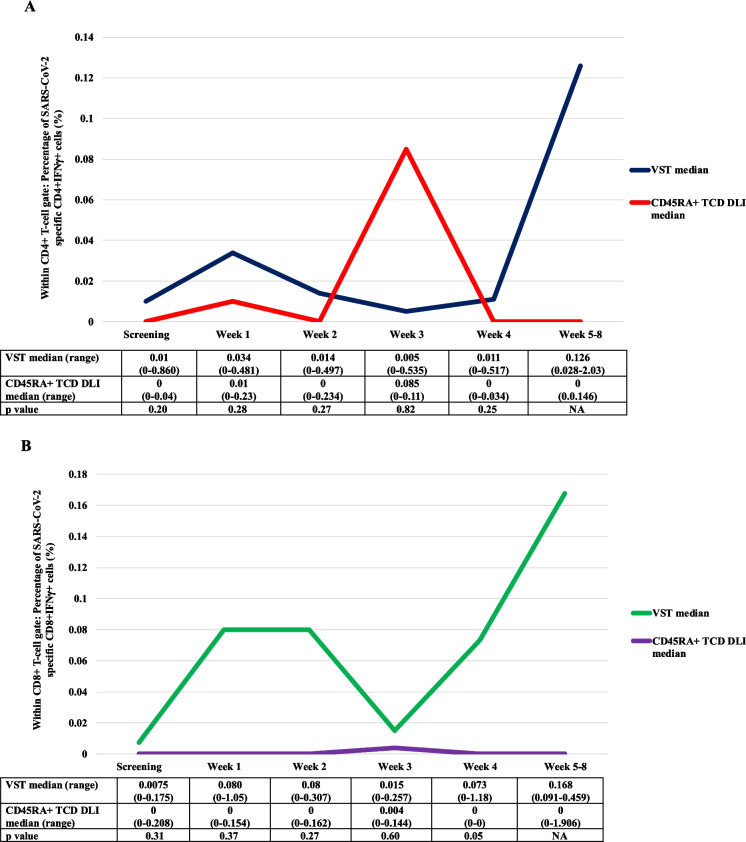

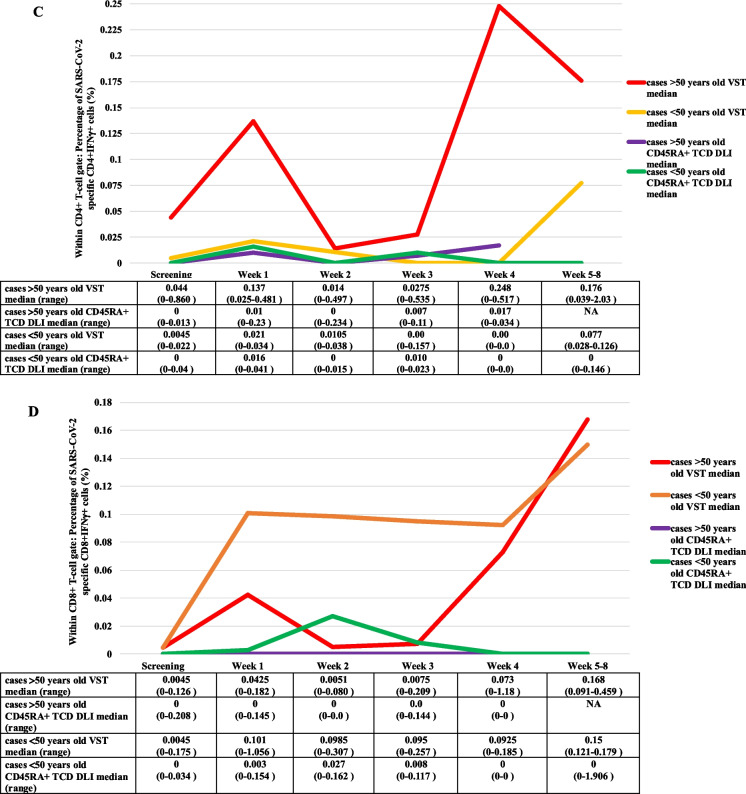
Kinetics of SARS-CoV-2 specific CD4 + and CD8 + T cells before and after adoptive T cell therapy: IFN-γ CCS VST versus CD45RA^+^ TCD DLI. **A**: Within CD4 + T-cell gate: Percentage of SARS-CoV-2 specific CD4 + IFNγ + cells (%). **B**: Within CD8 + T-cell gate: Percentage of SARS-CoV-2 specific CD8 + IFNγ + cells (%). **C**: Within CD4 + T-cell gate: Percentage of SARS-CoV-2 specific CD4 + IFNγ + cells (%) by age: over 50 years versus under 50 years. **D**: Within CD8 + T-cell gate: Percentage of SARS-CoV-2 specific CD8 + IFNγ + cells (%) by age: over 50 years versus under 50 years. Abbreviation: IFNγ: interferon-gamma; CCS: cytokine capture system; Pts: patients; VST: virus specific T cell; TCD: T-cell depleted; DLI: donor lymphocyte infusion; ND: not done; NA: not applicable

In both cohorts, the kinetics of SARS-CoV-2 specific CD4 + and CD8 + IFN-γ + T-cell frequency showed different patterns after adoptive T-cell therapy Fig. 2A–B). In all patients, at screening prior to ATT administration, the proportion of SARS-CoV-2-specific CD4 + and CD8 + IFN-γ + T-cells was virtually below the limit of detection threshold. In both cohorts, the median SARS-CoV-2 specific CD4 + IFN-γ + T-cell frequency showed an increase at week 1 (0.034% for IFN-γ CCS VST vs 0.01% for CD45RA^+^ TCD DLI, *p* = 0.28), followed by a decline by week 2. At week 3, the CD45RA⁺ TCD DLI cohort showed a clear increase in SARS-CoV-2-specific CD4 + IFN-γ + T cells (0.005% for IFN-γ CCS VST vs 0.085% for CD45RA⁺ TCD DLI, *p* = 0.82) compared to the IFN-γ CCS VST group, although the difference was not statistically significant; by week 4, levels declined below the limit of detection. The number of SARS-CoV-2 specific CD4 + IFN-γ + T-cells increased in weeks 5–8, when the detectability in the CD45RA^+^ TCD DLI cohort fell below the detection threshold, but due to the low number of cases, no statistical comparison could be made (0.126% for IFN-γ CCS VST vs 0% for CD45RA^+^ TCD DLI, *p* = NA). SARS-CoV-2 specific CD8 + IFN-γ + T cells in the IFN-γ CCS VST group showed an upward trend at weeks 1–2 (0.080% for IFN-γ CCS VST vs 0% for CD45RA^+^ TCD DLI, p = 0.37), followed by a marked decrease at week 3 and significant expansion at week 4 (0.073% for IFN-γ CCS VST vs 0% for CD45RA^+^ TCD DLI, p = 0.05). The SARS-CoV-2 specific CD8 + IFN-γ + T cells were always below the detection threshold except for week 3 in the CD45RA^+^ TCD DLI cohort.

### Comparison of changes in the characteristics of peripheral blood lymphocyte subpopulations after application of two types of adoptive T-cell therapies

The detailed parameters of changes in peripheral blood lymphocyte subpopulations following 2 adoptive T-cell therapies are shown in Supplementary Fig. 2. The CD3 + T-cell percentage at screening showed an increased percentage in IFN-γ CCS VST cohorts, followed by a significant difference in favor of the IFN-γ CCS VST group week 1 after adoptive T-cell therapy (76.87% for IFN-γ CCS VST vs 60.8% for CD45RA^+^ TCD DLI, *p* = 0.045). Between weeks 2 and 4, the groups showed no difference, with a decreasing trend in CD3 + T-cell percentage in the CD45RA^+^ TCD DLI group from weeks 5–8. to 8. The median percentage of CD8 + T-cells was elevated in the IFN-γ CCS VST group without significant difference. The proportion of CD4 + T cells was low in both groups. NK cells showed higher values in the CD45RA^+^ TCD DLI cohort without significant difference (Suppl_Fig. 2B). The proportion of CD3 + HLA-DR + activated T cells in the IFN-γ CCS VST group showed an elevated proportion throughout. There was no significant change in the proportion of regulatory T cells. In the CD4 + T-cell compartment, CD4 + CD45RO + memory T-cells behaved similarly between weeks 1 and 4, followed by an upward trend in CD45RA^+^ TCD DLI between weeks 5 and 8 (81.2% for IFN-γ CCS VST vs 96.28% for CD45RA^+^ TCD DLI, p = 0.056). Alloreactive CD4 + CD45RA + T-cells were below the normal range for the first 4 weeks, and then the naive T-cell ratio in the IFN-γ CCS VST cohort returned to the normal range between weeks 5 and 8 (Suppl_Fig. 2C). In the CD8 + T-cell compartment, CD45RA + naive T-cells showed similar proportions between weeks 1 and 4, and then significantly increased proportions in the IFN-γ CCS VST cohort between weeks 5 and 8 compared to the CD45RA^+^ TCD DLI group (70.71% vs 10.61%, *p* = 0.019). In CD8 + CD45RO + memory T cells, we observed the opposite response: similar parameters between weeks 1 and 4, followed by a significant difference between weeks 5 and 8 in favor of the CD45RA^+^ TCD DLI cohort (29.02% vs 88.34%, *p* = 0.007) (Suppl_Fig. 2D).

### Comparison of multi-cytokine and chemokine patterns following IFN-γ CCS VST versus CD45RA^+^ TCD DLI

The median values of the multi-cytokine and chemokine patterns following IFN-γ CCS VST and CD45RA^+^ TCD DLI therapy are illustrated in Supplementary Fig. 3. In both T-cell therapy groups, interferon α2 (IFN-α2), IFN-γ, interleukin-1α (IL-1α), IL-1β, IL-2, IL-4, IL-12, IL-13, IL-17A, macrophage-inflammatory protein-1α (MIP-1α), tumor necrosis factor α (TNF-α), and TNF-β all exhibited decreased or normal levels. However, statistical analysis of median values found IFN-γ to be a significant difference at week 3 in the CD45^+^ TCD DLI group (1.3 vs 6.37 pg/ml, *p* = 0.035), MIP-1α in the IFN-γ CCS VST cohort at week 2 (12. 34 vs 3.0 pg/ml, *p* = 0.042), and at weeks 3 and 4, TNF-β levels in the CD45RA^+^ TCD DLI group (1.6 vs 11.59 pg/ml, *p* = 0.004, 1.6 vs 4.69 pg/ml, *p* = 0.044) (Suppl_Fig. 3A). IL-5 was elevated between weeks 5–8 in all cohorts. IL-6 levels increased between weeks 5–8 in the IFN-γ CCS VST group (5 times the upper normal value), like IL-6 levels, although to a lesser extent (Suppl_Fig. 3B). IL-8, IL-10, and IL-15 levels also increased between weeks 5–8 in the IFN-γ CCS VST group, but statistical analysis was not possible due to the low sample size (Suppl_Fig. 3C-D). In contrast to the levels measured between weeks 5–8, IL-10 at week 4 showed significantly different levels in the CD45RA^+^ TCD DLI cohort (6.25 vs 18.39 pg/ml, *p* = 0.021) (Suppl_Fig. 3C). Interferonγ-induced protein 10 kDa (IP-10), monocyte chemoattractant protein-1 (MCP-1), and regulated upon activation, normal T-cell expressed and secreted (RANTES) showed elevated levels in both groups throughout. The IP-10/CXCL10 in the IFN-γ CCS VST group showed a downward trend in the first 2 weeks but continued to increase in both groups in weeks 5–8. MCP-1 showed variable-fluctuating behavior. RANTES levels in both groups showed a decreasing trend by weeks 5–8 (Suppl_Fig. 3D-F). Although not statistically comparable, we found a broader spectrum of cytokine and chemokine elevation patterns in the IFN-γ CCS VST group between 5–8 weeks: IL-5, IL-6, IL-8, IL-10, IL-15, MIP-1α, IP-10, IFN-γ, and MCP-1. In the CD45RA^+^ TCD DLI group, a narrower pattern of cytokine elevation was detected at weeks 5–8: IL-5, IP-10, and MCP-1.

### Assessment of microchimerism

In both the IFN-γ CCS VST and CD45RA^+^ TCD DLI cohorts, no microchimerism was detected in all but 1 case during follow-up using the digital droplet deletion-insertion (indel) PCR (ddDIP) method with a sensitivity of 0.05% (Supplementary Table 2). In most patients, we did not perform microchimerism assays on sorted T cells. In the IFN-γ CCS VST group, 10 patients were tested for microchimerism at least 2 times, but only 4 patients were tested for microchimerism on sorted T cells with negative results. In some patients, there were no informative markers for microchimerism testing between the recipient, the allogeneic-HSCT donor, and the third-party adoptive T-cell donor. Based on this, we were only able to test microchimerism in 6 patients in the CD45RA^+^ TCD DLI group. In the 3rd patient in the CD45RA^+^ TCD DLI group, we detected microchimerism between 0.754–0.209% at weeks 1 and 2 (1/6 patients, 17% detection rate).

### Subgroup analysis: comparison of IFN-γ CCS VST versus CD45RA^+^ TCD DLI results by age groups

**Fig. 3:**
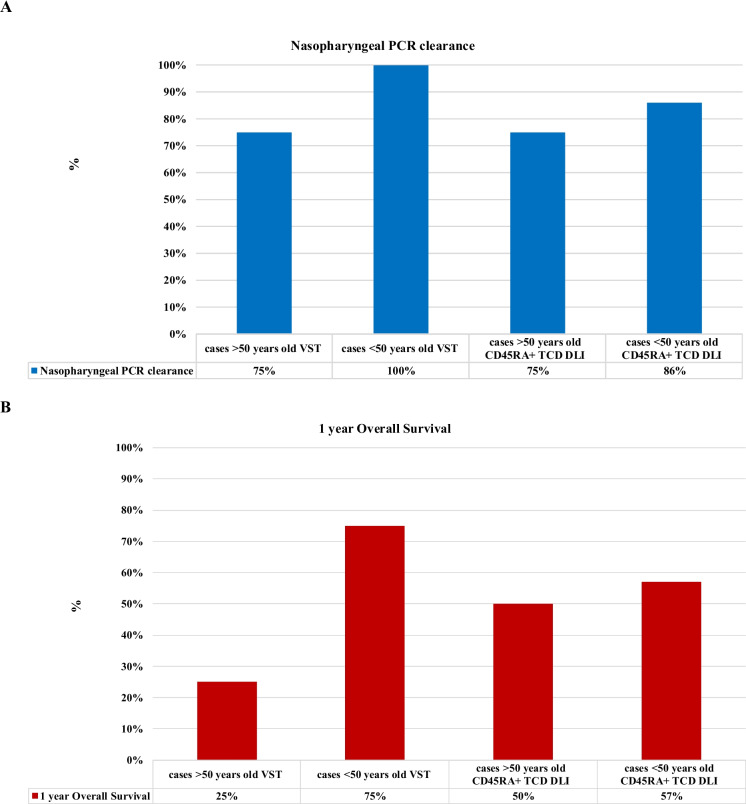
Comparison of nasopharyngeal virus clearance and 1-year overall survival in the age group over 50 years and under 50 years: IFN-γ CCS VST versus CD45RA^+^ depleted DLI. **A**: Nasopharyngeal virus clearance. **B**: 1-year overall survival. Abbreviation: VST: virus specific T cell; IFN-γ CCS: interferon-γ cytokine capture system; TCD: T-cell depletion; DLI: donor lymphocyte infusion

Both adoptive T-cell therapy groups were divided into younger subgroups under 50 years and older subgroups over 50 years to compare viral clearance, SARS-CoV-2 specific T-cell kinetics, and 1-year overall survival. Median time to nasopharyngeal PCR virus clearance showed no association with age (for IFN-γ CCS VST group age over 50 years vs under 50 years, the median 3 weeks, range: 1–17 weeks, and the median 3.5 weeks, range 1–26 weeks; for CD45RA^+^ TCD DLI group age over 50 years vs under 50 years, the median 4 weeks, range 0–27 weeks, and the median 4 weeks, range 1–27 weeks). In both groups, nasopharyngeal PCR virus clearance was higher in the group of patients under 50 years of age (for IFN-γ CCS VST group under 50 years 100% vs. over 50 years 75%, for CD45RA^+^ TCD DLI group under 50 years 86% vs. over 50 years 75%) (Fig. 3A). In the CD45RA^+^ TCD DLI cohort, patient 7 of the 1 st dose of CD45RA^+^ TCD DLI showed no viral clearance and poor graft function and therefore received a second CD34 + stem cell booster and CD45RA^+^ TCD DLI from an original haploidentical donor. Finally, after a total of 27 weeks, nasopharyngeal PCR showed viral clearance. Statistical comparisons of SARS-CoV-2 specific T cell kinetics in the subgroups by age were not possible due to the low number of cases (Fig. 2C-D). The kinetics of SARS-CoV-2 specific CD4 + IFN-γ + T-cell frequency clearly showed an increase in the IFN-γ CCS VST group over 50 years of age compared to the other 3 age subgroups. The kinetics in the IFN-γ CCS VST over 50 years of age cohort showed an increase at week 1, followed by a clear decrease at weeks 2–3 and finally a definite expansion at week 4 compared to the CD45RA^+^ TCD DLI cohort over 50 years of age. The IFN-γ CCS VST cohort under 50 years of age showed a small increase in SARS-CoV-2 specific CD4 + IFN-γ + T-cell frequency at weeks 1–2 and an increase at weeks 5–8 compared to the CD45RA^+^ TCD DLI group under 50 years of age. In both age-matched groups of the CD45RA^+^ TCD DLI cohort, we saw a small increase in the proportion of SARS-CoV-2 specific CD4 + IFN-γ + T-cells at weeks 1 and 3. However, an increase was seen in the CD45RA^+^ TCD DLI cohort over 50 years of age by week 4 compared to the under 50 years group. In conclusion, we observed improved SARS-CoV-2 CD4 + IFN-γ + T-cell induction in both cohorts of the adoptive T-cell therapy over 50 years compared to the younger cohort. The SARS-CoV-2 specific CD8 + IFN-γ + T-cell kinetics in both IFN-γ CCS VST age cohorts showed an increase compared to both CD45RA^+^ TCD DLI age cohorts. A marked SARS-CoV-2 specific CD8 + IFN-γ + T-cell induction was observed in both IFN-γ CCS VST disease age groups by weeks 4 and 5–8. In the CD45RA^+^ TCD DLI under 50 age group, we observed an increase in SARS-CoV-2 specific CD8 + IFN-γ + T cells by week 2 and a gradual decrease in the following weeks. In the CD45RA^+^ TCD DLI age group over 50 years, the SARS-CoV-2 specific CD8 + IFN-γ + T-cell rate was always very low. Overall, 1-year survival varied by age group (Fig. 3B). Over 50 years of age, 1-year overall survival was 50% in the CD45RA^+^ TCD DLI group and 25% in the IFN-γ CCS VST cohort. In contrast, below 50 years of age, 1-year overall survival was favorable in the IFN-γ CCS VST group (75% for IFN-γ CCS VST vs 57.1% for CD45RA^+^ TCD DLI).

## Discussion

### Summary of main findings

To our knowledge, this is the first prospective single-center study directly comparing IFN-γ cytokine capture system virus-specific T-cell therapy (IFN-γ CCS VST) and CD45RA-positive T-cell depleted donor lymphocyte infusion (CD45RA^+^ TCD DLI) for the treatment of persistent COVID-19 in immunocompromised patients, primarily following HSCT. Both therapies proved to be safe and feasible, with no cases of graft-versus-host disease, cytokine release syndrome, or secondary hemophagocytic lymphohistiocytosis. Clinical responses were encouraging in both groups, with improvement in symptoms, resolution of pneumonia, and high rates of nasopharyngeal viral clearance. Despite these shared benefits, several differences emerged. Relapse occurred more frequently after IFN-γ CCS VST, whereas CD45RA^+^ TCD DLI was associated with a trend toward improved one-year COVID-19–free survival overall and a significant advantage among HSCT recipients. Overall survival at one year was similar between the two groups, but age-stratified analyses revealed divergent patterns: in patients older than 50 years, survival was higher with CD45RA^+^ TCD DLI (50% vs. 25%), while in younger patients (< 50 years), outcomes slightly favored IFN-γ CCS VST (75% vs. 57%). Immune monitoring highlighted mechanistic differences between the therapies. IFN-γ CCS VST promoted in vivo expansion of naïve T-cell compartments and SARS-CoV-2–specific CD4 + and CD8 + T cells and induced broad cytokine elevations (IL-5, IL-6, IL-8, IL-10, IL-15, MIP-1α, IP-10, IFN-γ, MCP-1), particularly between weeks 5 and 8. By contrast, CD45RA^+^ TCD DLI led to predominant expansion of memory T cells and a narrower cytokine profile (IL-5, IP-10, MCP-1). Product composition also differed: IFN-γ CCS VST displayed a slight CD4 + predominance, while CD45RA^+^ TCD DLI showed clear CD4 + dominance. Together, these findings suggest that the two approaches achieve viral control through distinct immunological pathways—one favoring robust virus-specific responses, the other leveraging memory T-cell–driven immunity.

### Cellular therapies for the treatment of SARS-CoV-2 infection

New SARS-CoV-2 variants continue to disproportionately impact immunocompromised individuals and elderly/comorbid populations due to suboptimal vaccine responses and increased risk of severe disease [3, 4, 52]. Older age, multiple comorbidities (e.g., diabetes, cardiovascular and pulmonary diseases), and use of multiple immunosuppressants independently elevate severity risk [4, 8, 9]. *Ljungman *et al. compared COVID-19 outcomes in HSCT recipients, reporting 77.9% six-week survival in allogeneic and 72.1% in autologous HSCT recipients during the 2020 pandemic wave; later data showed improved survival but higher mortality in CMV-seropositive patients [53, 54]. Persistent COVID-19 infection remains without established treatment in these groups [4, 10, 55]. Potential therapies include antiviral combinations (e.g., remdesivir plus nirmatrelvir), SARS-CoV-2-specific T-cell therapies, and passive immunization with monoclonal antibodies [12, 15–17]. Prolonged infection in immunodeficient patients can lead to viral mutations with features of variants of interest, immune escape, or antiviral resistance, making them potential reservoirs for new SARS-CoV-2 variants [13, 56–58].

Three main cell therapy types are used or emerging to combat viral infections: VST, chimeric CAR-T cells, and mesenchymal stromal cells (MSCs) [59]. VSTs can be directly isolated by activation markers (CD69, CD154), peptide– major histocompatibility complex (MHC) tetramer staining, or interferon-γ capture assays, but ex vivo expansion remains the most clinically effective approach with over three decades of favorable safety data including low GVHD and infusion toxicity [60]. Multi-virus–specific expanded VST products like AlloVir’s posoleucel target several viruses (Baumann-Krech virus (BK), CMV, EBV, human herpesvirus 6 (HHV-6), John Cunningham virus (JCV), ADV), demonstrating up to 95% overall response and significant viral load reduction in phase 2 trials [27]. CAR-T cell therapies are being explored to target viral entry molecules, e.g., using mutated angiotensin-converting enzyme (ACE)–2 extracellular domains for SARS-CoV-2 targeting [61]. Additionally, NKG2D–ACE CAR-NK cells derived from umbilical cord blood are in phase 2 trials [61]. Regulatory T cell CAR therapies aim to promote tissue repair post-SARS-CoV-2 infection [62]. MSCs contribute by preventing tissue damage and supporting repair, reducing lung injury and alveolar fluid in influenza, and decreasing inflammatory damage with improved outcomes in COVID-19 patients [63, 64].

### Comparison with previous studies

#### Clinical outcomes of expanded or IFN-γ CCS VST, CD45RA^+^ TCD DLI, and other ATT approaches

Numerous publications and clinical trials have reported on adoptive T-cell therapy (ATT) for hospitalized patients with high-risk or persistent SARS-CoV-2 infection. Data on ex vivo expanded VSTs come from three reports covering 11 patients and one prospective phase 1/2 randomized trial [65–68]. Additionally, autologous ex vivo expanded VSTs have been used in a small case series [69]. Our group and *Seng *et al. have studied directly selected IFN-γ CCS VSTs, including patients post-HSCT and data from a phase 1/2 trial [30, 31]. A separate phase 1 trial evaluated third-party ex vivo expanded CD8 + T CTLs [70]. An alternative strategy involves memory T cells generated by depleting CD45RA + naïve T cells [41–43]. The randomized phase 2 RELEASE study used CD45RA^+^ TCD DLI for hospitalized SARS-CoV-2 patients [71]. Our single-center study compared two ATT approaches—IFNγ CCS VST and CD45RA^+^ TCD DLI—evaluating safety, feasibility, and laboratory outcomes.

SARS-CoV-2-specific VST generated by ex vivo expansion was evaluated in a phase 1 BATIT trial and compassionate use across 11 patients (median age 57, 91% > 50 years), mostly immunocompromised (SOT, HSCT, hematological malignancies) [65–67]. The OS after expanded VST therapy was 64%, with variable outcomes across subgroups (HSCT 75%, SOT 67%, malignancies 57%). *Papadopoulou *et al. conducted a randomized phase 1/2 trial (60 VST + SoC vs. 30 SoC during the delta variant wave) in mostly non-immunocompromised but comorbid patients (median age 57) [68]. The expanded VST arm showed higher recovery rates (65% vs. 38%, *p* = 0.017), shorter median recovery time (11 days vs. not reached), and 53% lower 60-day mortality, especially in patients > 50 years [68]. In a prospective study, autologous expanded VSTs were successfully manufactured in 10 out of 12 immunocompromised patients (83%) [69]. Three patients received autologous ex vivo expanded VSTs, resulting in viral clearance and resolution of symptoms. The efficacy of autologous expanded ATT in COVID-19 infection is provided by cytokine-induced killer T cells (CIK-T) administered alongside anti-cancer treatment. In COVID-19 patients, infection outcomes were compared across three groups: those receiving both CIK-T cells and anti-cancer therapy, those receiving anti-cancer therapy alone, and hospitalized non-cancer patients. The group treated with CIK-T cells plus anti-cancer therapy demonstrated significantly faster symptom improvement, along with reduced rates of severe infection and mortality [72]. Beyond our own group’s work, *Seng *et al. evaluated the safety and efficacy of SARS-CoV-2–specific rapid IFN-γ CCS VST in immunocompromised patients in a phase 1–2 trial [30, 31]. Like our study, 12 patients (median age 63.5, 75% hematologic malignancies) received third-party IFN-γ CCS VST (NCT04457726). Overall survival was 75%, with higher survival in HSCT recipients (83%) [31]. In our own cohorts, a higher proportion of patients—particularly allogeneic HSCT recipients—was observed (25% in *Seng *et al. vs. 9% across three expanded VST publications), indicating a higher‐risk, more vulnerable population [30, 31, 65–67]. Among the 11 high-risk patients, 3-month overall survival was 75%, with three fatalities. Age-stratified survival was 66.7% (6/9) for patients over 50 years. Tevogen Bio’s phase 1 trial (NCT04765449) administered off-the-shelf CD8 + CTLs to 12 HLA-A*02:01-positive high-risk patients (6 immunocompromised) [70]. Clinical improvement occurred within 2–3 days, with 100% 28-day survival and 91% 6-month survival; all patients > 50 years survived at 6 months. CD45RA^+^ TCD DLI has proven effective against various pathogens in refractory infections, both post-transplant and in prophylactic settings [21]. Several studies have already been completed in patients with severe lymphopenia and/or post-HSCT [73, 74]. A multicenter phase 2 trial (NCT04578210) comparing 1 × 10⁶/kg CD45RA^+^ TCD DLI + SoC versus SoC alone in non-immunocompromised patients but based on age and comorbidity profiles, most patients in both arms were high-risk for COVID-19 progression [71]. At day 14, 74% of patients in the CD45RA^+^ TCD DLI + SoC arm had recovered from COVID-19 vs 47.5% in the SoC arm (*p* = 0.03) and higher lymphocyte counts, though no mortality or hospital stay benefit. The trial supports CD45RA^+^ TCD DLI’s potential for immunocompromised patients. Our data confirm these findings, showing 28-day OS of 82% (CD45RA^+^ TCD DLI) and 83.3% (IFN-γ CCS VST), consistent with published studies. No significant difference in 1-year OS was observed (*p* = 0.89), but 1-year COVID-19–free survival tended to favor CD45RA^+^ TCD DLI (*p* = 0.059), reaching significance in HSCT recipients (*p* = 0.036).

### Timing of ATT administration

The enrolled patients presented with persistent COVID-19 unresponsive to conventional SARS-CoV-2–specific antiviral therapies. Due to ongoing viral replication, oncologic treatments frequently had to be postponed, adversely affecting long-term survival, particularly among older or frail patients with limited physiological reserve. Therefore, earlier intervention with ATT may promote infection resolution and mitigate the cumulative negative effects of both COVID-19 and the underlying disease. An additional key factor influencing ATT efficacy is the timing of therapy relative to infection onset and the time required for T-cell product preparation. At the beginning of our study, donor screening, SARS-CoV-2–specific T-cell identification, and HLA matching were time-consuming processes. Subsequently, with the availability of cryopreserved SARS-CoV-2–specific T-cell products, patients could receive therapy much more rapidly. A similar trend was observed in three reports of expanded VST therapy involving 11 patients [65–67]. The interval between COVID-19 infection and VST administration significantly decreased with the use of cryopreserved products: for the first seven patients, the median interval was 34 days (range 15–245 days), whereas for the last four patients, it was only 5–14 days. Early administration of expanded VST (within 30 days of infection) resulted in superior survival compared to delayed treatment (> 30 days) (6/7 patients, 86% vs. 1/4 patients, 25%) [65–67]. In another study, expanded third-party CD8⁺ CTLs were administered within 96 h of COVID-19 diagnosis, yielding excellent outcomes [70]. In two randomized trials, ATT was initiated within 7 days of symptom onset for expanded VST and at a median of 9 days for CD45RA + TCD DLI [68, 71]. Expanded VST therapy resulted in a 60-day mortality reduction, and CD45RA^+^ TCD DLI demonstrated significantly improved viral clearance at 14 days compared to SoC. Overall, the available evidence clearly indicates that early identification of high-risk patients and prompt initiation of ATT improves clinical outcomes. However, achieving this requires the establishment of cryopreserved cell banks and well-coordinated multidisciplinary clinical networks.

### Impact of baseline immunosuppression and prior antiviral therapy on ATT efficacy

Our findings highlight that background immunosuppression substantially modulates both the clinical and immunological efficacy of ATT. While IFN-γ CCS VSTs are highly sensitive to corticosteroids and calcineurin inhibitors, CD45RA^+^ TCD DLI products demonstrate relative resistance, allowing their administration even under moderate steroid exposure [21]. Despite this advantage, baseline immunosuppression continues to delay immune reconstitution in HSCT and SOT recipients. Current experimental strategies such as steroid- or tacrolimus-resistant cellular engineering remain technically demanding, leaving CD45RA^+^ TCD DLI the most feasible option for broader clinical use [30].

In persistent SARS-CoV-2 infection, concomitant antiviral and immunomodulatory treatments reduce proinflammatory cytokines and support lymphocyte recovery, yet fail to achieve full immune restoration in many immunocompromised hosts. In these settings, the addition of ATT to standard of care has been associated with accelerated lymphocyte subset normalization and cytokine modulation, suggesting that T-cell–based immunotherapy directly contributes to viral clearance and immunological recovery [68, 71].

### Viral clearance

Among 11 patients receiving expanded VSTs, 7 achieved sustained viral clearance while 4 showed transient responses, all demonstrating viral load reduction [65–67]. In a randomized phase 1/2 trial compared to standard of care (SoC), ex vivo expanded VST treatment led to significantly faster viral clearance (*p* = 0.017), with 30% versus 20% of patients SARS-CoV-2 negative by day 10 [68]. Autologous expanded VST recipients cleared the virus between 2- and 8-weeks post-infusion [69]. Expanded CD8 + CTL therapy induced rapid responses, with > 88% and > 99% viral clearance in NP swabs at days 4 and 14, respectively; 25% were PCR-negative by day 4 and 83% by day 14 [70]. In the phase 1–2 IFN-γ CCS VST trial, the median time to NP PCR negativity was 12 days (range 5–154) [31]. The phase 2 CD45RA^+^ TCD DLI study showed no significant difference in PCR negativity compared to SoC at days 7 (27% vs. 33%) or 14 (50% vs. 56%) [71]. Our data showed 28-day PCR negativity rates of 54.5% (CD45RA⁺ TCD DLI) and 50% (IFN-γ CCS VST). Viral clearance varied by age: patients > 50 years reached 75% clearance in both groups, while those < 50 years had 100% clearance with IFN-γ CCS VST and 86% with CD45RA⁺ TCD DLI. Despite differing assessment times across studies limiting direct comparison, ATT clearly facilitated SARS-CoV-2 elimination.

### Safety

Among the 11 patients who received expanded VSTs, two cases of grade 3 CRS were reported; one was caused by COVID-19 infection, and in the other case, a skin rash developed and was considered a possible CRS [66, 67]. In the phase 1–2 studies involving expanded VSTs, CD8⁺ CTLs, IFN-γ CCS VSTs, CD45RA^+^ TCD DLI, as well as in our own IFN-γ CCS VST and CD45RA⁺ TCD DLI cohorts, no DLTs, CRS, or GVHD were observed [30, 31, 65–71]. Based on these findings, it can be concluded that various SARS-CoV-2-specific ATT approaches are safe and well tolerated, even in high-risk patient populations, with minimal adverse effects.

### Mechanistic insights

#### Distinct immune response profiles

*Papadopoulou *et al. showed significantly improved CD3 + T-cell reconstitution (*p* = 0.0024) and higher NK cell counts in the expanded VST arm [68]. In the RELEASE study, day 7 analysis revealed CD8 + CD45RA + naïve T cells at 50% in the CD45RA⁺ TCD DLI plus SoC group versus 37% in SoC alone, while memory CD8 + CD45RO + T cells were 49% versus 62%, respectively, like proportions observed in our CD45RA^+^ TCD DLI cohort at one week [71]. In our study, T-cell percentages in the CD45RA^+^ TCD DLI group rose until week 4, then declined during weeks 5–8; NK cells increased steadily except for a transient dip at week 4. IFN-γ CCS VST treatment did not significantly alter T or NK cell proportions. In contrast, Seng et al. demonstrated increases in CD3⁺ and CD4/CD8 T-cell counts at weeks 1 and 2 [31]. Notably, between weeks 5 and 8, patients receiving IFN-γ CCS VST exhibited expansion of naïve CD4 + and CD8 + T cells, as first reported in our initial cohort and confirmed in the full IFN-γ CCS VST group [30]. This is the first report documenting late-phase naïve T-cell expansion post IFN-γ CCS VST infusion. Conversely, memory T-cell expansion following CD45RA⁺ TCD DLI aligns with prior studies [34, 73]. We observed clear evidence of B-cell regeneration between weeks 5 and 8 following both ATT modalities. B-cell reconstitution occurred among long-term survivors in both treatment arms; however, we cannot conclusively attribute B-cell recovery to ATT.

### Multi-cytokine profiles

Cytokine and chemokine profiles following ATT have not previously been directly compared or monitored over as long a timeframe as in our study. Unlike the RELEASE study, we did not observe an early decline in IP-10/CXCL10 in the CD45RA⁺ TCD DLI group, whereas the IFN-γ CCS VST cohort showed the expected decrease during the first two weeks [30, 71]. Both groups demonstrated a novel rebound of IP-10 between weeks 5 and 8. Elevated CXCL10 is linked to severe COVID-19 cytokine release syndrome and typically normalizes with clinical recovery [49]. In ex vivo expanded SARS-CoV-2 VST trials, Th1 cytokines predominate post-treatment; however, apart from a slight IFN-γ increase within normal limits at week 1 in our IFN-γ CCS VST group, we found no significant changes in IL-2, TNF-α, or TNF-β [68]. Between weeks 5 and 8, the IFN-γ CCS VST cohort exhibited elevated IL-5, IL-6, IL-8, IL-10, IL-15, MIP-1α, IP-10, IFN-γ, and MCP-1, while the CD45RA⁺ TCD DLI group showed more limited increases in IL-5, IP-10, and MCP-1.

### Expansion and persistence of ATT

Following SARS-CoV-2 ATT infusion, in vivo persistence and expansion of infused cells were evaluated using various methods. In a phase 1–2 trial of ex vivo expanded VSTs, sorted T cells were analyzed by NGS with 0.1% sensitivity [68]. Microchimerism analysis detected a median of 1.72% donor-derived VST microchimerism on day 5, with a peak of 2.63% on day 15; donor microchimerism persisted for at least 60 days. IFNγ CCS VST persistence was monitored up to day 28 by STR (1% sensitivity) and RT-qPCR of sorted T cells (0.001–0.05% sensitivity) [31]. Donor microchimerism was detected in 73% of patients; all with microchimerism recovered, while some without it died. *Ferreras *et al. reported microchimerism in sorted T cells, detectable in 64.3% of patients at week 3 and 47.6% at week 4 using RT-qPCR (0.01–0.05% sensitivity) [71]. *Lee *et al. used flow cytometric detection of SARS-CoV-2-specific pentamer-positive T cells to track infused autologous VSTs up to 6 months post-infusion [69]. In our study, microchimerism detection was limited: in the IFNγ CCS VST group (10 patients via peripheral blood mononuclear cells (PBMC); 4 via sorted T cells), no microchimerism was found. In the CD45RA^+^ TCD DLI group, infused T-cell persistence was demonstrated in 16% of patients during weeks 1–2 using the DIP method (performed on 6 patients). A major challenge for microchimerism detection was that 58.7% of patients had undergone allogeneic HSCT, limiting available DIP-informative markers to distinguish recipient, stem cell donor, and third-party ATT donor, rendering microchimerism assessment often unfeasible.

### Dynamics of virus-specific T cells after ATT

After ATT, virus-specific T cells in vivo may derive from donor or recipient origin. Various methods have been used to detect and quantify these cells, including cytokine release assays (CRA), IFN-γ Enzyme-Linked Immunospot (ELISpot), and peptide-stimulated flow cytometry measuring IFN-γ + cells within CD4 + and CD8 + T-cell gates [30, 31, 68]. *Seng *et al. reported SARS-CoV-2-specific responses by CRA in 5 patients, detectable pre-ATT in four and post-ATT in one [31]. *Papadopoulou *et al. showed that IFN-γ ELISpot levels of SARS-CoV-2-specific T cells remained significantly elevated up to 22 days post-infusion, correlating with clinical recovery, but differences diminished and were no longer statistically significant (p = 0.183) by day 60 [68]. The most precise method is peptide pool stimulation followed by flow cytometric measurement of IFN-γ + CD4 + and CD8 + T cells, which we used to track SARS-CoV-2-specific T-cell dynamics after both ATT types [30]. This method quantifies CD4 + and CD8 + subsets separately but cannot distinguish donor versus recipient origin. In our study, after CD45RA^+^ TCD DLI, CD4 + IFN-γ + T cells increased rapidly but declined sharply; IFN-γ CCS VST cohort induced a slower, sustained CD4 + IFN-γ + T-cell expansion lasting through weeks 5–8. Despite the lower frequency of SARS-CoV-2-specific T cells in CD45RA^+^ TCD DLI, efficacy remained adequate, likely due to the predominance of CD4 + memory T cells, which induced a rapid but transient response. For CD8 + IFN-γ + cells, IFN-γ CCS VST recipients showed an initial rise, a dip at week 3, then robust expansion between weeks 4 and 8. CD45RA^+^ TCD DLI recipients had minimal SARS-CoV-2-specific CD8 + IFN-γ + T-cell responses except at week 3, despite broad polyclonal memory CD8 + T-cell expansion. Persistence of expanded VSTs has been definitively demonstrated using deep TCRβ complementarity determining region 3 (CDR3) sequencing [75]. *Grosso *et al. identified endogenous T-cell responses by novel TCRβ clones post-infusion and observed a 6.6-fold higher expansion of donor clones compared to endogenous ones, persisting up to 6 months [70]. Similarly, *Vasileiou *et al. showed durable SARS-CoV-2-reactive T-cell persistence beyond six months using TCRβ sequencing [66].

### Role of HLA matching

Contrary to the findings reported by *Haidar *et al., which suggested that clinical response did not depend on HLA matching, an analysis combining 11 patients from three publications into one cohort yielded opposite results [65–67]. In cases of low HLA matching (1–3 out of 8 HLA alleles matched), survival was 43% (3/7 patients), compared to 100% survival (4/4 patients) in cases of high HLA matching (4–8 out of 8 alleles matched). These may represent the first data suggesting the importance of better HLA matching in COVID-19 VST therapy. *Papadopoulou *et al. required at least one shared HLA-DRB1 allele for eligibility; their CD4⁺-dominant expanded VST products highlighted the significance of MHC class II matching, though this did not affect microchimerism or overall survival [68]. *Grosso *et al. used ex vivo expanded CD8⁺ CTLs matched at HLA-A02:01, resulting in rapid clinical improvement [70]. The IFN-γ CCS VST trial mandated at least one shared HLA allele; most donor-recipient pairs matched at two or more loci [31]. The CD45RA^+^ TCD DLI phase 2 study found associations between specific HLA alleles (HLA-A02:01, DPB104:01) and early lymphocyte recovery [71]. In our cohorts, the most common HLA match was 1 of 6 alleles. Overall, there is no statistically confirmed link in the literature between HLA matching and T-cell persistence or survival. SARS-CoV-2-specific adoptive T-cell therapies demonstrate that both CD4 + and CD8 + subsets contribute critically to viral clearance.

### Aging and immunosenescence in persistent COVID-19 infection and their therapeutic implications

Further examination of the patient population at high risk for and particularly affected by persistent COVID-19 infection reveals common underlying pathophysiological characteristics. In elderly and/or comorbid and immunocompromised patients, pre-existing senescent and immunosenescence-related phenomena may be mutually triggered or exacerbated during SARS-CoV-2 infection [4, 9, 76, 77]. The first description of cellular senescence was provided by *Leonard Hayflick* in the 1960 s [78, 79]. Senescent cells are aged, non-functional cells with impaired metabolism that are resistant to programmed cell death. In young organisms, senescent cells are cleared by the innate immune system, but the role of the adaptive immune system in this process is less well understood [79]. Senescent cells undergo metabolic changes, apoptosis resistance, and develop a senescence-associated secretory phenotype (SASP). The SASP-mediated infectious senescence propagates and amplifies chronic inflammation causing tissue damage and promoting the development of age-related diseases [76, 77]. Another characteristic of senescent cells is their unique antigen signature distinct from healthy cells, due to genomic instability leading to the activation of endogenous retroelements and latent viruses (e.g., CMV) [76, 80]. Moreover, COVID-19 infection triggers senescence, referred to as virus-induced senescence (VIS), which along with SASP further amplifies the senescence phenomenon, facilitating the occurrence of persistent COVID-19 infection [77, 80].

Immunosenescence is the gradual decline of the immune system with age, which is associated with increased susceptibility to infections, reduced vaccine responses, and a higher risk of tumor development [81, 82]. The above process affects both the innate and adaptive immune systems. The immunopeptidome of senescent cells is highly immunogenic, making it suitable for the development of personalized therapeutic approaches [79]. Immunosenescence markers include increased proportions of CD57 +, PD-1 +, TIM-3 + senescent T cells, a decreased naïve T cell pool, and an inflammatory mediator profile creating a strongly tolerogenic and inflammatory environment that impedes viral clearance [83]. Aging-related immunosenescence results in decreased T regulatory cell activity, exaggerated Th17 responses, and persistent low-grade inflammation [84].

Senolytic agents target the BCL-2, p53, and PI3K/AKT pathways, which are crucial for the survival of senescent cells [85]. Furthermore, current research is exploring various senolytic drugs, including dasatinib, quercetin, fisetin, navitoclax, and UBX0101, as well as immunotherapeutic approaches such as the CD153 vaccine, CDX-011 (glembatumumab vedotin), and T cell- or NK cell–based senotherapies aimed at eliminating senescent cells. T cells serve as the primary effectors in senescent-cell surveillance and thus represent promising targets for senolytic immunotherapy [77, 86]. Potential approaches include CAR-T or TCR-engineered T-cell therapies directed against urokinase-type plasminogen activator receptor (uPAR) and NKG2D ligands expressed on senescent cells [76, 87]. The uPAR CAR T cells safely eliminated senescent cells in a mouse model [88]. The NKG2D-CAR T cells eliminated senescent cells in aged mice and nonhuman primates [89]. In a mouse model, fibroblast activation protein (FAP) CAR-CD8 + T cells significantly reduced cardiac fibrosis and restored impaired function [90]. Moreover, senolytic CAR-T-cell treatment may be combined with small-molecule senolytics such as dasatinib + quercetin or with PD-1 checkpoint inhibitors [91]. Additional strategies under investigation include low-dose interleukin-2 administration to expand autologous regulatory T cells or infusion of allogeneic Tregs derived from umbilical cord blood [82, 92, 93]. Another potential approach based on strong immunogenicity is the use of senescent cell-derived nanovesicles [79]. Natural killer (NK) cells play a critical role in the immune system; however, immunosenescence associated with aging reduces the ability to combat infections, clear senescent cells, and inhibit tumor development. To date, mainly autologous expanded NK cells have been used to eliminate immunosenescent cells [77, 94]. Two infusions of expanded autologous NK cells improved the immunosenescence phenotype for over one year in human PBMCs [86, 95]. Finally, NK cell infusions from allogeneic healthy donors have shown rejuvenating effects on immunosenescence [94].

The COVID-19 pandemic highlighted a stronger impact on the human population than other respiratory viruses, e.g., long COVID development, and appears to accelerate aging through nuclear deoxyribonucleic acid (DNA) damage [96]. It has been demonstrated that severe COVID-19 infection significantly accelerates immune aging and vascular aging compared to control groups [83, 97]. Furthermore, in one study, even after mild or asymptomatic COVID-19 infection, accelerated biological aging was demonstrated in induced sputum cells through the assessment of DNA methylation age (DNAmAge) and telomere length [98].

Similar senescence/immunosenescence phenomena are observed in immunocompromised populations. A comparison between allogeneic HSCT recipients and healthy controls showed a higher proportion of CD57 + senescent cells post-HSCT: 23% vs. 58% [99]. Increased senescent T cell frequencies correlate with more frequent infections, reduced vaccine responses, poorer graft-versus-leukemia effects, and higher risks of late complications. In animal models (mice and hamsters), age-related accumulation of senescent cells is associated with higher SARS-CoV-2 viral loads and decreased virus clearance in older animals [100]. In severe COVID-19 survivors, elevated proportions of CD28-CD57 + senescent CD4 +/CD8 + T cells persist 6 months post-infection, with decreased naïve T cells and increased memory T cell compartments [101]. Immunosenescence, senescent cell accumulation, and SASP propagation collectively may cause persistent COVID-19 infection by impairing effective viral clearance. Collectively, senescence, immunosenescence, and SASP disrupt effective viral clearance and promote persistent SARS-CoV-2 infection, especially in immunocompromised hosts [102]. Therefore, it is understandable that the risk groups for COVID-19 infection, as confirmed by multiple studies [8], are linked through the development and exacerbation of senescence/immunosenescence, combined with SARS-CoV-2-induced VIS and SASP spread. Hence, the adoptive transfer of allogeneic T cells from healthy donors represents an effective therapeutic approach for treating patients with senescent cell accumulation, immune dysfunction, and exhaustion who are suffering from persistent SARS-CoV-2 infection, aiming to achieve viral clearance. Until NK cell–, T cell–, and CAR-T cell–based senotherapeutic methods become widely available for reversing senescent accumulation, the use of off-the-shelf ATT products may provide a viable solution for immunocompromised patients to combat severe or persistent infections. In the future, these efforts should integrate pharmacological and cellular-based senotherapies with ATT approaches to overcome virus-induced senescence (VIS) and persistent SARS-CoV-2 infection.

### Emerging perspectives for the clinical application of ATT

In IFN-γ CCS VST therapy, the antiviral effect is primarily mediated by IFNγ–producing T cells that recognize viral antigens. However, these products may also contain non–IFNγ–producing alloreactive T cells. As noted in previous reports, the proportion of alloreactive T cells should remain below the GVHD activation threshold, yet the potential immunological role of these alloreactive subsets remains poorly understood [47, 48]. In contrast, CD45RA^+^ TCD DLI products contain the entire CD45RA- memory T-cell compartment, predominantly CD4 + subsets, encompassing the donor’s lifetime-acquired memory repertoire against multiple pathogens [41]. Consequently, CD45RA^+^ TCD DLI may provide broader immune support in frail and immunosenescent patients, potentially facilitating recovery from both SARS-CoV-2 infection and secondary opportunistic infections. Furthermore, because CD45RA^+^ TCD DLI includes tumor-specific memory T cells, its theoretical antitumor potential has been proposed. Indeed, several studies in hematologic settings have reported beneficial effects in relapse or mixed chimerism, although results from pediatric cohorts have been less conclusive [33]. In our own experience, CD45RA^+^ TCD DLI has been safely administered for relapse treatment; however, given the limited patient numbers, further studies are warranted to clarify its role in this indication.

### Translational and practical considerations

The ATT initially targeted refractory viral infections occurring in immunocompromised patients following allogeneic HSCT for hematologic malignancies, as well as prophylactically aimed at improving immune reconstitution [22–28, 39, 73, 74]. However, observing the safety and success of ATT, its use expanded to solid organ transplant (SOT) recipients and patients with primary immunodeficiencies [29, 60]. A further paradigm shift in the application concept occurred with the emergence of the COVID-19 pandemic, which revealed a significantly larger vulnerable patient population within the human population, including the elderly and those with comorbidities. During the administration of SARS-CoV-2-specific ATT, multiple clinical trials have demonstrated its suitability, safety, and efficacy even in these vulnerable patient groups [31, 68, 71]. Among SARS-CoV-2-specific ATTs, expanded VST and CD8 + CTL show clear clinical efficacy with high cell doses, rapid responses, and prolonged persistence [65–68, 70]. However, their production is complex, costly, requires genetically modified organism (GMO) accreditation, and is classified as Advanced Therapy Medicinal Products (ATMP), limiting off-the-shelf biobank availability for broad high-risk population use [71]. The efficacy of autologous expanded VST, especially in elderly and comorbid patients, remains uncertain. Immunocompromised patients face challenges like lymphopenia and T-cell exhaustion, similar to CAR-T manufacturing.

The ATTs used in our study require specialized expertise and advanced laboratory infrastructure; however, neither qualifies as an ATMP, and thus their manufacturing is not subject to complex legal regulation [71]. IFNγ CCS VST are produced using the CliniMACS Prodigy device, whereas CD45RA^+^ TCD DLI are prepared using the CliniMACS Plus system. IFN-γ CCS VST is more expensive (~ 17,400 euro (EUR)/20,500 United States dollar (USD)) than CD45RA⁺ TCD DLI (~ 10,000 EUR/11,700 USD). Both approaches have the potential for biobank-based, off-the-shelf product development. However, their clinical applications differ significantly. In most clinical trials and small case series evaluating SARS-CoV-2 ATT, convalescent donors have been used. Exceptions include the study by *Seng *et al. [31] and our own institutional clinical study, in which donors who had only undergone vaccination were also utilized. Currently, no clinical study or publication is available that directly compares the efficacy of ATT derived from convalescent versus vaccinated donors. Based on our own findings, the frequency of SARS-CoV-2–specific T cells measured during donor screening was significantly lower in vaccinated donors compared to convalescent donors. Moreover, it is well established that vaccinated donors mount only Spike-specific T-cell responses, in contrast to convalescent donors, who also exhibit T-cell responses against more conserved epitopes such as NP and M, which are less affected by viral mutations [103]. In our study, due to the low number of cases, we did not directly compare the clinical efficacy of ATT derived from convalescent versus vaccinated donors.

Donors are selected based on the highest frequency of virus-specific CD4 + and CD8 + T cells, directly influencing the IFN-γ + cell content of the final product. For IFN-γ CCS VST, the administered IFN-γ + T-cell dose varies across studies [30, 31, 51]. Clinical experience with expanded VSTs indicates improved outcomes with higher cell doses and repeated infusions. Therefore, maximizing IFN-γ + T-cell dose per infusion is critical. Importantly, the proportion of alloreactive non-IFN-γ + T cells must remain below the GVHD threshold [47, 48]. Lower IFN-γ + T-cell doses may reduce clinical efficacy but allow treatment of approximately 9–10 recipients per single IFN-γ CCS VST product [31]. However, if dosing is constrained by the non-IFN-γ + T-cell GVHD threshold, one IFN-γ CCS VST product suffices for treatment of only one or, at most, two patients. Based on these parameters, the creation of cryopreserved off-the-shelf biobanks is limited to small case numbers. In contrast, CD45RA^+^ TCD DLI allows production from a single 5-L apheresis unit sufficient for 9–10 patients at 1 × 10⁶ CD45RA-T cells/kg. Large-volume apheresis can supply 10–30 patients within 24 h, making it suitable for off-the-shelf biobanks for high-risk populations [71]. In pandemic scenarios, the production of IFN-γ CCS VST is time-consuming and requires antigen-specific peptide stimulation (e.g., PepTivator), whereas CD45RA^+^ TCD DLI is readily available, requiring only a convalescent donor. Here, the most common HLA alleles within the target population and HLA alleles relevant for certain viruses can be taken into consideration. The CD45RA^+^ TCD DLI comprises complex memory T-cell populations with long lifespan and capacity for re-expansion. It remains uncertain how long partially HLA-mismatched T-cells persist in recipients, but data from Release studies indicate persistence for at least four weeks. The therapy can be repeated as needed. The optimal dose of 1 × 10^6^ CD45RA-T cells/kg for infection treatment is not yet established but represents the current standard. Defining the precise dose will be a focus of future studies. In summary, in the context of pandemic preparedness, convalescent donors should be prioritized, and regardless of donor screening, CD45RA^+^ TCD DLI appears to be the most suitable option for the large-scale generation of off-the-shelf ATT.

### Limitations of the study

Our study has several limitations, despite being a prospective, single-center investigation comparing two SARS-CoV-2-specific ATT for the treatment of immunocompromised patients. First, the study was not randomized. Second, the enrolled patient number was low. Third, the two ATT modalities were not compared with immunocompromised patients receiving SoC treatment. Furthermore, subgroup analyses were limited by insufficient sample sizes for statistical comparisons. This subgroup analysis was conducted in an exploratory manner, designed primarily to generate preliminary hypotheses regarding age-related trends in therapeutic response and immune reconstitution. Although detailed assessments of viral clearance, flow cytometry, and cytokine/chemokine profiling were performed, the number of samples collected between weeks 5–8 was low. Additionally, our study was limited by using unsorted T cells for microchimerism measuring, which affected detection sensitivity. Nevertheless, this represents the first prospective study to compare the outcomes of two promising ATT approaches for the treatment of immunocompromised patients infected with SARS-CoV-2, including those who have undergone HSCT. Both ATT proved to be safe and effective treatment modalities. The OS at both 28 days and 1 year was similar between the two groups. However, COVID-19 relapse-free survival was superior in the CD45RA^+^ TCD DLI group. Among patients older than 50 years, CD45RA^+^ TCD DLI demonstrated better 1-year OS. In patients younger than 50 years, both ATT approaches showed adequate 1-year OS, with slightly better outcomes observed in the IFN-γ CCS VST group.

### Conclusions and future directions

During the COVID-19 pandemic, elderly, comorbid, and immunocompromised populations exhibited suboptimal responses to vaccines and antiviral therapies, highlighting the urgent need for effective and immediately applicable treatment options. In this prospective, single-center comparison, both IFN-γ cytokine capture system virus-specific T-cell therapy (IFN-γ CCS VST) and CD45RA-positive T-cell depleted donor lymphocyte infusion (CD45RA^+^ TCD DLI) proved safe and effective, leading to viral clearance without graft-versus-host disease or severe toxicities. Importantly, IFN-γ CCS VST elicited stronger SARS-CoV-2–specific immune responses, whereas CD45RA^+^ TCD DLI showed a survival advantage in HSCT recipients and demonstrated practical advantages for large-scale application. From a translational perspective, IFN-γ CCS VST requires antigen-specific stimulation and individualized donor selection, which is time-consuming and resource-intensive. In contrast, CD45RA^+^ TCD DLI can be prepared more rapidly through simple depletion of naïve T cells and is well suited for cryopreserved off-the-shelf banking. This feature makes CD45RA^+^ TCD DLI particularly attractive for pandemic preparedness, where rapid availability and broad treatment accessibility are critical. Beyond these logistical considerations, adoptive T-cell therapies directly address fundamental mechanisms of immunosenescence and virus-induced senescence that compromise antiviral immunity in aging populations. Furthermore, combining ATT with senolytic pharmacological treatments and engineered T-cell therapies (such as senolytic CAR-T cells, NK cells, or regulatory T cells) targeting senescence may further enhance clinical efficacy. By restoring functional virus-specific or memory T-cell responses, these therapies not only provide a viable strategy for persistent SARS-CoV-2 infection but also represent a broader model for overcoming age-related immune dysfunction. Drawing on both published evidence and our own clinical experience, we believe that in elderly and/or comorbid patients—including those with hematologic malignancies, HSCT, or SOT—both adoptive T-cell therapy approaches are feasible, safe, and effective. The next step toward broader clinical translation will be the development of cryopreserved, off-the-shelf biobanks supported by robust interdisciplinary collaboration. Future randomized multicenter studies in larger cohorts will be essential to validate these findings, define optimal patient selection, and explore integration of adoptive T-cell therapy with geroscience-guided interventions.

## Supplementary Information

Below is the link to the electronic supplementary material.

ESM 1 Nasopharyngeal PCR clearance after SARS-CoV-2 specific IFN-γ CCS VST and CD45RA+ TCD DLI therapy, A: Nasopharyngeal PCR clearance after IFN-γ CCS VST. B: Nasophranygeal PCR clearance after CD45RA+ TCD DLI. Abbreviations: PCR: polymerase chain reaction; IFN-γ CCS: interferon-γ cytokine capture system; VST: virus-specific T-cells; TCD: T-cell depletion; DLI: donor memory T-cell infusion Note: In the CD45RA+ TCD DLI group, case 7 with persistent SARS-CoV-2 positivity and poor graft function after the 1 st third-party cryopreserved CD45RA+ TCD DLI received CD34+ positively selected and CD45RA+ depleted T-cell booster from her original haploidentical donor. (PDF 41.3 KB)

ESM 2 (PDF 42.2 KB)

ESM 3 Characterization of Peripheral Blood Lymphocyte Subpopulations by Flow Cytometry Following Adoptive T-Cell Therapy: IFN-γ CCS VST Versus CD45RA+ TCD DLI. A: Statistical comparisons of lymphocyte subpopulations percentages for both cohorts. B: Changes in T-cells and NK-cells. C: Changes in CD4+ and CD8+ naive T-cell compartments. D: Changes in CD4+ and CD8+ memory T-cell compartments. Note: yellow background: decreased value; pale blue background: normal value; pink background: elevated value. Abbreviations: VST: virus-specific T- cells; IFN-γ CCS: interferon-γ cytokine capture system; TCD: T-cell depleted; DLI: donor lymphocyte infusion; T-reg: regulatory T-cell. (PDF 125 KB)

ESM 4 (PDF 56.3 KB)

ESM 5 (PDF 54.9 KB)

ESM 6 (PDF 55.3 KB)

ESM 7 Comparison of changes in multicytokine and chemokine patterns following IFN-γ CCS VST versus CD45RA+ depleted DLI therapy. A: Statistical comparisons of cytokine and chemokine values for both cohorts. B: Changes in IL-5 and IL-6 levels. C: Changes in IL-8 and IL-10 levels. D: Changes in IL-15 and RANTES levels. E: Changes in IP-10 and IFNγ levels. F: Changes in MIP-1α and MCP-1 levels. Note: pale gray background: decreased value; yellow background: normal value; orange background: >1-<2x upper normal value; light blue background: >2-<5x upper normal value; pinke background: >5-<10x upper normal value; purple background: >10-<50x upper normal value; dark blue background: >50-<100x upper normal value; red background: >100x upper normal value. Abbreviations: VST: virus specific T-cell; IFN-γ CCS: interferon-γ cytokine capture system; CD45RA+ TCD DLI: CD45RA+ T-cell depleted donor lymphocyte infusion; IFN: interferon; IL: interleukin; RANTES: regulated upon activation, normal T-cell expressed and secreted; CCL-5: C-C motif ligand 5; MCP-1: monocyte chemoattractant protein-1; IP-10: interferonγ-induced protein 10 kDa; CXCL10: C-X-C motif chemokine ligand 10; TNF: tumor necrosis factor. (PDF 169 KB)

ESM 8 (PDF 52.4 KB)

ESM 9 (PDF 50.5 KB)

ESM 10 (PDF 51.1 KB)

ESM 11 (PDF 59.9 KB)

ESM 12 (PDF 58.5 KB)

ESM 13 (DOCX 37.8 KB)

ESM 14 (DOCX 20.8 KB)

## Data Availability

Not applicable.

## Abbreviations

ACE: Angiotensin-converting enzyme
ADV: Adenovirus
ATMP: Advanced Therapy Medicinal Products
ATT: Adoptive T-cell transfer
BK: Baumann-Krech virus
CAR: Chimeric antigen receptor
CCS: Cytokine capture system
CD45RA + TCD DLI: CD45RA + T-cell-depleted donor lymphocyte infusion
CDR3: Complementary determining region 3
CIK-T: Cytokine-induced killer T cell
CMNC: Continuous Mononuclear Cell Collection
CMV: Cytomegalovirus
COVID-19: Coronavirus disease 2019
CRA: Cytokine release assay
CRS: Cytokine release syndrome
CT: Computed tomography
CTL: Cytotoxic T lymphocyte
CXCL10: C-X-C motif chemokine ligand 10
ddDIP: Digital droplet deletion-insertion (indel) PCR
DLI: Donor lymphocyte infusion
DLT: Dose-limiting toxicity
DNA: Deoxyribonucleic acid
EBV: Epstein Barr virus
ELISpot: Enzyme-Linked Immunospot
EUR: Euro
FACS: Flow cytometric analysis
FAP: Fibroblast activation protein
GMO: Genetically modified organism
GVHD: Graft-versus-host disease
HHV-6: Human herpesvirus 6
HLA: Human leukocyte antigen
HSCT: Hematopoietic stem cell transplantation
ICU: Intensive care unit
IFN-γ: Interferon-γ
IL: Interleukin
IP-10: Interferonγ-induced protein 10 kDa
JCV: John Cunningham virus
LOS: Length of stay
MCP-1: Monocyte chemoattractant protein-1
MHC: Major histocompatibility complex
MIP-1α: Macrophage-inflammatory protein-1α
MSC: Mesenchymal stem cell
MUD: Matched unrelated donor
NA: Not applicable
NK: Natural killer cell
NKG2D: Natural Killer Group 2 Member D
OS: Overall survival
OVSZ: Hungarian National Blood Transfusion Service
PBMC: Peripheral blood mononuclear cells
PD-1: Programmed cell death protein 1
RANTES: Regulated upon activation, normal T–cell expressed and secreted
RFS: Relapse-free survival
RI: Relapse incidence
RT-PCR: Real-time polymerase chain reaction
SARS-CoV-2: Severe Acute Respiratory Syndrome Coronavirus 2
SASP: Senescence-associated secretory phenotype
sHLH: Secondary hemophagocytic lymphohistiocytosis
SOC: Standard-of-care
SOT: Solid organ transplantation
SPCH–NIHID: South Pest Central Hospital, National Institute of Hematology and Infectious Diseases
TCR: T-cell receptor
TIM-3: T cell immunoglobulin and mucin domain-containing protein 3
TNF: Tumor necrosis factor
VIS: Virus induced senescence
VST: Virus specific T-cell
USD: United states dollar

## References

[1] Msemburi W, Karlinsky A, Knutson V, Aleshin-Guendel S, Chatterji S, Wakefield J. The WHO estimates of excess mortality associated with the COVID-19 pandemic. Nature. 2023;613:130–7. 10.1038/s41586-022-05522-2. 36517599 10.1038/s41586-022-05522-2 PMC9812776

[2] WHO warns of slowing global health gains in new statistics report [WHO Web site]. 2025. https://www.who.int/news/item/15-05-2025-who-warns-of-slowing-global-health-gains-in-new-statistics-report. Accessed 15 May 2025.

[3] Docherty AB, Harrison EM, Green CA, Hardwick HE, Pius R, Norman L, et al. Features of 20133 UK patients in hospital with covid-19 using the ISARIC WHO clinical characterisation protocol: prospective observational cohort study. BMJ. 2020;369:m1985. 10.1136/bmj.m1985. 32444460 10.1136/bmj.m1985 PMC7243036

[4] Wemhöner L, Brandts C, Dinse H, Skoda E-M, Jansen S, Teufel M, et al. Consequences of COVID-19 for geriatric patients during a pandemic. Sci Rep. 2025;15:3136. 10.1038/s41598-024-84379-z. 39856128 10.1038/s41598-024-84379-z PMC11759943

[5] COVID-19 -Global situation [WHO Web site] 2025. https://www.who.int/emergencies/disease-outbreak-news/item/2025-DON572. Accessed 28 May 2025.

[6] Bartha I, Maher C, Lavrenko V, Chen Y-P, Tao Q, di Iulio J, et al. Morbidity of SARS-CoV-2 in the evolution to endemicity and in comparison with influenza. Commun Med. 2024;4:244. 10.1038/s43856-024-00633-5. 39578575 10.1038/s43856-024-00633-5 PMC11584631

[7] Lippi G, Sanchis-Gomar F. Mortality of post-COVID-19 condition: 2025 update. COVID. 2025;5:11. 10.3390/covid5010011.

[8] Turtle L, Thorpe M, Drake TM, Swets M, Palmieri C, Russell CD, et al. Outcome of COVID-19 in hospitalised immunocompromised patients: an analysis of the WHO ISARIC CCP-UK prospective cohort study. PLoS Med. 2023;20:e1004086. 10.1371/journalpmed.1004086. 36719907 10.1371/journal.pmed.1004086 PMC9928075

[9] Loubet P, Benotmane I, Fourati S, Malard F, Vuotto F, Blanchard E, et al. Avouac. Risk of severe COVID-19 in four immunocompromised populations: a french expert perspective. Infect Dis Ther. 2025;14:671–733. 10.1007/s40121-025-01124-3. 40100618 10.1007/s40121-025-01124-3 PMC11993528

[10] Choi B, Choudhary MC, Regan J, Sparks JA, Padera RF, Qiu X, et al. Persistence and evolution of SARS-CoV-2 in an immunocompromised host. N Engl J Med. 2020;383:2291–3. 10.1056/NEJMc2031364. 33176080 10.1056/NEJMc2031364 PMC7673303

[11] Li Y, Choudhary MC, Regan J, Boucau J, Nathan A, Speidel T, et al. SARS-CoV-2 viral clearance and evolution varies by extent of immunodeficiency. medRXiv. 2023:23293441. 10.1101/2023.07.31.23293441.

[12] Feng S, Reid GE, Clark NM, Harrington A, Uprichard SL, Baker SC. Evidence of SARS-CoV-2 convergent evolution in immunosuppressed patients treated with antiviral therapies. Virol J. 2024;21:105. 10.1186/s12985-024-02378-y. 38715113 10.1186/s12985-024-02378-y PMC11075269

[13] Marques AD, Graham-Wooten J, Fitzgerald AS, Leonard AS, Cook EJ, Everett JK, et al. SARS-CoV-2 evolution during prolonged infection in immunocompromised patients. MBio. 2024;15:e00110-24. 10.1128/mbio.00110-24. 38364100 10.1128/mbio.00110-24 PMC10936176

[14] Gulick RM, Pau AK, Daar E, Evans L, Gandhi RT, Tebas P, et al. National Institutes of Health COVID-19 treatment guidelines panel: perspectives and lessons learned. Ann Intern Med. 2024;177:1547–57. 10.7326/ANNALS-24-00464. 39348691 10.7326/ANNALS-24-00464 PMC13107399

[15] Caso JM, Fernández-Ruiz M, López-Medrano F, Caro-Teller JM, Lizasoain M, San-Juan R, et al. Nirmatrelvir/ritonavir for the treatment of immunocompromised adult patients with early‐stage symptomatic COVID‐19: a real‐life experience. J Med Virol. 2023;95:e29082. 37671852 10.1002/jmv.29082

[16] Cesaro S, Ljungman P, Mikulska M, Hirsch HH, Navarro D, Cordonnier C, et al. Post-pandemic recommendations for the management of COVID-19 in patients with haematological malignancies or undergoing cellular therapy, from the European Conference on Infections in Leukaemia (ECIL-10). Leukemia. 2025. 10.1038/s41375-025-02649-9. 40456838 10.1038/s41375-025-02649-9 PMC12380621

[17] Kim N, Lee J-M, Oh E-J, Jekarl DW, Lee D-G, Im K-I, et al. Off-the-shelf partial HLA matching SARS-CoV-2 antigen specific T cell therapy: a new possibility for COVID-19 treatment. Front Immunol. 2021;12:751869. 10.3389/fimmu.2021.751869. 35003063 10.3389/fimmu.2021.751869 PMC8733616

[18] Guerreiro M, Aguilar‐Gallardo C, Montoro J, Francés‐Gómez C, Latorre V, Luna I, Planelles D, Carrasco MP, Gómez MD, González-Barberá EM, Aguardo C, Sempere A, Solves P, Gómez-Seguí I, Balaguer-Rosello A, Louro A, Perla A, Larrea L, Sanz J, Arbona C, de la Rubia, Geller R, Sanz MÁ, Sanz G, Piñana. Adoptive Transfer of Ex Vivo Expanded SARS‐CoV‐2‐specific Cytotoxic Lymphocytes: A Viable Strategy for COVID‐19 Immunosuppressed Patients? Transpl Infect Dis. 2021;23: e13602. 10.1111/tid.13602. 10.1111/tid.13602 PMC8250091 33728702

[19] Sivapalan R, Liu J, Chakraborty K, Arthofer E, Choudhry M, Barie PS, et al. Virus induced lymphocytes (VIL) as a novel viral antigen-specific T cell therapy for COVID-19 and potential future pandemics. Sci Rep. 2021;11:15295. 10.1038/s41598-021-94654-y. 34315945 10.1038/s41598-021-94654-y PMC8316478

[20] Mora-Buch R, Tomás-Marín T, Enrich E, Antón-Iborra M, Martorell L, Valdivia E, et al. Virus-specific T cells from cryopreserved blood during an emergent virus outbreak for a potential off-the-shelf therapy. Transplant Cell Ther. 2023;29:572.e1-572.e13. 10.1016/j.jtct.2023.06.001. 37290691 10.1016/j.jtct.2023.06.001

[21] Al-Akioui-Sanz K, Pascual-Miguel B, Díaz-Almirón M, Mestre-Durán C, Navarro-Zapata A, Clares-Villa L, et al. Donor selection for adoptive cell therapy with CD45RA− memory T cells for patients with coronavirus disease 2019, and dexamethasone and interleukin-15 effects on the phenotype, proliferation and interferon gamma release. Cytotherapy. 2023;25:330–40. 10.1016/j.jcyt.2022.12.001. 36585293 10.1016/j.jcyt.2022.12.001 PMC9742221

[22] Papadopoulou A, Gerdemann U, Katari UL, Tzannou I, Liu H, Martinez C, et al. Activity of broad-spectrum T cells as treatment for AdV, EBV, CMV, BKV, and HHV6 infections after HSCT. Sci Transl Med. 2014. 10.1126/scitranslmed.3008825. 24964991 10.1126/scitranslmed.3008825 PMC4181611

[23] Gottschalk S, Rooney CM. Adoptive T cell immunotherapy. Curr Top Microbiol Immunol. 2015;391:427–54. 10.1007/978-3-319-22834-1_15. 26428384 10.1007/978-3-319-22834-1_15 PMC4655436

[24] Jiang W, Clancy LE, Avdic S, Sutrave G, Street J, Simms R, et al. Third-party CMV- and EBV-specific T-cells for first viral reactivation after allogeneic stem cell transplant. Blood Adv. 2022(17). 10.1182/bloodadvances.2022007103. 10.1182/bloodadvances.2022007103 PMC9631614 35819448

[25] O’Reilly RJ. T-cells: third party parity for viral infections. Transplant Cell Ther. 2023;29:285–6. 10.1016/j.jtct.2023.03.031. 37120252 10.1016/j.jtct.2023.03.031

[26] Tzannou I, Papadopoulou A, Naik S, Leung K, Martinez CA, Ramos CA, et al. Off-the-shelf virus-specific T cells to treat BK virus, human herpesvirus 6, cytomegalovirus, Epstein-Barr virus, and adenovirus infections after allogeneic hematopoietic stem-cell transplantation. J Clin Oncol. 2017;35:3547–57. 10.1200/JCO.2017.73.0655. 28783452 10.1200/JCO.2017.73.0655 PMC5662844

[27] Pfeiffer T, Tzannou I, Wu M, Ramos C, Sasa G, Martinez C, et al. Posoleucel, an allogeneic, off-the-shelf multivirus-specific T-cell therapy, for the treatment of refractory viral infections in the post-HCT setting. Clin Cancer Res. 2023;29:324–30. 10.1158/1078-0432.CCR-22-2415. 36628536 10.1158/1078-0432.CCR-22-2415 PMC9843433

[28] Dadwal SS, Bansal R, Schuster MW, Yared JA, Myers GD, Matzko M, et al. Final outcomes from a phase 2 trial of posoleucel in allogeneic hematopoietic cell transplant recipient. Blood Adv. 2024;8:4740–50. 10.1182/bloodadvances.2023011562. 38593233 10.1182/bloodadvances.2023011562 PMC11413696

[29] Green A, Rubinstein JD, Grimley M, Pfeiffer T. Virus-specific T cells for the treatment of systemic infections following allogeneic hematopoietic cell and solid organ transplantation. J Pediatr Infect Dis Soc. 2024;13(Supplement_1):S49-57. 10.1093/jpids/piad077. 10.1093/jpids/piad077 38417086

[30] Gopcsa L, Réti M, Andrikovics H, Bobek I, Bekő G, Bogyó J, Ceglédi A, Dobos K, Giba-Kiss Laura, Jankovics I, Kis O, Lakatos B, Mathiász D, Meggyesi N, Miskolczi G, Németh N, Paksi M, Riczu A, Sinkó J, Szabó B, Szilvási A, Szlávik J, Szabolcs Tasnády Sz, Reményi P, Vályi-Nagy I. Effective virus-specific T-cell therapy for high-risk SARS-CoV-2 infections in hematopoietic stem cell transplant recipients: initial case studies and literature review. Geroscience. 2024;46: 1083–1106. 10.1007/s11357-023-00858-7. 10.1007/s11357-023-00858-7 PMC10828167 37414968

[31] Seng MS-F, Ng KP, Soh TG, Tan TT, Chan M, Maiwald M, et al. A phase I/II study of adoptive SARS-CoV-2-specific T cells in immunocompromised hosts with or at risk of severe COVID-19 infection. Cytotherapy. 2024;26:1170–8. 10.1016/j.jcyt.2024.05.014. 38864802 10.1016/j.jcyt.2024.05.014

[32] Talleur AC, Li Y, Akel S, Sharma A, Qudeimat A, Srinivasan A, et al. Haploidentical CD45RA-negative donor lymphocyte infusions are feasible, safe and associated with clinical benefit. Biol Blood Marrow Transplant. 2020;26:S268. 10.1016/j.bbmt.2019.12.435.

[33] Gasior Kabat M, Bueno D, Sisinni L, De Paz R, Mozo Y, Perona R, Arias-Salgado EG, Rosich B, Marcos A, Romero AB, Constanzo, Jiménez-Yuste V, Pérez-Martínez A. Selective T-Cell Depletion Targeting CD45RA as a Novel Approach for HLA-Mismatched Hematopoietic Stem Cell Transplantation in Pediatric Nonmalignant Hematological Diseases. Int J Hematol. 2021;114: 116–123. 10.1007/s12185-021-03138-2. 10.1007/s12185-021-03138-2 33772729

[34] Naik S, Triplett BM. Selective depletion of naïve T cells by targeting CD45RA. Front Oncol. 2023;12:1009143. 10.3389/fonc.2022.1009143. 36776371 10.3389/fonc.2022.1009143 PMC9911795

[35] Bleakley M, Heimfeld S, Jones LA, Turtle C, Krause D, Riddell SR, et al. Engineering human peripheral blood stem cell grafts that are depleted of naïve T cells and retain functional pathogen-specific memory T cells. Biol Blood Marrow Transplant. 2014;20:705–16. 10.1016/j.bbmt.2014.01.032. 24525279 10.1016/j.bbmt.2014.01.032 PMC3985542

[36] Bleakley M, Heimfeld S, Loeb KR, Jones LA, Chaney C, Seropian S, et al. Outcomes of acute leukemia patients transplanted with naive T cell–depleted stem cell grafts. J Clin Invest. 2015;125:2677–89. 10.1172/JCI81229. 26053664 10.1172/JCI81229 PMC4563691

[37] Bleakley M, Sehgal A, Seropian S, Biernacki MA, Krakow EF, Dahlberg A, et al. Naive T-cell depletion to prevent chronic graft-versus-host disease. J Clin Oncol. 2022;40:1174–85. 10.1200/JCO.21.01755. 35007144 10.1200/JCO.21.01755 PMC8987226

[38] Braidotti S, Granzotto M, Curci D, Kotnik BF, Maximova N. Advancing allogeneic hematopoietic stem cell transplantation outcomes through immunotherapy: a comprehensive review of optimizing non-CAR donor T-lymphocyte infusion strategies. Biomedicines. 2024;12:1853. 10.3390/biomedicines12081853. 39200317 10.3390/biomedicines12081853 PMC11351482

[39] Shelikhova L, Ilushina M, Shekhovtsova Z, Shasheleva D, Khismatullina R, Kurnikova E, et al. αβ T cell-depleted haploidentical hematopoietic stem cell transplantation without antithymocyte globulin in children with chemorefractory acute myelogenous leukemia. Biol Blood Marrow Transplant. 2019;25:e179–82. 10.1016/j.bbmt.2019.01.023. 30677509 10.1016/j.bbmt.2019.01.023

[40] Maung KK, Chen BJ, Barak I, Li Z, Rizzieri DA, Gasparetto C, Sullivan KM, Long GD, Engemann AM, Waters-Pick B, Nichols KR, Lopez R, Kang Y, Sarantopoulos, Sung AD, Chao NJ, Horwitz. Phase I Dose Escalation Study of Naive T-Cell Depleted Donor Lymphocyte Infusion Following Allogeneic Stem Cell Transplantation. Bone Marrow Transplant. 2021; 56: 137–43. 10.1038/s41409-020-0991-5. 10.1038/s41409-020-0991-5 PMC10805111 32624583

[41] Ferreras C, Pascual-Miguel B, Mestre-Durán C, Navaro-Zapata A, Clares-Villa L, Martín-Costázar C, et al. SARS-CoV-2-specific memory T lymphocytes from COVID-19 convalescent donors: identification, biobanking, and large-scale production for adoptive cell therapy. Front Cell Dev Biol. 2021;9:620730. 33718360 10.3389/fcell.2021.620730 PMC7947351

[42] García-García I, Guerra-García P, Ferreras C, Borobia AM, Carcas AJ, Queiruga-Parada J, et al. A phase I/II dose-escalation multi-center study to evaluate the safety of infusion of natural killer cells or memory T cells as adoptive therapy in coronavirus pneumonia and/or lymphopenia: RELEASE study protocol. Trials. 2021;22:674. 34600562 10.1186/s13063-021-05625-7 PMC8487326

[43] Pérez-Martínez A, Mora-Rillo M, Ferreras C, Guerra-García P, Pascual-Miguel B, Mestre-Durán C, et al. Phase I dose-escalation single centre clinical trial to evaluate the safety of infusion of memory T cells as adoptive therapy in COVID-19 (RELEASE). EClinicalMedicine. 2021;39:101086. 34405140 10.1016/j.eclinm.2021.101086 PMC8361305

[44] Sanz KA-A Karima, Parente CE, Ferreras C, Ribes MM, Navarro A, Mestre C, et al. Familial CD45RA– T cells to treat severe refractory infections in immunocompromised patients. Front Med. 2023;10:1083215. 10.3389/fmed.2023.1083215. 10.3389/fmed.2023.1083215 PMC9944023 36844219

[45] Chin HJ, Ryu YH, Kang DY, Park HJ, Hong KT, Choi JY, et al. CD45RA+ depleted lymphocyte infusion for treatment of refractory cytomegalovirus disease in complete DiGeorge syndrome: a case report. Pediatric Infection & Vaccine. 2023;30:173–9. 10.14776/piv.2023.30.e18.

[46] Zhang M, Luo C, Wang J, Zhu H, Luo C, Qin X, et al. TCRαβ-depleted hematopoietic stem cell transplant and third-party CD45RA+ depleted adoptive cell therapy for treatment of post-transplant parvovirus B19 aplastic crisis. Int J Infect Dis. 2024;144:107043. 10.1016/j.ijid.2024.107043. 38583826 10.1016/j.ijid.2024.107043

[47] Federmann B, Bornhauser M, Meisner C, Kordelas L, Beelen DW, Stuhler G, et al. Haploidentical allogneic hematopoietic cell transplantation in adults using CD3/CD19 depletion and reduced intensity conditioning: a phase II study. Haematologica. 2012;97:1523–31. 10.3324/haematol.2011.059378. 22491731 10.3324/haematol.2011.059378 PMC3487553

[48] Zhang P, Tey S-K. Adoptive T cell therapy following haploidentical hematopoietic stem cell transplantation. Front Immunol. 2019;10:1854. 10.3389/fimmu.2019.01854. 31447852 10.3389/fimmu.2019.01854 PMC6691120

[49] Gopcsa L, Bobek I, Bekő G, Lakatos B, Molnár E, Réti M, et al. Common points of therapeutic intervention in COVID-19 and in allogeneic hematopoietic stem cell transplantation associated severe cytokine release syndrome. Acta Microbiol Immunol Hung. 2021;68:240–55. 34797216 10.1556/030.2021.01620

[50] Morris AB, Bray R, Gebel HM, Sullivan HC. A primer on chimerism analysis: a straightforward, thorough review. Lab Med. 2023;54:352–62. 36374737 10.1093/labmed/lmac132

[51] Flower A, Ayello J, Harrison L, Morris E, Sturhahn M, Maryamchik E, et al. The safety and efficacy of targeted virus specific cytotoxic T-lymphocytes (VST) manufactured by the IFN-g Cytokine Capture System (CCS) for the treatment of refractory adenovirus (ADV), cytomegalovirus (CMV), Epstein Barr virus (EBV) and BK virus (BKV) in children, adolescents and young adults (CAYA) after allogeneic hematopoietic stem cell transplantation (Allo-HSCT), solid organ transplantation (SOT), or with primary immunodeficiency (PID) (IND#17449). Biol Blood Marrow Transplant. 2020;26:8–74. 10.1016/j.bbmt.2019.12.220.

[52] Arevalo-Romero JA, Chingaté-López SM, Camacho BA, Alméciga-Díaz CJ, Ramirez-Segura. Nex-generation treatments: Immunotherapy and advanced therapies for COVID-19. Heliyon. 2024;10: e26423. 10.1016/j.heliyon.2024.e26423. 10.1016/j.heliyon.2024.e26423 PMC10907543 38434363

[53] Ljungman P, de la Camara R, Mikulska M, Tridello G, Aguado B, Al Zahrani M, Apperley J, Berceanu A, Bofarull RM, Calbacho M, Ciceri F, Lopez-Corral L, Crippa C, Fox ML, Grassi A, Jimenez M-J, Demir SK, Kwon M, Llamas CV, Lorenzo JLL, Mielke S, Orchard K, Porras RP, Vallisa D, Xhaard A, Knelange NS, Cedillo A, Kröger N, Piňana JL, Styczynski. COVID-19 and stem cell transplantation; results from an EBMT and GETH multicenter prospective survey. Leukemia. 2021;35: 2885–94. 10.1038/s41375-021-01302-5. 10.1038/s41375-021-01302-5 PMC8171362 34079042

[54] Ljungman P, Tridello G, Piňana JL, Ciceri F, Sengeloev H, Kulagin A, et al. Improved outcomes over time and higher mortality in CMV seropositive allogeneic stem cell transplantation patients with COVID-19; an infectious disease working party study from the European Society for Blood and Marrow Transplantation registry. Front Immunol. 2023;14:1125824. 10.3389/fimmu.2023.1125824. 36960069 10.3389/fimmu.2023.1125824 PMC10028143

[55] Machkovech HM, Hahn AM, Wang JG, Grubaugh ND, Halfmann PJ, Johnson MC, et al. Persistent SARS-CoV-2 infection: significance and implications. Lancet Infect Dis. 2024;24:e453–62. 10.1016/S1473-3099(23)00815-0. 38340735 10.1016/S1473-3099(23)00815-0

[56] Hettle D, Hutchings S, Muir P, Moran E. Persistent SARS-CoV-2 infection in immunocompromised patients facilitates rapid viral evolution: retrospective cohort study and literature review. Clinical Infection in Practice. 2022;16:100210. 10.1016/j.clinpr.2022.100210. 36405361 10.1016/j.clinpr.2022.100210 PMC9666269

[57] Nooruzzaman M, Johnson KEE, Rani R, Finkelsztein EJ, Caserta LC, Kodiyanplakkal RP, et al. Emergence of transmissible SARS-CoV-2 variants with decreased sensitivity to antivirals in immunocompromised patients with persistent infections. Nat Commun. 2024;15:7999. 10.1038/s41467-024-51924-3. 39294134 10.1038/s41467-024-51924-3 PMC11411086

[58] Siddiqui AN, Musharaf I, Gulumbe BH. The JN.1 variant of COVID-19: immune evasion, transmissibility, and implications for global health. Ther Adv Infect Dis. 2025;12:20499361251314764. 10.1177/20499361251314763. 10.1177/20499361251314763 PMC11783492 39896217

[59] Nardo D, Maddox EG, Riley JL. Cell therapies for viral diseases: a new frontier. Semin Immunopathol. 2025;47:5. 10.1007/s00281-024-01031-8. 39747475 10.1007/s00281-024-01031-8 PMC11695571

[60] Koukoulias K, Papayanni PG, Leen AM, Vasileiou S. Virus-specific T-cell therapy for the management of viral infections in the immunocompromised. Transfus Med Hemother. 2024;52:5–26. 10.1159/000540961. 39944414 10.1159/000540961 PMC11813280

[61] Chen Y, Liu C, Fang Y, Chen W, Qiu J, Zhu M, et al. Developing CAR-immune cell therapy against SARS-CoV-2: current status, challenges and prospects. Biochem Pharmacol. 2024;222:116066. 10.1016/j.bcp.2024.116066. 38373592 10.1016/j.bcp.2024.116066

[62] Campbell C, Rudensky A. Roles of regulatory T cells in tissue pathophysiology and metabolism. Cell Metab. 2020;31:18–25. 10.1016/j.cmet.2019.09.010. 31607562 10.1016/j.cmet.2019.09.010 PMC7657366

[63] Loy H, Kuok DIT, Hui KPY, Choi MHL, Yuen W, Nicholls JM, et al. Therapeutic implications of human umbilical cord mesenchymal stromal cells in attenuating influenza A(H5N1) virus–associated acute lung injury. J Infect Dis. 2019;219:186–96. 10.1093/infdis/jiy478. 30085072 10.1093/infdis/jiy478 PMC6306016

[64] Yan C, Hu M, Dai R. Safety and efficacy of mesenchymal stem cells in COVID-19 patients: a systematic review and meta-analysis. Immunity Inflamm Dis. 2023;11:e1000. 10.1002/iid3.1000. 10.1002/iid3.1000 PMC10515507 37773722

[65] Martits-Chalangari K, Spak CW, Askar M, Killian A, Fisher TL, Atillasoy E, et al. ALVR109, an off-the-shelf partially HLA matched SARS-CoV-2-specific T cell therapy, to treat refractory severe COVID-19 pneumonia in a heart transplant patient: case report. Am J Transplant. 2022;22:1261–5. 10.1111/ajt.16927. 34910857 10.1111/ajt.16927 PMC9303326

[66] Vasileiou S, Hill L, Kuvalekar M, Workineh AG, Watanabe A, Velazquez Y, et al. Allogeneic, off-the-shelf, SARS-CoV-2-specific T cells (ALVR109) for the treatment of COVID-19 in high risk patients. Haematologica. 2022;108:1840–50. 10.3324/haematol.2022.281946. 10.3324/haematol.2022.281946 PMC10316279 36373249

[67] Haidar G, Jacobs JL, Hughes Kramer K, Naqvi A, Heaps A, Parikh U, et al. Therapy with allogeneic SARS-CoV-2-specific T-cells for persistent COVID-19 in immunocompromised patients. Clin Infect Dis. 2023;77:696–702. 10.1093/cid/ciad233. 37078720 10.1093/cid/ciad233 PMC10495124

[68] Papadopoulou A, Karavalakis G, Papadopoulou E, Xochelli A, Bousiou Z, Vogiatzoglou A, et al. SARS-CoV-2-specific T cell therapy for severe COVID-19: a randomized phase 1/2 trial. Nat Med. 2023;29:2019–29. 10.1038/s41591-023-02480-8. 37460756 10.1038/s41591-023-02480-8

[69] Lee R, Kim N, Kim W-B, Im K-I, Nho D, Cho S-Y, Park C, Beck KS, Lee GH, Lee I, Cho S-G, Lee D-G. Effectiveness and safety of autologous virus-specific T cell therapy for persistent COVID-19 in people with immunocompromise: A Clinical Trial Study. Clin Infect Dis. 2025; ciaf302. 10.1093/cid/ciaf302. 10.1093/cid/ciaf302 40493744

[70] Grosso D, Wagner JL, O’Connor A, Keck K, Huang Y, Wang Z-X, et al. Safety and feasibility of third-party cytotoxic T lymphocytes for high-risk patients with COVID-19. Blood Adv. 2024;8:4113–24. 10.1182/bloodadvances.2024013344. 38885482 10.1182/bloodadvances.2024013344 PMC11345373

[71] Ferreras C, Hernández-Blanco C, Martín-Quirós A, Al-Akioui-Sanz K, Mora-Rillo M, Ibáñez F, et al. Results of phase 2 randomized multi-center study to evaluate the safety and efficacy of infusion of memory T cells as adoptive therapy in severe acute respiratory syndrome coronavirus 2 (SARS-CoV-2) pneumonia and/or lymphopenia (RELEASE NCT04578210). Cytotherapy. 2024;26:25–35. 10.1016/j.jcyt.2023.10.002. 37897472 10.1016/j.jcyt.2023.10.002

[72] Li C, Xu D, Lu L, Peng S, Zhao H, Zeng C, et al. Clinical impact of concurrent autologous adoptive T cells immunotherapy in acitve COVID-19 infected cancer patients for chmeotherapy. Infect Agent Cancer. 2025;20:23. 40205403 10.1186/s13027-025-00654-2 PMC11983847

[73] Dunaikina M, Zhekhovtsova Z, Shelikhova L, Glushkova S, Nikolaev R, Blagov S, et al. Safety and efficacy of the low-dose memory (CD45RA-depleted) donor lymphocyte infusion in recipients of αβ T cell-depleted haploidentical grafts: results of a prospective randomized trial in high-risk childhood leukemia. Bone Marrow Transplant. 2021;56:1614–24. 10.1038/s41409-021-01232-x. 33594278 10.1038/s41409-021-01232-x

[74] Castagna L, Valli V, Timofeeva I, Capizzuto R, Bramanti S, Mariotti J, et al. Feasibility and efficacy of CD45RA+ depleted donor lymphocytes infusion after haploidentical transplantation with post-transplantation cyclophosphamide in patients with hematological malignancies. Transplantation and Cellular Therapy. 2021;27:478.e1-478.e5. 10.1016/j.jtct.2021.03.010. 33819481 10.1016/j.jtct.2021.03.010

[75] Pai JA. High-throughput and single-cell T cell receptor sequencing technologies. Nat Methods. 2021;18:881–92. 10.1038/s41592-021-01201-8. 34282327 10.1038/s41592-021-01201-8 PMC9345561

[76] Wu J, Zhang L, Zhao Z, Liu Y, Li Z, Feng X, et al. Advancing T-cell immunotherapy for cellular senescence and disease: mechanisms, challenges, and clinical prospects. Ageing Res Rev. 2025;109:102783. 10.1016/j.arr.2025.102783. 40412763 10.1016/j.arr.2025.102783

[77] Wang S, Huo T, Lu M, Zhao Y, Zhang J, He W, et al. Recent advances in aging and immunosenescence: mechanisms and therapeutic strategies. Cells. 2025;14:499. 10.3390/cells14070499. 40214453 10.3390/cells14070499 PMC11987807

[78] Hayflick L. The limited in vitro lifetime of human diploid cell strains. Exp Cell Res. 1965;37:614–36. 10.1016/0014-4827(65)90211-9. 14315085 10.1016/0014-4827(65)90211-9

[79] Matveeva K, Vasilieva M, Minskaia E, Rybtsov S, Shevyrev D. T-cell immunity against senescence: potential role and perspectives. Front Immunol. 2024;15:1360109. 10.3389/fimmu.2024.1360109. 38504990 10.3389/fimmu.2024.1360109 PMC10948549

[80] Marin I, Boix O, Garcia-Garijo A, Sirois I, Caballe A, Zarzuela E, et al. Cellular senescence is immunogenic and promotes antitumor immunity. Cancer Discov. 2023;13:410–31. 10.1158/2159-8290.CD-22-0523. 36302218 10.1158/2159-8290.CD-22-0523 PMC7614152

[81] Fu Z, Xu H, Yue L, Zheng W, Pan L, Gao F, et al. Immunosenescence and cancer: opportunities and challenges. Medicine. 2023;102:e36045. 10.1097/MD.0000000000036045. 38013358 10.1097/MD.0000000000036045 PMC10681516

[82] Gergues M, Bari R, Koppisetti S, Gosiewska A, Kang L, Hariri RJ. Senescence, NK cells, and cancer: navigating the crossroads of aging and disease. Front Immunol. 2025;16:1565278. 10.3389/fimmu.2025.1565278. 40255394 10.3389/fimmu.2025.1565278 PMC12006071

[83] Lord JM, Veenith T, Sullivan J, Sharma-Oates A, Richter AG, Greening NJ, et al. Accelerated immune ageing is associated with COVID-19 disease severity. Immun Ageing. 2024;21:6. 10.1186/s12979-023-00406-z. 38212801 10.1186/s12979-023-00406-z PMC10782727

[84] Baechle JJ, Chen N, Makhijani P, Winer S, Furman D, Winer DA. Chronic inflammation and the hallmarks of aging. Mol Metab. 2023;74:101755. 10.1016/j.molmet.2023.101755. 37329949 10.1016/j.molmet.2023.101755 PMC10359950

[85] Kirkland JL, Tchkonia T. Senolytic drugs: from discovery to translation. J Intern Med. 2020;288:518–36. 10.1111/joim.13141. 32686219 10.1111/joim.13141 PMC7405395

[86] Du PY, Gandhi A, Bawa M, Gromala J. The ageing immune system as a potential target of senolytics. Oxf Open Immunol. 2023;4:iqad004. 10.1093/oxfimm/iqad004. 37255929 10.1093/oxfimm/iqad004 PMC10191675

[87] Lelarge V, Capelle R, Oger F, Mathieu T, Le Calvé B. Senolytics: from pharmacological inhibitors to immunotherapies, a promising future for patients’ treatment. NPJ Aging. 2024;10:12. 10.1038/s41514-024-00138-4. 38321020 10.1038/s41514-024-00138-4 PMC10847408

[88] Amor C, Feucht J, Leibold J, Ho YJ, Zhu C, Alonso-Curbelo D, et al. Senolytic CAR T cells reverse senescence-associated pathologies. Nature. 2020;583:127–32. 10.1038/s41586-020-2403-9. 32555459 10.1038/s41586-020-2403-9 PMC7583560

[89] Yang D, Sun B, Li S, Wei W, Liu X, Cui X, et al. NKG2D-CAR T cells eliminate senescent cells in aged mice and nonhuman primates. Sci Transl Med. 2023;15:eadd1951. 10.1126/scitranslmed.add1951. 37585504 10.1126/scitranslmed.add1951

[90] Aghajanian H, Kimura T, Rurik JG, Hancock AS, Leibowitz MS, Li L, et al. Targeting cardiac fibrosis with engineered T cells. Nature. 2019;573:430–3. 10.1038/s41586-019-1546-z. 31511695 10.1038/s41586-019-1546-z PMC6752964

[91] Zhai P, Sadoshima J. Cardiomyocyte senescence and the potential therapeutic role of senolytics in the heart. J Cardiovasc Aging. 2024;4:18. 10.20517/jca.2024.06. 39119147 10.20517/jca.2024.06 PMC11309366

[92] Beers DR, Thonhoff JR, Faridar A, Thome AD, Zhao W, Wen S, et al. Tregs attenuate peripheral oxidative stress and acute phase proteins in ALS. Ann Neurol. 2022;92:195–200. 10.1002/ana.26375. 35445431 10.1002/ana.26375 PMC9545429

[93] Elyaman W, Stern L, Jiang N, Dressman D, Bradley P, Klatzmann D, et al. Exploring the role of T cells in Alzheimer’s and other neurodegenerative diseases: emerging therapeutic insights from the T cells in the brain symposium. Alzheimer’s & Dementia. 2025;21:e14548. 10.1002/alz.14548. 10.1002/alz.14548 PMC11851166 39868844

[94] Deng X, Terunuma H. Adoptive NK cell therapy: a potential revolutionary approach in longevity therapeutics. Immun Ageing. 2024;21:43. 10.1186/s12979-024-00451-2. 38926847 10.1186/s12979-024-00451-2 PMC11201368

[95] Chelyapov N, Nguyen TT, Gonzalez R. Autologous NK cells propagated and activated *ex vivo* decrease senescence markers in human PBMCs. Biochem Biophys Rep. 2022;32:101380. 10.1016/j.bbrep.2022.101380. 36386442 10.1016/j.bbrep.2022.101380 PMC9661662

[96] di d’Adda Fagagna F. SARS-CoV-2 causes DNA damage, cellular senescence andinflammation. Nat Cell Biol. 2023;25:526–7. 10.1038/s41556-023-01097-w. 36894672 10.1038/s41556-023-01097-w

[97] Bruno RM, Badhwar S, Abid L, Agharazii M, Anastasio F, Bellien J, et al. Accelerated vascular ageing after COVID-19 infection: the CARTESIAN study. Eur Heart J. 2025. 10.1093/eurheartj/ehaf430. 40819656 10.1093/eurheartj/ehaf430 PMC12517754

[98] Campisi M, Cannella L, Bordin A, Moretto A, Scapellato ML, Mason P, et al. Revealing the hidden impacts: insights into biological aging and long-term effects in pauci- and asymptomatic COVID-19 healthcare workers. Int J Mol Sci. 2024;25:8056. 10.3390/ijms25158056. 39125624 10.3390/ijms25158056 PMC11311509

[99] Morin S, Pradier A, Giannotti F, Mamez A-C, Vu-Cantero D-L, Fabra-Urdiola M, et al. Expansion of phenotypically senescent CD57+ CD8 T cells is associated with impaired immunocompetence after allogeneic hematopoietic stem cell transplantation. Blood. 2021;138(1):1807. 10.1182/blood-2021-153133.

[100] Delval L, Hantute-Ghesquier A, Sencio V, Flaman JM, Robil C, Angulo FS, Lipskaia L, Çobanoğlu O, Lacoste A-S, Machelart A, Danneels A, Corbin M, Deruyter L, Heumel S, Idziorek T, Séron K, Sauve K, Bongiovanni A, Prévot V, Wolowczuk I, Belouzard S, Saliou J-M, Gosset P, Bernard D, Rouillé Y, Adnot S, Duterque-Coquillaud M, Trottein F. Removal of senescent cells reduces the viral load and attenuates pulmonary and systemic inflammation in SARS-CoV-2-infected, aged hamsters.Nat Aging. 2023;3: 829–845. 10.1038/s43587-023-00442-w. 10.1038/s43587-023-00442-w PMC10353934 37414987

[101] Vazquez-Alejo E, Espinar-Buitrago MS, Genebat M, Calderón M, Pérez-Cabeza G, Magro-Lopez E, et al. Persistent exhausted T-cell immunity after severe COVID-19: 6-month evaluation in a prospective observational study. J Clin Med. 2023;12:3539. 10.3390/jcm12103539. 37240647 10.3390/jcm12103539 PMC10219183

[102] Chavda VP, Chhabria MT, Apostolopoulos V. Aged population and immunocompromised patients: impact on SARS-CoV-2 variants and treatment outcomes. Biologics. 2022;2:165–70. 10.3390/biologics2030013.

[103] Im KI, Kim N, Lee J, Oh UH, Lee HW, Lee DG, et al. SARS-CoV-2-specific T-cell as a potent therapeutic strategy against immune evasion of emerging COVID-19 variants. Int J Mol Sci. 2024;25:10512. 10.3390/ijms251910512. 39408840 10.3390/ijms251910512 PMC11477143

