# Supplementary figures and images for: Adoptive T-cell therapies for persistent COVID-19 in immunocompromised patients: Comparison of IFN-γ virus-specific T-cell therapy and CD45RA+ T-cell depleted donor lymphocyte infusion
Source: GeroScience. 2026 Jan 12;48(3):3755–87. doi: 10.1007/s11357-025-02050-5 (PMC13356011; doi:10.1007/s11357-025-02050-5)

A

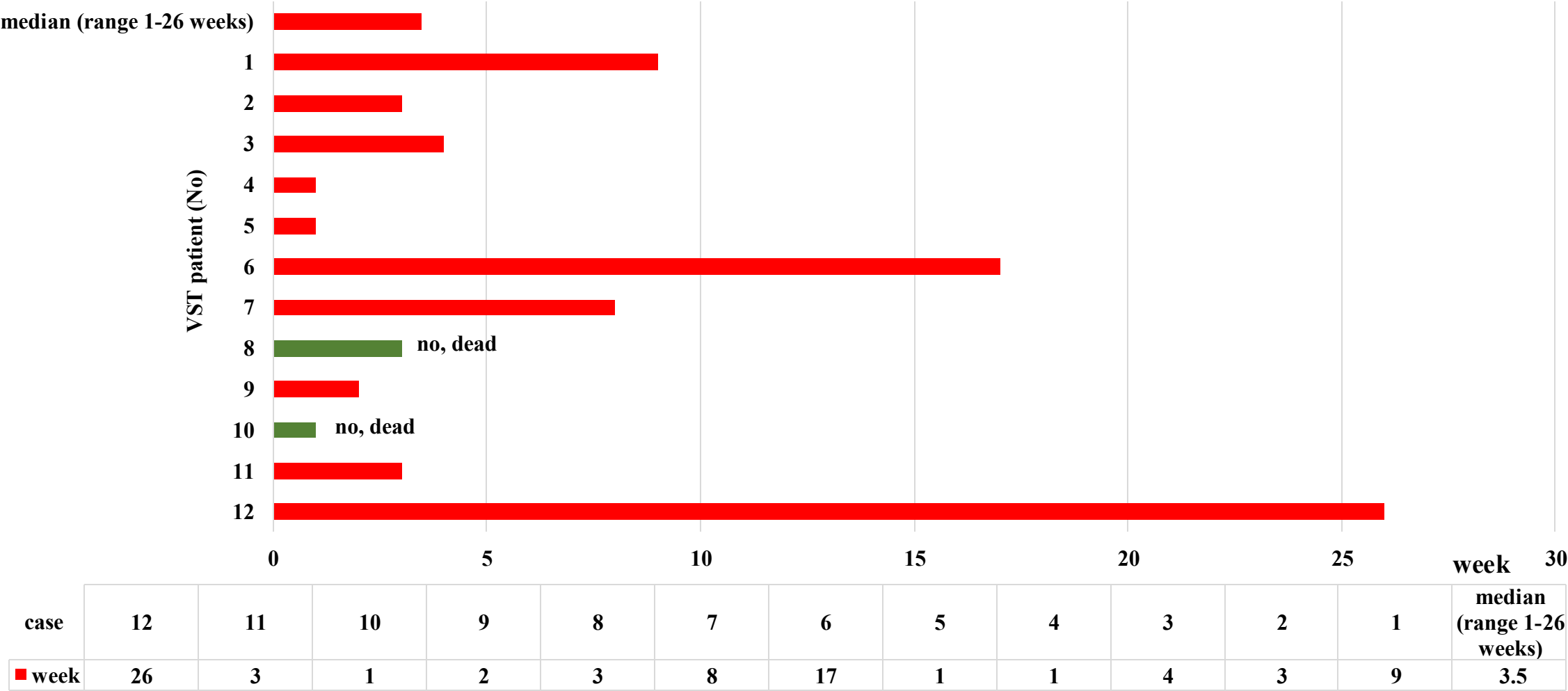

Supplement: Supplementary file 1 — Nasopharyngeal PCR clearance after SARS-CoV-2 specific IFN-γ CCS VST and CD45RA+ TCD DLI therapy, A: Nasopharyngeal PCR clearance after IFN-γ CCS VST. B: Nasophranygeal PCR clearance after CD45RA+ TCD DLI. Abbreviations: PCR: polymerase chain reaction; IFN-γ CCS: interferon-γ cytokine capture system; VST: virus-specific T-cells; TCD: T-cell depletion; DLI: donor memory T-cell infusion Note: In the CD45RA+ TCD DLI group, case 7 with persistent SARS-CoV-2 positivity and poor graft function after the 1 st third-party cryopreserved CD45RA+ TCD DLI received CD34+ positively selected and CD45RA+ depleted T-cell booster from her original haploidentical donor. (PDF 41.3 KB) [file 11357_2025_2050_MOESM1_ESM.pdf]

B

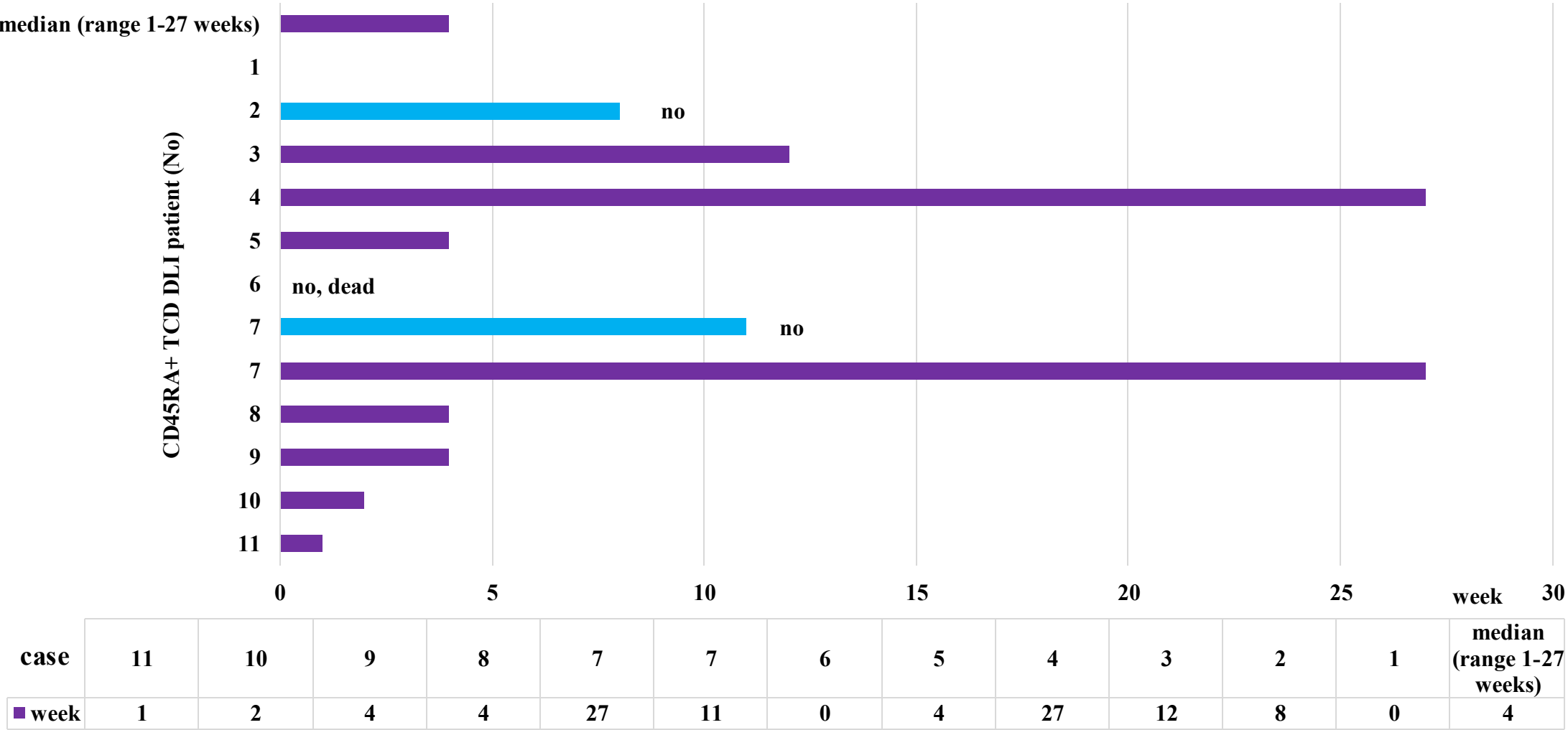

Supplement: Supplementary file 2 — (PDF 42.2 KB) [file 11357_2025_2050_MOESM2_ESM.pdf]

D

CD8+CD45RA+ naive T-cells

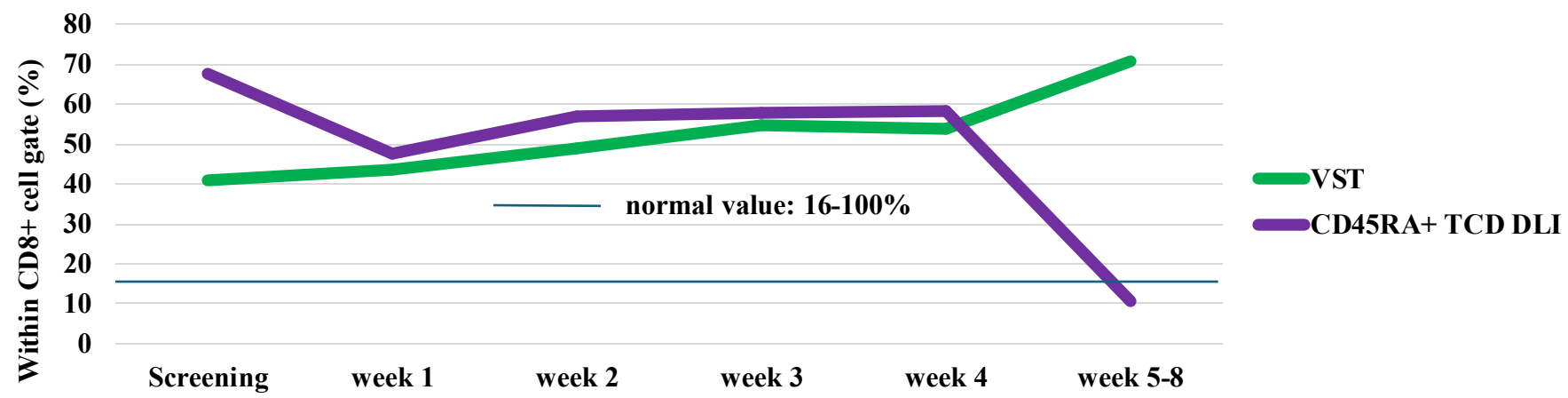

|                 |       |       |       |       |       |       |
|-----------------|-------|-------|-------|-------|-------|-------|
| VST             | 41.06 | 43.7  | 48.8  | 54.89 | 54    | 70.71 |
| CD45RA+ TCD DLI | 67.52 | 47.8  | 57.12 | 57.68 | 58.4  | 10.61 |
| p value         | 0.08  | 0.912 | 0.327 | 0.056 | 0.412 | 0.019 |

CD8+CD45RO+ memory T-cells

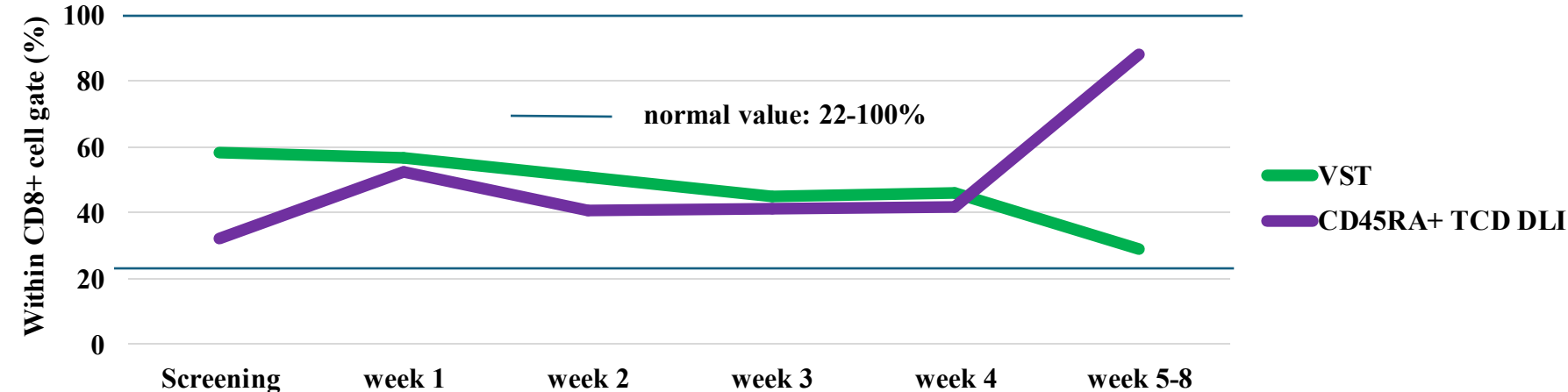

|                 |       |       |       |       |       |       |
|-----------------|-------|-------|-------|-------|-------|-------|
| VST             | 58.19 | 56.6  | 50.9  | 45.05 | 46.35 | 29.02 |
| CD45RA+ TCD DLI | 32.48 | 52.4  | 40.64 | 41.56 | 41.6  | 88.34 |
| p value         | 0.417 | 0.289 | 0.105 | 0.289 | 0.659 | 0.007 |

Supplement: Supplementary file 6 — (PDF 55.3 KB) [file 11357_2025_2050_MOESM6_ESM.pdf]

D

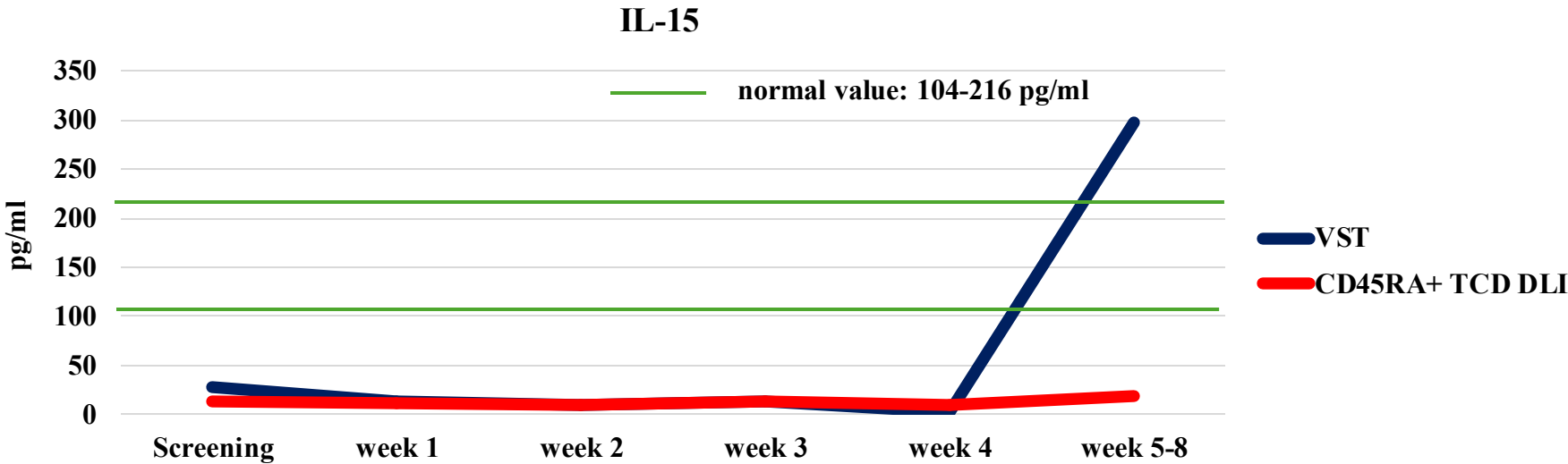

|                 |       |       |       |       |       |        |
|-----------------|-------|-------|-------|-------|-------|--------|
| VST             | 28.05 | 13.41 | 9.8   | 12.67 | 3     | 298.42 |
| CD45RA+ TCD DLI | 13.6  | 10.56 | 9.42  | 13.6  | 10.55 | 19.39  |
| p value         | 0.569 | 0.659 | 0.337 | 0.522 | 0.082 | n.a.   |

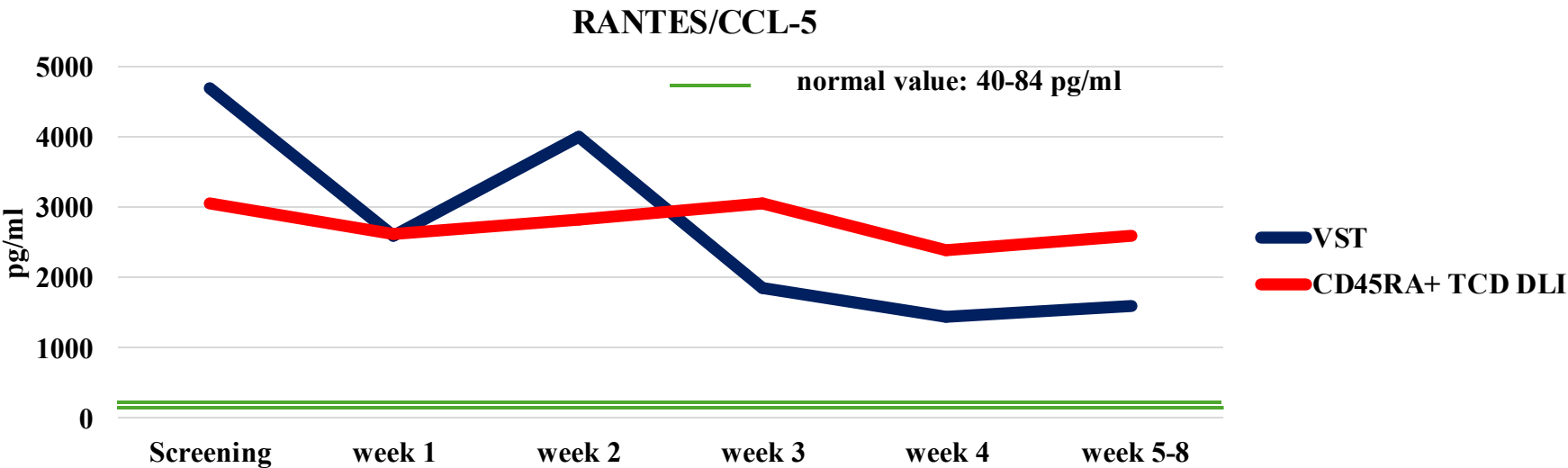

|                 |         |         |         |        |         |         |
|-----------------|---------|---------|---------|--------|---------|---------|
| VST             | 4698.42 | 2599.42 | 4019    | 1848.1 | 1448.7  | 1603.53 |
| CD45RA+ TCD DLI | 3049.5  | 2611.54 | 2827.97 | 3049.5 | 2387.06 | 2589.74 |
| p value         | 0.177   | 0.704   | 0.897   | 0.352  | 0.424   | n.a.    |

Supplement: Supplementary file 10 — (PDF 51.1 KB) [file 11357_2025_2050_MOESM10_ESM.pdf]

F

MIP-1α/CCL3

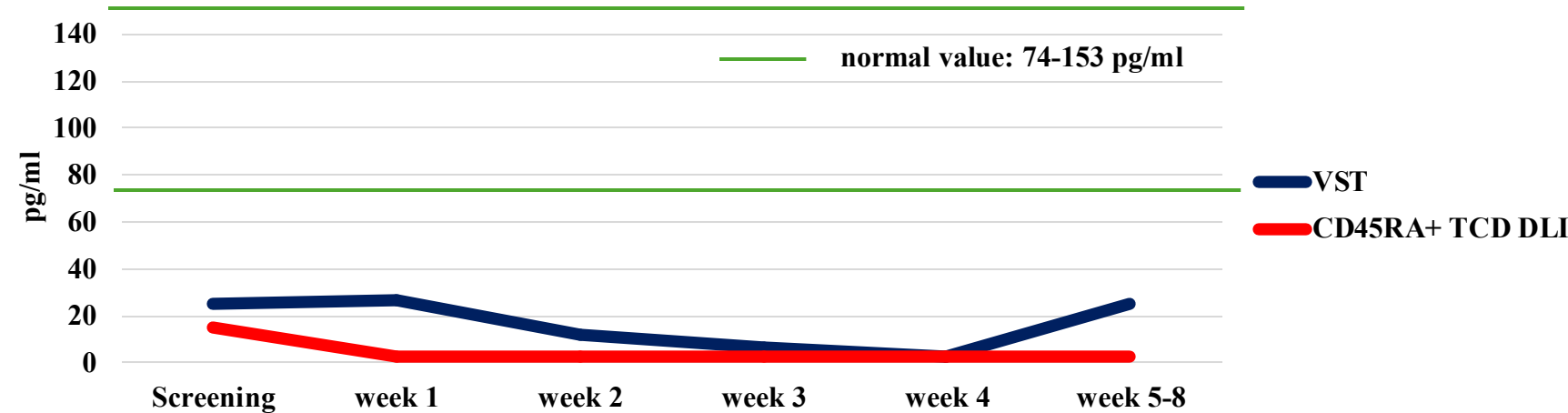

|                 |       |       |       |       |       |       |
|-----------------|-------|-------|-------|-------|-------|-------|
| VST             | 25.38 | 26.67 | 12.34 | 6.93  | 3     | 25.37 |
| CD45RA+ TCD DLI | 15.01 | 3     | 3     | 3     | 3     | 3     |
| p value         | 0.689 | 0.891 | 0.042 | 0.826 | 0.968 | n.a.  |

MCP-1/CCL2

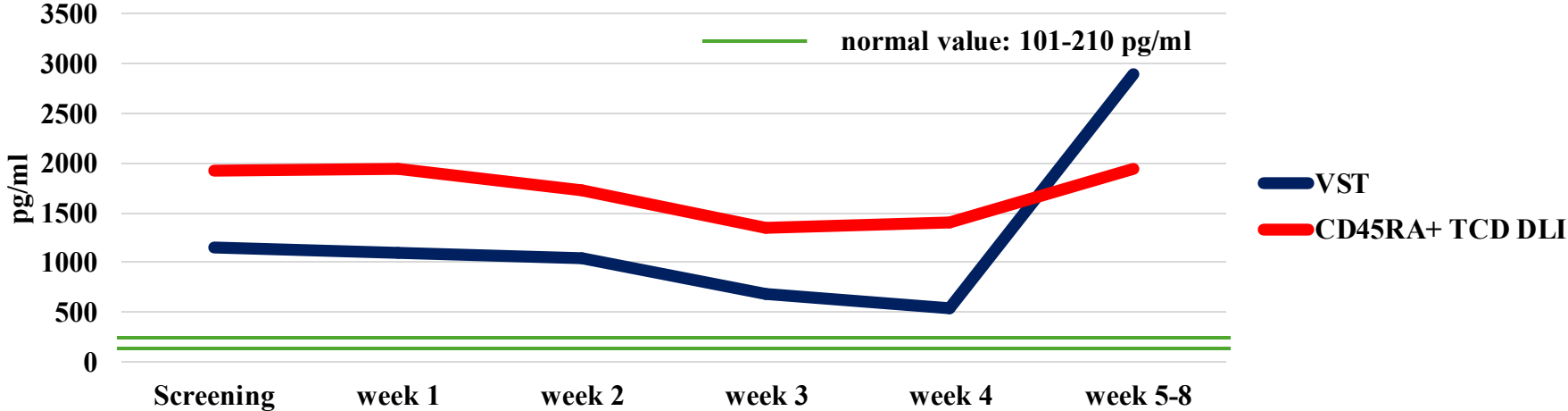

|                 |         |         |         |         |         |         |
|-----------------|---------|---------|---------|---------|---------|---------|
| VST             | 1149.89 | 1102.11 | 1038.32 | 679.32  | 536.56  | 2904.54 |
| CD45RA+ TCD DLI | 1935.15 | 1942.38 | 1728.44 | 1342.73 | 1397.41 | 1939.73 |
| p value         | 0.881   | 0.332   | 0.337   | 0.347   | 0.659   | n.a.    |

Supplement: Supplementary file 12 — (PDF 58.5 KB) [file 11357_2025_2050_MOESM12_ESM.pdf]
