# Supplementary material for: Adoptive T-cell therapies for persistent COVID-19 in immunocompromised patients: Comparison of IFN-γ virus-specific T-cell therapy and CD45RA+ T-cell depleted donor lymphocyte infusion
Source: GeroScience. 2026 Jan 12;48(3):3755–87. doi: 10.1007/s11357-025-02050-5 (PMC13356011; doi:10.1007/s11357-025-02050-5)
Supplement: Supplementary file 3 — Characterization of Peripheral Blood Lymphocyte Subpopulations by Flow Cytometry Following Adoptive T-Cell Therapy: IFN-γ CCS VST Versus CD45RA+ TCD DLI. A: Statistical comparisons of lymphocyte subpopulations percentages for both cohorts. B: Changes in T-cells and NK-cells. C: Changes in CD4+ and CD8+ naive T-cell compartments. D: Changes in CD4+ and CD8+ memory T-cell compartments. Note: yellow background: decreased value; pale blue background: normal value; pink background: elevated value. Abbreviations: VST: virus-specific T- cells; IFN-γ CCS: interferon-γ cytokine capture system; TCD: T-cell depleted; DLI: donor lymphocyte infusion; T-reg: regulatory T-cell. (PDF 125 KB) [file 11357_2025_2050_MOESM3_ESM.pdf]

A

Time intervals from SARS-CoV-2 T cell therapy	Screening			Week 1			Week 2			Week 3			Week 4			Week 5-8		
	VST	CD45 RA+ TCD	*P*	VST	CD45 RA+ TCD	*P*	VST	CD45R A+ TCD	*P*	VST	CD45 RA+ TCD	*P*	VST	CD45 RA+ TCD	*P*	VST	CD45 RA+ TCD	*P*
**Normal value within the lymphoid gate (%)**																		
CD3+ T-cells (55-83)	83.12	59.78	0.347	76.87	60.80	0.045	76.69	69.78	0.952	75.87	66.27	0.184	73.47	85.18	0.294	69.24	46.08	0.772
CD8+ T-cells (10-39)	50.36	39.92	0.347	49.43	29.89	0.084	48.21	36.36	0.617	42.04	31.25	0.719	41.61	43.07	0.617	38.33	24.34	0.226
CD4+ T-cells (28-57)	23.94	15.72	0.503	20.73	15.26	0.51	21.36	15.25	0.624	24.82	13.68	0.094	21.01	14.99	0.749	24.76	14.46	0.289
NK cells (7-31)	15.56	33.28	0.522	20.10	30.80	0.857	21.77	27.46	0.347	23.20	31.82	0.139	21.08	14.54	0.646	19.73	29.99	0.549
CD19+ B-cells (6-19)	0.0	0.08	0.779	0.07	0.00	0.242	0.04	0.04	0.984	0.09	0.00	0.659	0.00	0.04	0.802	0.37	0.28	0.818
**Normal value within the CD3+ T-cell gate (%)**																		
TCRαβ (36-98)	95.55	96.40	0.119	96.56	96.14	0.490	97.90	95.09	0.638	97.84	98.33	0.271	97.29	98.60	0.849	97.50	85.29	0.384
TCRγδ (0,8-11)	4.35	3.60	0.262	3.15	3.86	0.208	2.00	4.92	0.128	1.70	1.60	0.337	2.71	1.40	0.438	2.30	14.71	0.197
CD3+CD25+ T-cells (>1)	5.19	4.62	0.624	3.6	3.53	0.459	6.05	7.57	0.162	13.42	5.03	0.490	13.25	5.42	0.267	9.90	6.99	0.472
CD3+HLA-DR+ T-cells (2-12%)	30.20	12.17	0.624	25.2	8.70	0.697	22.40	12.32	0.298	17.40	9.00	0.424	25.38	15.56	0.555	20.04	28.00	0.139
**Normal value within the CD4+ T-cell gate (%)**																		
CD4+CD45RA+ naive T-cells (16-100)	15.30	10.71	0.133	13.84	9.63	0.674	15.80	6.00	0.857	14.55	9.01	0.317	13.60	15.66	0.646	18.98	3.40	0.603
CD4+CD45RO+ memory T-cells (19-100)	84.75	89.29	0.267	85.47	90.37	0.478	84.30	88.68	0.099	85.40	90.99	0.384	86.48	84.35	0.764	81.12	96.28	0.056
**Normal value within the CD8+ T-cell gate (%)**																		
CD8+CD45RA+ naive T-cells (16-100)	41.06	67.52	0.08	43.7	47.80	0.912	48.80	57.12	0.327	54.89	57.68	0.056	54.00	58.40	0.412	70.71	10.61	0.019
CD8+CD45RO+ memory T-cells (22-100)	58.19	32.48	0.417	56.6	52.04	0.289	50.90	40.64	0.105	45.05	41.56	0.289	46.35	41.60	0.659	29.02	88.34	0.007
T-reg of total nucleated cells	0.15	0.15	0.704	0.286	0.14	0.802	0.21	0.11	0.968	0.28	0.23	0.936	0.11	0.07	0.696	0.20	0.09	0.877

Colour scales: Normal value Elevated value Decreased value
