# Supplementary material for: Adoptive T-cell therapies for persistent COVID-19 in immunocompromised patients: Comparison of IFN-γ virus-specific T-cell therapy and CD45RA+ T-cell depleted donor lymphocyte infusion
Source: GeroScience. 2026 Jan 12;48(3):3755–87. doi: 10.1007/s11357-025-02050-5 (PMC13356011; doi:10.1007/s11357-025-02050-5)
Supplement: Supplementary file 4 — (PDF 56.3 KB) [file 11357_2025_2050_MOESM4_ESM.pdf]

B

CD3+ T-cells

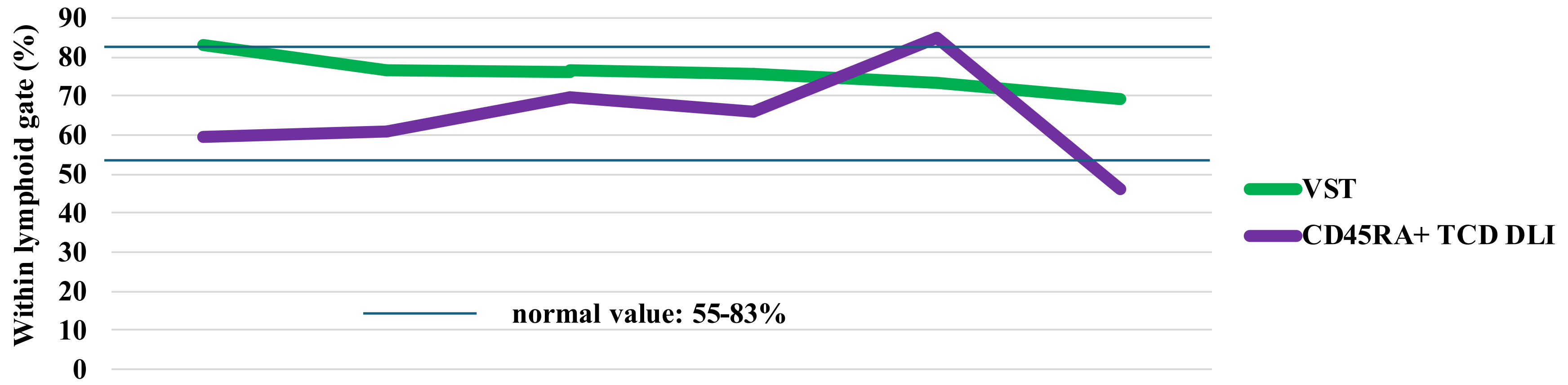

	Screening	week 1	week 2	week 3	week 4	week 5-8
VST	83.12	76.87	76.69	75.87	73.47	69.24
CD45RA+ TCD DLI	59.78	60.8	69.78	66.27	85.18	46.08
p value	0.347	0.045	0.952	0.184	0.294	0.772

NK cells

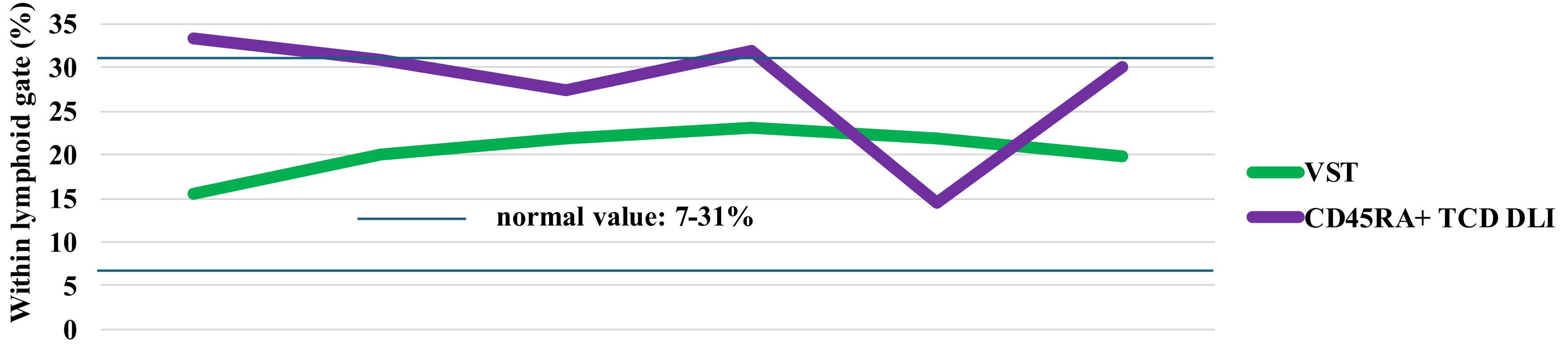

normal value: 7-31%

	Screening	week 1	week 2	week 3	week 4	week 5-8
VST	15.56	20.1	21.77	23.2	21.8	19.73
CD45RA+ TCD DLI	33.28	30.8	27.46	31.82	14.54	29.99
p value	0.522	0.857	0.347	0.139	0.646	0.549
