# Supplementary material for: Adoptive T-cell therapies for persistent COVID-19 in immunocompromised patients: Comparison of IFN-γ virus-specific T-cell therapy and CD45RA+ T-cell depleted donor lymphocyte infusion
Source: GeroScience. 2026 Jan 12;48(3):3755–87. doi: 10.1007/s11357-025-02050-5 (PMC13356011; doi:10.1007/s11357-025-02050-5)
Supplement: Supplementary file 5 — (PDF 54.9 KB) [file 11357_2025_2050_MOESM5_ESM.pdf]

C

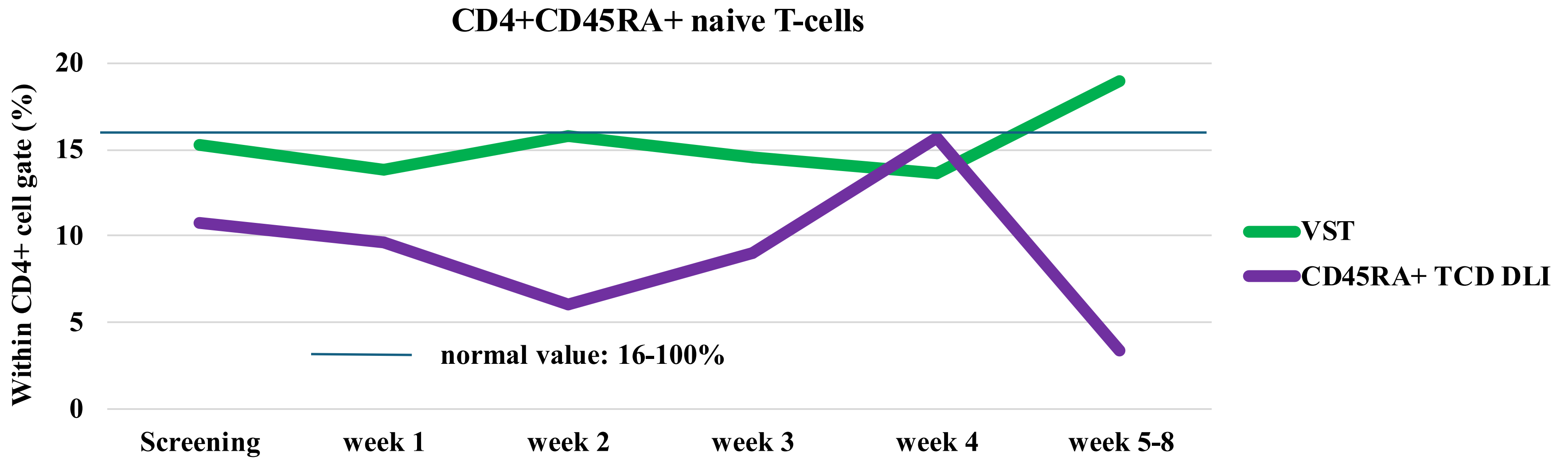

	Screening	week 1	week 2	week 3	week 4	week 5-8
VST	15.3	13.84	15.8	14.55	13.6	18.98
CD45RA+ TCD DLI	10.71	9.63	6	9	15.66	3.4
p value	0.133	0.674	0.857	0.317	0.646	0.603

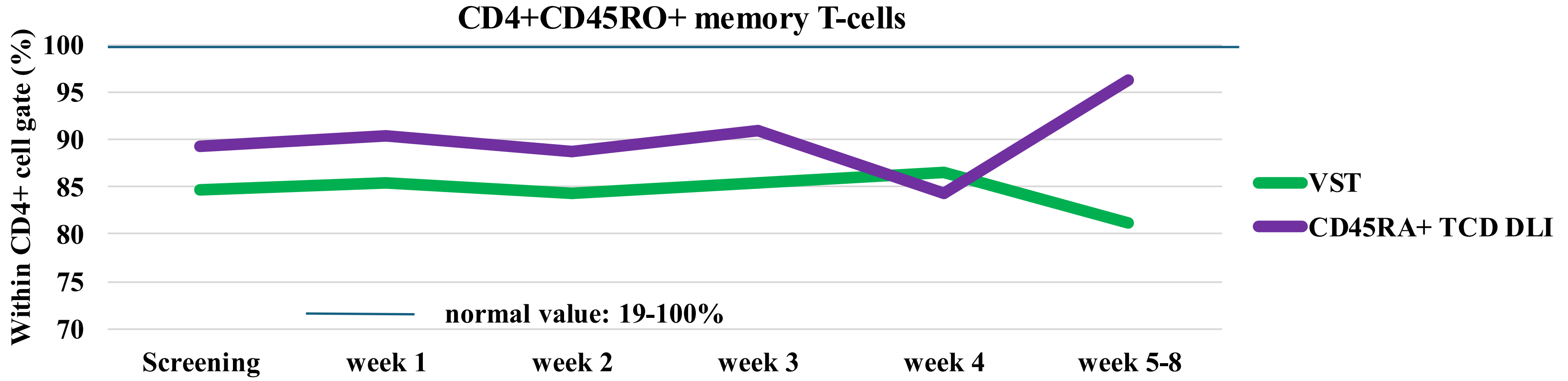

	Screening	week 1	week 2	week 3	week 4	week 5-8
VST	84.75	85.47	84.3	85.4	86.48	81.12
CD45RA+ TCD DLI	89.29	90.37	88.68	90.99	84.35	96.28
p value	0.267	0.478	0.099	0.384	0.764	0.056
