# Supplementary material for: Adoptive T-cell therapies for persistent COVID-19 in immunocompromised patients: Comparison of IFN-γ virus-specific T-cell therapy and CD45RA+ T-cell depleted donor lymphocyte infusion
Source: GeroScience. 2026 Jan 12;48(3):3755–87. doi: 10.1007/s11357-025-02050-5 (PMC13356011; doi:10.1007/s11357-025-02050-5)
Supplement: Supplementary file 7 — Comparison of changes in multicytokine and chemokine patterns following IFN-γ CCS VST versus CD45RA+ depleted DLI therapy. A: Statistical comparisons of cytokine and chemokine values for both cohorts. B: Changes in IL-5 and IL-6 levels. C: Changes in IL-8 and IL-10 levels. D: Changes in IL-15 and RANTES levels. E: Changes in IP-10 and IFNγ levels. F: Changes in MIP-1α and MCP-1 levels. Note: pale gray background: decreased value; yellow background: normal value; orange background: >1-<2x upper normal value; light blue background: >2-<5x upper normal value; pinke background: >5-<10x upper normal value; purple background: >10-<50x upper normal value; dark blue background: >50-<100x upper normal value; red background: >100x upper normal value. Abbreviations: VST: virus specific T-cell; IFN-γ CCS: interferon-γ cytokine capture system; CD45RA+ TCD DLI: CD45RA+ T-cell depleted donor lymphocyte infusion; IFN: interferon; IL: interleukin; RANTES: regulated upon activation, normal T-cell expressed and secreted; CCL-5: C-C motif ligand 5; MCP-1: monocyte chemoattractant protein-1; IP-10: interferonγ-induced protein 10 kDa; CXCL10: C-X-C motif chemokine ligand 10; TNF: tumor necrosis factor. (PDF 169 KB) [file 11357_2025_2050_MOESM7_ESM.pdf]

A

Time intervals from SARS-CoV-2 T cell therapy	Screening			Week 1			Week 2			Week 3			Week 4			Week 5-8		
	VST	CD45 RA+ TCD	*P*	VST	CD45 RA+ TCD	*P*	VST	CD45RA+ TCD	*P*	VST	CD45 RA+ TCD	*P*	VST	CD45 RA+ TCD	*P*	VST	CD45 RA+ TCD	*P*
**IFNα2 pg/ml** (208-432)	8.00	8.00	0.711	17.42	8.00	0.492	8.00	8.00	0.976	8.00	8.00	0.555	22.59	8.00	0.596	26.64	32.03	n.a.
**IFNγ** (22-45)	2.13	7.97	0.472	3.33	17.60	0.134	2.13	6.11	0.389	1.30	6.37	0.035	1.46	6.37	0.453	26.28	9.54	n.a.
**IL-1α** (140-291)	6.00	11.89	0.184	4.80	6.95	0.459	4.80	18.85	0.168	4.80	4.80	0.322	4.80	4.80	0.129	17.32	4.80	n.a.
**IL-1β** (45-94)	2.79	1.60	0.352	1.60	1.60	0.303	1.60	2.29	0.472	1.60	1.60	0.912	1.60	1.60	0.857	3.14	1.60	n.a.
**IL-2** (18-38)	0.64	0.64	0.920	0.64	0.64	0.624	0.64	0.64	0.984	0.64	0.64	0.984	0.64	0.64	0.936	0.96	0.64	n.a.
**IL-4** (19-40)	1.87	2.10	0.764	2.40	1.52	0.603	2.31	3.52	0.535	1.45	2.32	0.509	1.34	1.62	0.779	2.23	1.43	n.a.
**IL-5** (21-44)	3.02	7.84	0.177	8.33	8.07	0.271	11.76	10.27	0.834	5.92	4.21	0.675	3.03	5.55	0.552	56.14	69.20	n.a.
**IL-6** (20-42)	41.66	16.87	0.407	23.48	18.30	0.267	10.47	4.35	0.787	5.21	3.99	1.0	1.66	5.02	0.682	417.18	21.78	n.a.
**IL-8** (21-44)	21.65	29.68	0.203	28.70	16.59	0.889	27.49	20.41	0.435	11.14	20.07	0.984	7.24	9.04	1.0	121.21	25.98	n.a.
**IL-10** (87-181)	21.84	20.82	0.624	18.90	22.01	0.184	16.63	27.69	0.280	4.28	12.91	0.060	6.25	18.39	0.021	409.50	16.84	n.a.
**IL-12 (p70)** (103-214)	3.27	3.00	0.582	3.00	3.00	0.704	3.00	3.00	0.803	3.00	3.00	0.363	3.00	3.00	0.317	4.15	3.00	n.a.
**IL-13** (197-409)	6.40	7.87	0.682	6.40	6.40	0.757	6.40	8.28	0.447	6.40	9.91	0.099	6.41	6.65	0.490	21.95	6.40	n.a.
**IL-15** (104-216)	28.05	13.60	0.569	13.41	10.56	0.659	9.80	9.42	0.337	12.67	13.60	0.522	3.00	10.55	0.082	298.42	19.39	n.a.
**IL-17A** (40-82)	1.55	1.30	0.401	1.30	1.30	0.246	1.30	1.30	1.0	1.30	1.30	0.912	1.30	1.30	0.976	1.30	1.30	n.a.
**IP-10/ CXCL10** (67-139)	719.69	247.73	0.803	510.35	384.82	0.631	291.77	388.66	0.456	445.24	382.63	0.764	292.69	508.58	0.142	969.04	848.04	n.a.
**MCP-1/ CCL2** (101-210)	1149.89	1935.15	0.881	1102.11	1942.38	0.332	1038.32	1728.44	0.337	679.32	1342.73	0.347	536.56	1397.41	0.659	2904.54	1939.73	n.a.
**MIP-1α/CCL3** (74-153)	25.38	15.01	0.689	26.67	3.00	0.891	12.34	3.00	0.042	6.93	3.00	0.826	3.00	3.00	0.968	25.37	3.00	n.a.
**RANTES/CCL-5** (40-84)	4698.42	3049.50	0.177	2599.42	2611.54	0.704	4019.00	2827.97	0.897	1848.10	3049.50	0.352	1448.70	2387.06	0.424	1603.53	2589.74	n.a.
**TNFα** (123-256)	36.70	35.67	0.992	32.34	39.22	0.728	24.44	33.54	0.667	12.78	45.94	0.719	18.14	17.22	0.089	38.49	33.54	n.a.
**TNFβ** (53-111)	2.40	12.37	0.215	3.59	8.15	0.589	1.88	15.35	0.920	1.60	11.59	0.004	1.60	4.69	0.044	3.86	3.86	n.a.

Cytokine levels colour scales: Decreased value | Normal value | >1-<2x ULN | >2-<5x ULN | >5<10x ULN | >10-<50x ULN | >50-<100x ULN | >100x ULN
