# Supplementary material for: Adoptive T-cell therapies for persistent COVID-19 in immunocompromised patients: Comparison of IFN-γ virus-specific T-cell therapy and CD45RA+ T-cell depleted donor lymphocyte infusion
Source: GeroScience. 2026 Jan 12;48(3):3755–87. doi: 10.1007/s11357-025-02050-5 (PMC13356011; doi:10.1007/s11357-025-02050-5)
Supplement: Supplementary file 8 — (PDF 52.4 KB) [file 11357_2025_2050_MOESM8_ESM.pdf]

B

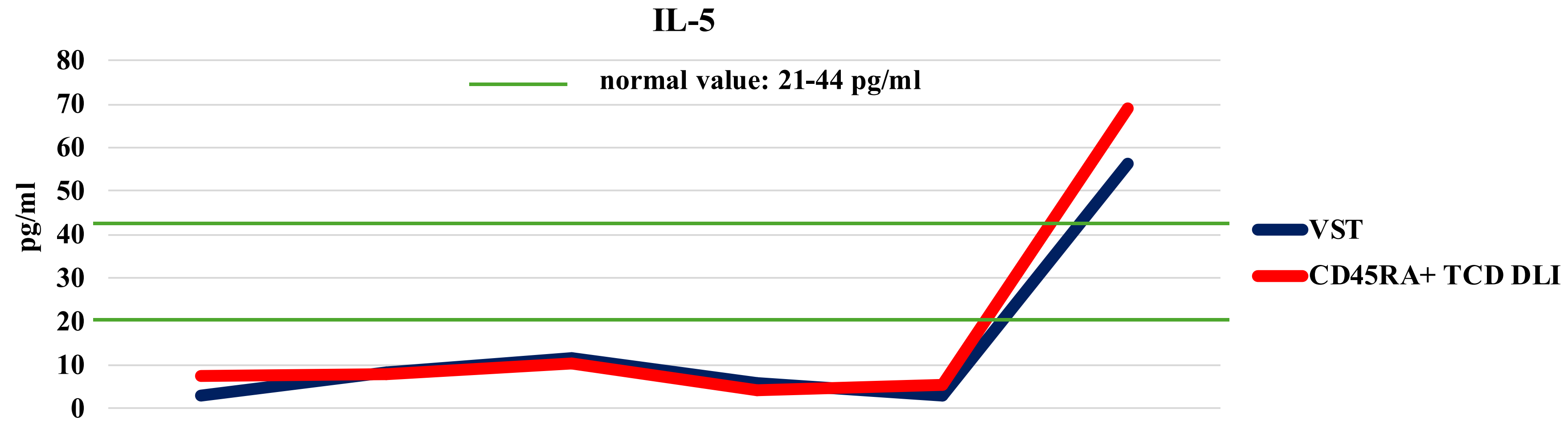

	Screening	week 1	week 2	week 3	week 4	week 5-8
VST	3.02	8.33	11.76	5.92	3.03	56.14
CD45RA+ TCD DLI	7.84	8.07	10.27	4.21	5.55	69.2
p value	0.177	0.271	0.834	0.675	0.552	n.a.

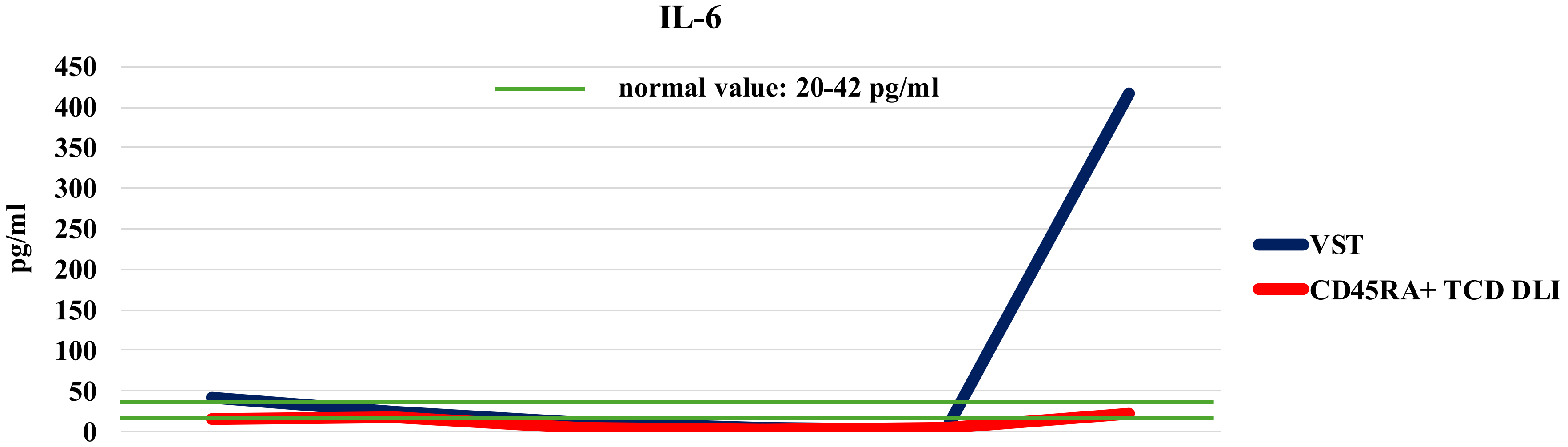

	Screening	week 1	week 2	week 3	week 4	week 5-8
VST	41.66	23.48	10.47	5.21	1.66	417.18
CD45RA+ TCD DLI	16.87	18.3	4.35	3.99	5.02	21.78
p value	0.407	0.267	0.787	1.0	0.682	n.a.
