# Supplementary material for: Adoptive T-cell therapies for persistent COVID-19 in immunocompromised patients: Comparison of IFN-γ virus-specific T-cell therapy and CD45RA+ T-cell depleted donor lymphocyte infusion
Source: GeroScience. 2026 Jan 12;48(3):3755–87. doi: 10.1007/s11357-025-02050-5 (PMC13356011; doi:10.1007/s11357-025-02050-5)
Supplement: Supplementary file 9 — (PDF 50.5 KB) [file 11357_2025_2050_MOESM9_ESM.pdf]

C

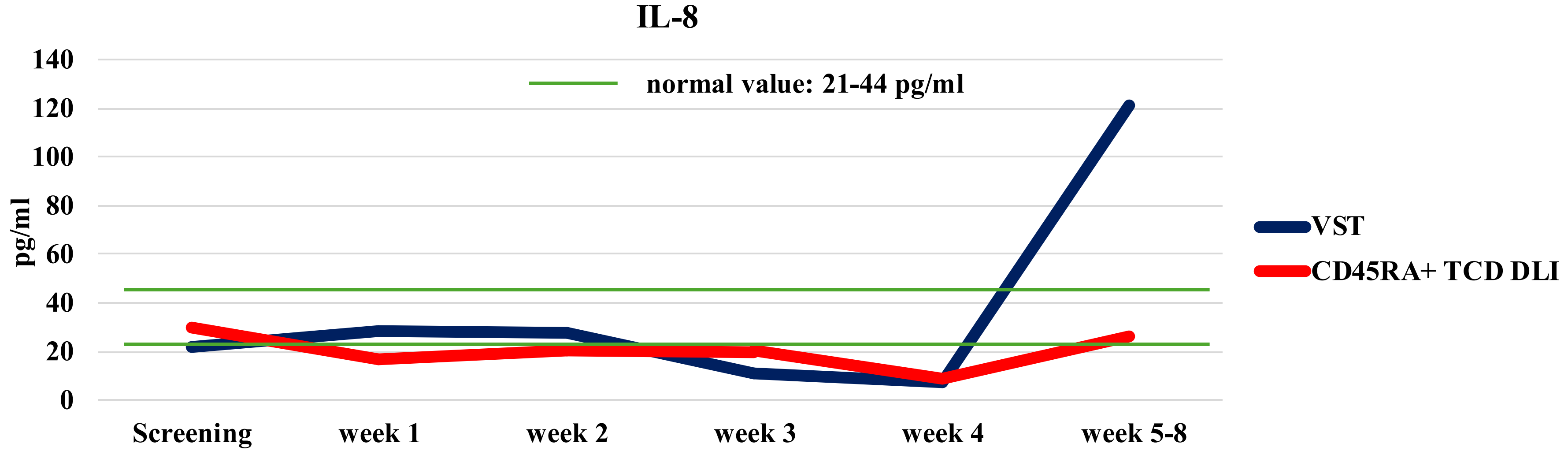

VST	21.65	28.7	27.49	11.14	7.24	121.21
CD45RA+ TCD DLI	29.68	16.59	20.41	20.07	9.04	25.98
p value	0.203	0.889	0.435	0.984	1.0	n.a.

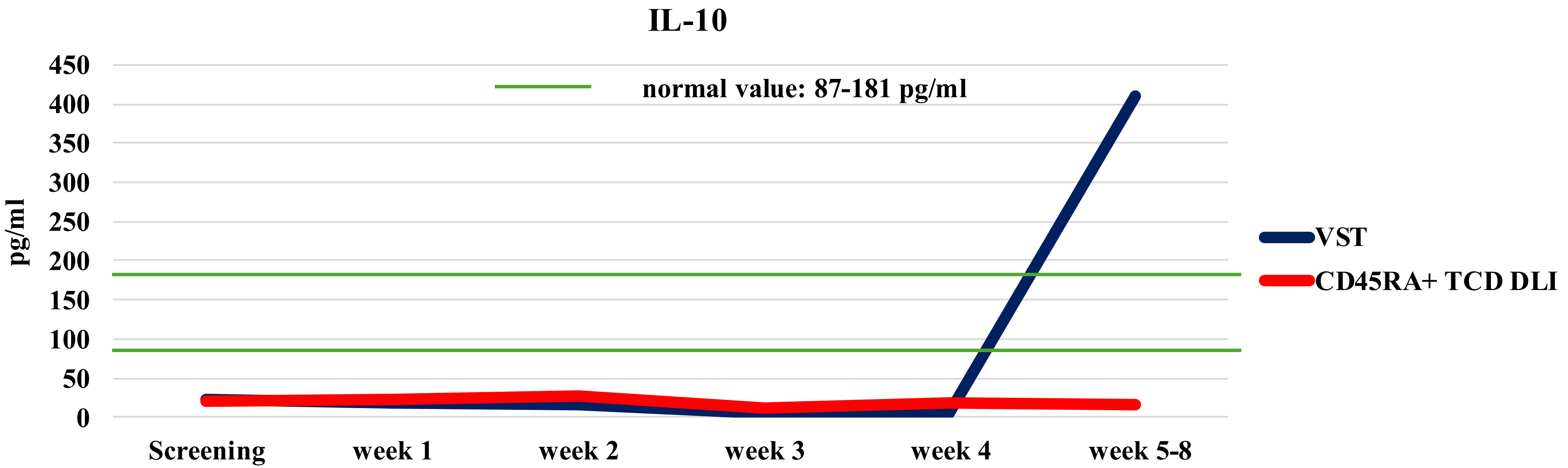

VST	21.84	18.9	16.63	4.28	6.25	409.5
CD45RA+ TCD DLI	20.82	22.01	27.69	12.91	18.39	16.84
p value	0.624	0.184	0.280	0.060	0.021	n.a.
