# Supplementary material for: Adoptive T-cell therapies for persistent COVID-19 in immunocompromised patients: Comparison of IFN-γ virus-specific T-cell therapy and CD45RA+ T-cell depleted donor lymphocyte infusion
Source: GeroScience. 2026 Jan 12;48(3):3755–87. doi: 10.1007/s11357-025-02050-5 (PMC13356011; doi:10.1007/s11357-025-02050-5)
Supplement: Supplementary file 11 — (PDF 59.9 KB) [file 11357_2025_2050_MOESM11_ESM.pdf]

E

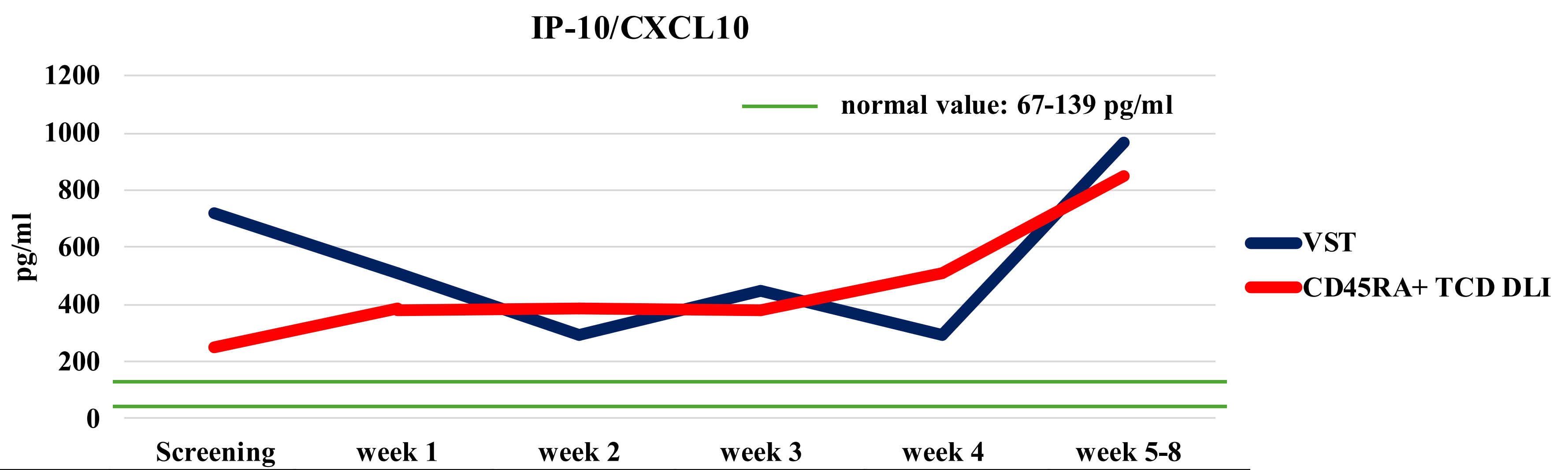

VST	719.69	510.35	291.77	445.24	292.69	969.04
CD45RA+ TCD DLI	247.3	384.82	388.66	382.63	508.58	848.04
p value	0.803	0.631	0.456	0.764	0.142	n.a.

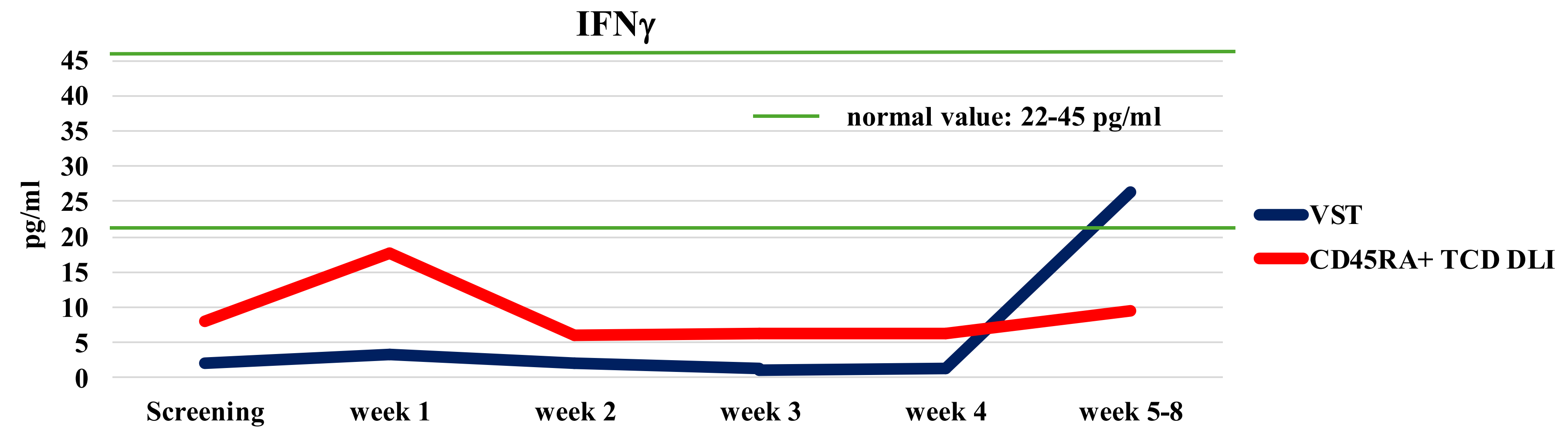

VST	2.13	3.33	2.13	1.3	1.46	26.28
CD45RA+ TCD DLI	7.97	17.6	6.11	6.37	6.37	9.54
p value	0.472	0.134	0.389	0.035	0.453	n.a.
