# Supplementary material for: Adoptive T-cell therapies for persistent COVID-19 in immunocompromised patients: Comparison of IFN-γ virus-specific T-cell therapy and CD45RA+ T-cell depleted donor lymphocyte infusion
Source: GeroScience. 2026 Jan 12;48(3):3755–87. doi: 10.1007/s11357-025-02050-5 (PMC13356011; doi:10.1007/s11357-025-02050-5)
Supplement: Supplementary file 13 — (DOCX 37.8 KB) [file 11357_2025_2050_MOESM13_ESM.docx]

**Supplementary Table 1.: Donor characteristics and composition of T-cell products, and the administered doses.**

**(A) Third-party SARS-CoV-2 virus specific T-cell product characteristics-processed by Miltenyi Prodigy system.**

**(B) Third-party SARS-CoV-2 CD45RA+ depleted donor lymphocyte infusion product characteristics-processed by Miltenyi CliniMACS Plus system.**

**A**

| **VST Case** | **1** | **2** | **3** | **4** | **5** | **6** | **7** | **8** | **9** | **10** | **11** | **12** | **median (range) or %** |
| --- | --- | --- | --- | --- | --- | --- | --- | --- | --- | --- | --- | --- | --- |
| **Donor: year, gender,** | **42-M** | **32-F** | **30-M** | **39-M** | **30-M (donor of case 3)** | **35-F** | **47-F** | **22-M** | **32-F (donor of case 2)** | **23-M** | **41-F** | **37-F** | **34 (22-47) M 50%, F 50%** |
| **Donor type** | **third-party** | **third-party** | **third-party** | **third-party** | **third-party** | **third-party** | **third-party** | **third-party** | **third-party** | **third-party** | **third-party** | **third-party** | **third- party 100%** |
| **HLA-allele matching** | **4/6** | **4/6** | **4/6** | **3/6** | **1/6** | **1/6** | **1/8** | **1/6** | **1/6** | **3/6** | **2/6** | **1/6** | **2/6 (1/8-4/6)**  **4/6 25%**  **3/6 16.7%**  **2/6 8.3%**  **1/6 41.7%**  **1/8 8.3%** |
| **Donor COVID-19 state** | **C** | **V** | **C+V** | **C** | **C+V** | **C** | **V** | **C+V** | **V** | **C+V** | **V** | **V** | **C 25%, C+V 33.3%, V 41.7%** |
| **Donor SARS-CoV-2 specific CD4+ IFNγ+ T-cells within CD4+ T-cell gate (%)** | **0.121** | **0.031** | **0.034** | **0.028** | **0.034** | **0.050** | **0.049** | **0.04** | **0.031** | **0.020** | **0.147** | **0.020** | **0.034% (range 0.02-0.147%)** |
| **Donor SARS-CoV-2 specific CD8+ IFNγ+ T-cells within CD8+ T-cell gate (%)** | **0.161** | **0.049** | **0.509** | **0.217** | **0.509** | **0.067** | **0.145** | **0.072** | **0.049** | **0.067** | **0.184** | **0.082** | **0.114% (range 0.049-0.509%)** |
| **Prodigy VST end product composition: Target (positive) fraction** | | | | | | | | | | | | | |
| **PBMC (x10^6^)** | **2.37** | **3.26** | **6.33** | **5.99** | **NA** | **3.22** | **5.29** | **4.23** | **NA** | **6.03** | **3.08** | **3.23** | **3.8 (2.4-6.33)** |
| **CD3+ T-cells (%)** | **67.713** | **63.228** | **76.345** | **55.02** | **NA** | **55.06** | **66.8** | **58.86** | **NA** | **8.98** | **56.94** | **25.83** | **57.9 (9.0-76.4)** |
| **CD3+ T-cells (x10^6^)** | **1.60** | **2.06** | **4.83** | **3.3** | **NA** | **1.77** | **3.54** | **2.49** | **NA** | **0.54** | **1.75** | **0.83** | **1.91 (0.54-4.83)** |
| **CD4+ T-cells (%)** | **53.241** | **59.633** | **9.023** | **27.93** | **NA** | **51.96** | **60.01** | **51.53** | **NA** | **79.88** | **75.6** | **57.54** | **55.39 (9.02-79.8)** |
| **CD4+ T-cells (x10^6^)** | **0.854** | **1.21** | **0.436** | **0.92** | **NA** | **0.922** | **2.12** | **1.28** | **NA** | **0.43** | **1.33** | **0.49** | **0.92 (0.43-2.12)** |
| **CD8+ T-cells (%)** | **44.10** | **39.14** | **88.58** | **68.75** | **NA** | **44.44** | **36.85** | **45.93** | **NA** | **12.85** | **22.87** | **34.56** | **41,62 (12.85-88.58)** |
| **CD8+ T-cells (x10^6^)** | **0.708** | **0.807** | **4.28** | **0.22** | **NA** | **0.788** | **1.3** | **1.14** | **NA** | **0.069** | **0.401** | **0.28** | **0.74 (0.069-4.28)** |
| **CD4+IFNγ+ T-cells (x10^6^)** | **0.715** | **0.963** | **0.279** | **0,55** | **NA** | **0.72** | **0.69** | **1.06** | **NA** | **0.43** | **0.708** | **0.27** | **0.699 (0.27-1.06)** |
| **IFNγ+ T-cells purity within CD4+ T-cells (%)** | **83.71** | **79.65** | **64** | **60.38** | **NA** | **78.15** | **67.42** | **82.8** | **NA** | **52.14** | **53.39** | **42.22** | **65.71 (42.22-83.71)** |
| **CD8+IFNγ+ T-cells (x10^6^)** | **0.599** | **0.616** | **4.11** | **2.26** | **NA** | **0.685** | **0.29** | **0.98** | **NA** | **0.017** | **0.26** | **0.096** | **0.607 (0.017-4.11)** |
| **IFNγ+ T-cells purity within CD8+ T-cells (%)** | **84.61** | **76.39** | **95.98** | **92.45** | **NA** | **86.91** | **77.22** | **86.25** | **NA** | **25** | **66.22** | **66.79** | **80.5 (25-95.98)** |
| **recipient body weight (kg)** | **75** | **80** | **95** | **42** | **94** | **95** | **70** | **70** | **80** | **58** | **95** | **52** | **77.5 (42-95)** |
| **End product non-IFNγ producing cells (x10^3^/kg)** | **3.308** | **5.454** | **3.463** | **12.76** | **0.81** | **3.20** | **14.11** | **5.39** | **5.46** | **4.46** | **10.25** | **7.16** | **5.42 (0.81-12.76)** |
| **End product non-IFNγ producing CD4+ cells (x10^3^/kg)** | **1.9** | **3.1** | **1.7** | **8.7** | **0.4** | **2.1** | **9.9** | **3.1** | **3.1** | **3.6** | **6.5** | **5.3** | **3.1 (0.4-9.9)** |
| **End product non-IFNγ producing CD8+ cells (x10^3^/kg)** | **1.4** | **2.4** | **1.8** | **4.1** | **0.41** | **1.1** | **4.2** | **2.2** | **2.4** | **0.9** | **1.4** | **1.8** | **2 (0.41-4.2)** |
| **End product IFNγ producing cells (x10^3^/kg)** | **17.52** | **19.738** | **46.2** | **61.57** | **10.83** | **14.78** | **34.85** | **29.21** | **19.78** | **4.17** | **10.25** | **7.57** | **18.63 (4.17-61.57)** |
| **End product IFNγ producing CD4+ cells (x10^3^/kg)** | **9.5** | **12.0** | **2.9** | **11.8** | **0.7** | **7.6** | **20.4** | **15.1** | **12.1** | **3.9** | **7.5** | **3.9** | **8.55 (0.7-20.4)** |
| **End product IFNγ producing CD8+ cells (x10^3^/kg)** | **8.0** | **7.7** | **43.3** | **49.8** | **10.1** | **1.1** | **14.4** | **14.1** | **7.7** | **0.3** | **2.8** | **3.7** | **7.85 (0.3-49.8)** |
| **1. VST IFNγ producing cells dose (x10^3^/kg)** | **5 f** | **5 f** | **10 f** | **61.57 c** | **10.8 c** | **14.78 f** | **34..85 f** | **29.21 f** | **19.78 c** | **4.17 f** | **10.25 f** | **7.16 f** | **17.28 (5-61.57)**  **f 75%**  **c 25%** |
| **2. VST IFNγ producing cells dose (x10^3^/kg)** | **5 c** | **5 c** | **10 c** | **ND** | **ND** | **ND** | **ND** | **ND** | **ND** | **ND** | **ND** | **ND** | **5 (5-10)**  **c 100%** |

**B**

| **CD45RA+ TCD DLI Case** | **1** | **2**^*^ | **3** | **4** | **5** | **6** | **7** | **7^Ψ^** | **8** | **9**^°^ | **10** | **11** | **median (range) or %** |
| --- | --- | --- | --- | --- | --- | --- | --- | --- | --- | --- | --- | --- | --- |
| **Donor: year, gender,** | **46-F** | **63-M** | **68-F** | **32-M** | **63-M (donor of case 2)** | **63-M (donor of case 2)** | **46-F (donor of case 1)** | **20-M** | **64-F** | **57-F** | **57-F (donor of case 9)** | **57-F (donor of case 9)** | **57 (20-68) M 36%, F 64%** |
| **Donor type** | **third-party** | **2 haplo** | **third-party** | **third-party** | **third-party** | **third-party** | **third-party** | **haplo** | **third-party** | **haplo** | **third-party** | **third-party** | **third- party 75%, haplo 25%** |
| **HLA-allele matching** | **2/6** | **2x3/6** | **1/6** | **1/6** | **1/6** | **1/6** | **1/6** | **3/6** | **1/6** | **3/6** | **1/6** | **1/6** | **1/6 (1/6-3/6) 3/6 25%**  **2/6 8.3%**  **1/6 66.7%** |
| **Donor COVID-19 state** | **V** | **C** | **V** | **V** | **C** | **C** | **V** | **C** | **V** | **NA** | **NA** | **NA** | **C 33.3%, V 41.7%, NA 25%** |
| **Donor SARS-CoV-2 specific CD4+ IFNγ+ T-cells within CD4+ T-cell gate (%)** | **0.063** | **1. 0.009 2. 0.003** | **0.006** | **0.017** | **0.009** | **0.009** | **0.063** | **ND** | **0.019** | **0.0027** | **0.0027** | **0.0027** | **0.009% (range 0.003-0.063%)** |
| **Donor SARS-CoV-2 specific CD8+ IFNγ+ T-cells within CD8+ T-cell gate (%)** | **0.007** | **1. 0.021 2. 0.007** | **0.016** | **0.215** | **0.021** | **0.021** | **0.007** | **ND** | **0.009** | **0.00** | **0.00** | **0.00** | **0.009% (range 0.00-0.215%)** |
| **CliniMACS Plus CD45RA+ TCD end product composition: Target (positive) fraction** | | | | | | | | | | | | | |
| **PBMC (x10^9^)** | **10.2** | **18.2** | **2.5** | **2.6** | **NA** | **NA** | **NA** | **4.4** | **11.2** | **9.0** | **NA** | **NA** | **9.0 (2.5-18.2)** |
| **CD3+ T-cells (%)** | **41** | **19** | **67** | **64** | **NA** | **NA** | **NA** | **47** | **25** | **67** | **NA** | **NA** | **47 (19-67)** |
| **CD4+/CD8+ T-cell ratio** | **7.2** | **8.31** | **3.43** | **10.4** | **NA** | **NA** | **NA** | **16.7** | **12.14** | **26.7** | **NA** | **NA** | **10.4 (3.43-26.7)** |
| **CD3+CD45RA+ T-cells (%)** | **0.095** | **0.116** | **0.042** | **0.056** | **NA** | **NA** | **NA** | **0.00** | **0.027** | **0.10** | **NA** | **NA** | **0.056 (0.0-0.116)** |
| **CD3+CD45RA- T-cells (%)** | **99.91** | **99.88** | **99.96** | **99.94** | **NA** | **NA** | **NA** | **100** | **99.97** | **99.9** | **NA** | **NA** | **99.97 (99.88-100)** |
| **recipient body weight (kg)** | **91** | **74** | **62** | **76** | **74** | **80** | **49** | **49** | **53** | **57** | **50** | **50** | **59.5 (49-91)** |
| **End product CD3+ T-cells (x10^6^/kg)** | **1.0** | **1.0** | **1.0** | **1.0** | **1.0** | **1.0** | **1.0** | **1.0** | **1.0** | **1.0** | **1.0** | **1.0** | **1.0** |
| **End product CD4+ T-cells (x10^6^/kg)** | **1.0** | **1.0** | **1.0** | **1.0** | **1.0** | **1.0** | **0.7** | **1.0** | **1.0** | **1.0** | **1.0** | **1.0** | **1.0 (0.7-1.0)** |
| **End product CD3+ CD45RA+T-cells (x10^2^/kg)** | **9.8** | **11.9** | **1.4** | **5.6** | **1.2** | **1.2** | **4.2** | **0.0** | **2.7** | **10.6** | **3.2** | **0.0** | **2.95 (0.0-11.9)** |
| **1. CD3+CD45RA-TCD dose (x10^6^/kg)** | **1.03**  **f** | **1.023**  **f** | **1.056 c** | **1.0**  **f** | **1.01 c** | **1.01 c** | **1.0 c** | **1.03 f** | **0.99 f** | **1.05 f** | **1.06 c** | **1.03 c** | **1.03 (0.99-1.06)**  **f 50%**  **c 50%** |
| **2. CD3+CD45RA-TCD dose (x10^6^/kg)** | **ND** | **1.02 c** | **ND** | **ND** | **ND** | **ND** | **ND** | **ND** | **ND** | **ND** | **ND** | **ND** | **NA** |

Abbreviation: VST: virus specific T-cell; M: male; F: female; C: convalescent; V: vaccinated; PBMC: peripheral blood mononuclear cells; IFNγ: interferon-gamma, f: fresh; c: cryopreserved; NA: not applicable; TCD: T-cell depletion; DLI: donor lymphocyte infusion; Haplo: haploidentical donor; ND: not done.

^*^ case 2 underwent 2nd allogeneic haploidentical stem cell transplantation from his SARS-CoV-2 convalescent father using CD34+ positivily selected+CD45RA+ depleted T-cell graft

**^Ψ^** case 7 received a CD34+ booster and CD45RA+ depleted donor lymphocyte infusion from a haploidentical donor due to poor graft function and persistent SARS-CoV-2 positivity.

^°^ case 9 received CD45RA depleted donor memory T-cell infusion from her original stem cell donor.
