# Supplementary material for: Adoptive T-cell therapies for persistent COVID-19 in immunocompromised patients: Comparison of IFN-γ virus-specific T-cell therapy and CD45RA+ T-cell depleted donor lymphocyte infusion
Source: GeroScience. 2026 Jan 12;48(3):3755–87. doi: 10.1007/s11357-025-02050-5 (PMC13356011; doi:10.1007/s11357-025-02050-5)
Supplement: Supplementary file 14 — (DOCX 20.8 KB) [file 11357_2025_2050_MOESM14_ESM.docx]

**Supplementary Table 2: Microchimerism results after the administration of SARS-CoV-2 VST and CD45RA+ TCD DLI, as assessed by ddPCR. Microchimerism results: A: VST and B: CD45RA+ TCD DLI.**

**A**

|  | **ddDIP PCR result (VST donor % of total white blood cells)** | | | | |  |
| --- | --- | --- | --- | --- | --- | --- |
| **Case** | **After VST, Week 1** | **Week 2** | **Week 3** | **Week 4** | **Week 5** | **Examined on sorted T cells** |
| **1** | NIM | NIM | NIM | NIM | NIM | NA |
| **2** | 0% | 0% | 0% | 0% | ND | yes |
| **3^†^** | 0% | 0% | 0% | 0% | 0% | yes |
| **4** | 0% | 0% | 0% | 0% | 0% | no |
| **5** | ND | ND | ND | ND | ND | NA |
| **6** | 0% | 0% | 0% | 0% | ND | no |
| **7** | ND | 0% | 0% | 0% | ND | no |
| **8** | 0% | 0% | ND | 0% | ND | no |
| **9** | 0% | 0% | ND | ND | ND | yes |
| **10** | 0% | 0% | ND | ND | ND | yes |
| **11** | ND | 0% | 0% | 0% | ND | no |
| **12** | 0% | 0% | 0% | 0% | ND | no |

**B**

|  | **ddDIP PCR result (CD45RA+ TCD donor % of total white blood cells)** | | | | |  |
| --- | --- | --- | --- | --- | --- | --- |
| **Case** | **After CD45RA+ TCD DLI, Week 1** | **Week 2** | **Week 3** | **Week 4** | **Week 5** | **Examined on sorted T cells** |
| **1^†^** | 0% | 0% | 0% | 0% | ND | no |
| **3** | 0.754% | 0.209% | 0% | 0% | ND | no |
| **4** | 0% | 0% | 0% | 0% | ND | no |
| **5** | ND | ND | 0% | 0% | 0% | no |
| **6** | NA | NA | NA | NA | NA | NA |
| **7** | 0% | 0% | 0% | ND | ND | no |
| **8** | 0% | 0% | 0% | 0% | 0% | no |
| **10** | 0% | 0% | 0% | 0% | ND | no |
| **11** | NIM | NIM | NIM | NIM | NIM | NA |

**Abbreviations**: VST: virus-specific T-cells; TCD: T-cell depletion; DLI: donor memory T-cell infusion; STR: short tandem repeats; ddDIP: digital droplet deletion-insertion polymorphism; ND: not done; NIM: no informative marker; NA: not applicable.

† Case 3 in Table A and Case 1 in Table B refer to the same patient, who received VST for the initial SARS-CoV-2 infection and subsequently received CD45RA+ depleted DLI for a second infection.

Note: Within CD45RA+ TCD DLI group, case 2 underwent a second allogeneic haploidentical stem cell transplantation from his SARS-CoV-2 convalescent father, using a CD34+ positively selected and CD45RA+ depleted T-cell graft. Non take due to case 2 also underwent a third allogeneic haploidentical stem cell transplantation from his SARS-CoV-2 convalescent mother, using unmanipulated graft. Case 7 with persistent SARS-CoV-2 positivity and poor graft function after the 1st third-party cryopreserved CD45RA+ TCD DLI received CD34+ positively selected and CD45RA+ depleted T-cell booster from her original haploidentical donor. Case 9 received a CD45RA+ depleted donor memory T-cell infusion from her original SARS-CoV-2 convalescent stem cell donor.
